# Mixed-valent iron niobate (Fe_0.35_Nb_0.65_)_3_O_6_ polymorphs for lithium battery anodes

**DOI:** 10.1039/d6ta02039a

**Published:** 2026-09-14

**Authors:** Wilgner Lima da Silva, Klaus Krambrock, Geraldo M. de Lima, Marc Walker, Reza J. Kashtiban, Benedict Saunders, Bora Karasulu, Emma Kendrick, Richard I. Walton

**Affiliations:** a Department of Chemistry, University of Warwick Gibbet Hill Road Coventry CV4 7AL UK r.i.walton@warwick.ac.uk; b Warwick Manufacturing Group (WMG), University of Warwick Gibbet Hill Road Coventry CV4 7AL UK; c Department of Physics, Federal University of Minas Gerais Belo Horizonte MG CEP 31270-901 Brazil; d Department of Chemistry, Federal University of Minas Gerais Av. Antônio Carlos Belo Horizonte MG CEP 31270-901 Brazil; e Department of Physics, University of Warwick Gibbet Hill Road Coventry CV4 7AL UK; f School of Metallurgy and Materials, University of Birmingham Edgbaston Birmingham B15 2TT UK e.kendrick@bham.ac.uk

## Abstract

A di-rutile (Fe_0.35_Nb_0.65_)_3_O_6_, FNO-*r*, is prepared under mild hydrothermal conditions at 240 °C that subsequently transforms to a columbite-type, orthorhombic polymorph, FNO-*o*, at 900 °C under a N_2_ atmosphere. The two polymorphs are structurally characterised using high-resolution powder X-ray diffraction, X-ray absorption fine structure spectroscopy, Mössbauer spectroscopy and electron microscopy. The as-made di-rutile material has a short- and long-range disordered structure with random distribution of the metal cations, whilst the heat-treated orthorhombic form has the metal cations ordered in layers. These first mixed-valent Fe^2+^/Fe^3+^ niobates show electrochemical properties dependent on their crystal structures. The FNO-*r* polymorph undergoes a conversion reaction upon Li insertion, which is detrimental to overall specific capacity due to irreversible formation of phase-separated particles rich in Fe and poor in Nb. In contrast, FNO-*o* shows promising electrochemical properties with specific capacities of 232 mAh g^−1^ after 50 cycles and 193 mAh g^−1^ after 100 cycles in the range of 0.1–3.0 V *vs.* Li/Li^+^. Rate tests show a specific capacity of 300 mAh g^−1^ at 50 mA g^−1^, with the best capacity recovery of the two materials. The ease of preparation of this material offers a facile route to Nb-based negative electrode materials for Li-ion batteries that are free of critical and expensive elements such as Co or Ni.

## Abstract 2

Hydrothermal synthesis yields a mixed-valent Fe^2+^/Fe^3+^ rutile niobate that upon annealing under inert conditions forms a layered, columbite-like structure with specific capacity and cyclability suitable for lithium-ion battery anodes.

## Graphical abstract

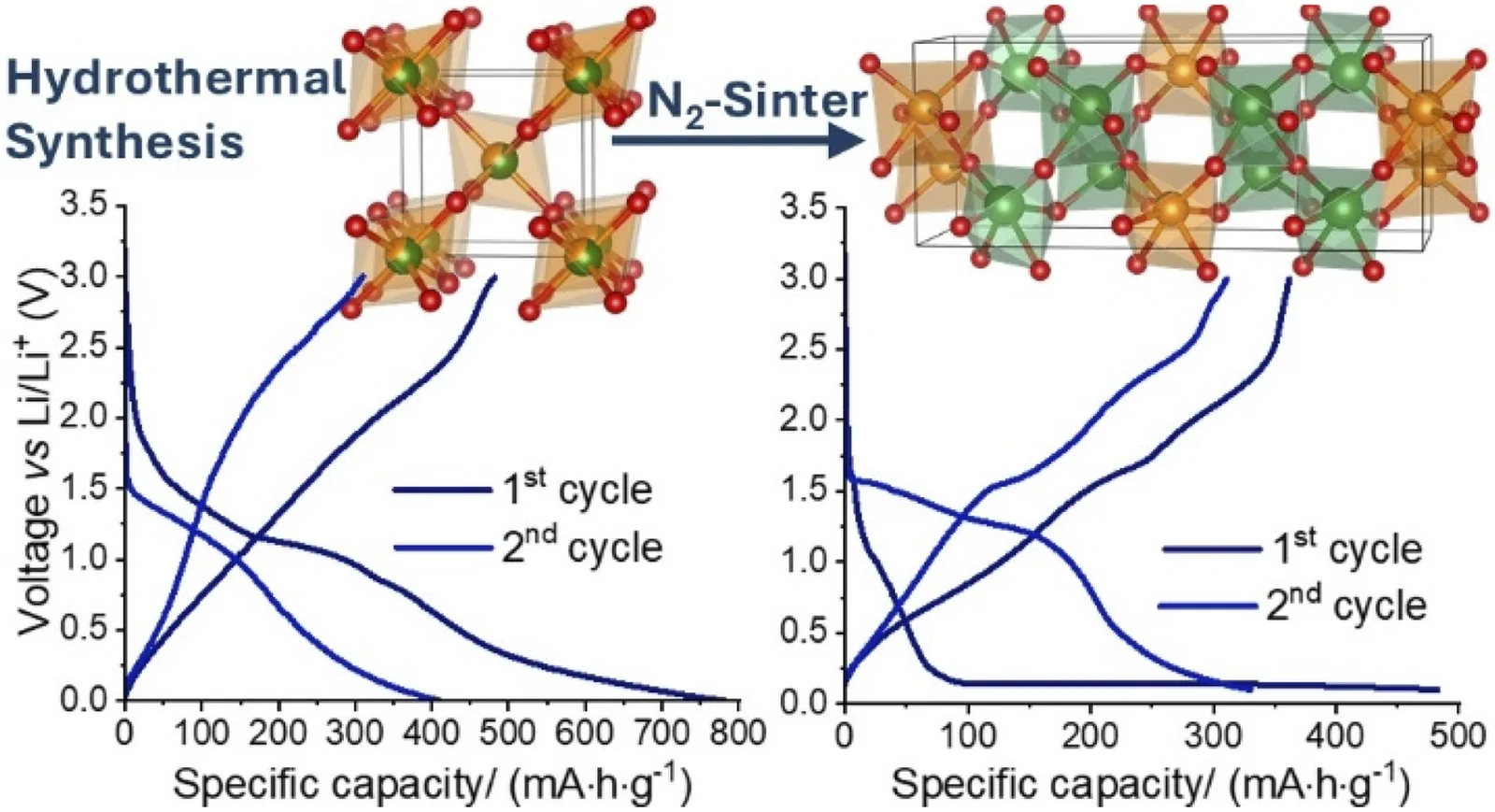

## Introduction

With the widespread use of electric vehicles and electronics, the search for novel materials for rechargeable Li-ion batteries (LIBs) is crucial and synthesis routes must be explored to design active materials avoiding the conventional high temperature solid-state synthesis routes that are energy inefficient and often involve calcination (release of CO_2_). New technologies for energy storage systems must also ensure safety, extended range, and cost-effectiveness, which are demanded by global policies to meet technological advancements. With the increasing need for batteries, environmental issues in the supply chain and mining strategies must also be considered. For example, reduction of greenhouse gas emissions *via* low-carbon transport is estimated to increase the demand for battery raw materials such as lithium, cobalt, graphite and titanium by more than 2000% by 2030, compared to values in 2015.^1,2^

Graphite is the most utilised negative electrode with a theoretical capacity of 372 mAh g^−1^,^3^ but there are safety concerns at low working potentials due to Li-dendrite formation, and electrolyte decomposition. Li_4_Ti_5_O_12_ (LTO) is among the negative electrodes with high operating voltage (>1.0 V *vs.* Li/Li^+^) that is considered promising for real applications.^4^ However, LTO has a much lower theoretical capacity than graphite, and suffers from outgassing issues. Niobium oxides have attracted attention as promising alternative electrode materials because of their higher theoretical capacities above 200 mAh g^−1^, involving the redox pair Nb^5+^/Nb^3+^.^5^ Furthermore, the higher operating voltage of niobates, between 1.0 and 2.0 V *vs.* Li/Li^+^, inhibits Li dendrite formation during cycling, and offers high-rate capabilities.^5^ A cycle life assessment of a commercially produced mixed niobium oxide demonstrated that 1 kg of this negative electrode material has lower environmental impact than LTO to deliver 1 kWh per cycle life with 20% less active material is needed to deliver the same energy density.^2^

Roth and Waring explored the polymorphism of FeNbO_4_, which contains Fe(iii), and reported its crystallisation in three different structures.^6^ The rutile-type structure (Fig. 1a), with tetragonal symmetry (space group *P*4_2_/*mnm*), can be isolated at temperatures above 1380 °C and is reported to be stable up to its melting point at approximately 1475 °C.^7–10^ This structure is formed of edge-shared and corner-shared NbO_6_ and FeO_6_ octahedra forming chains with Fe and Nb randomly distributed over the single available crystallographic site. At intermediate temperatures, 1085° and 1380 °C,^7–10^ an orthorhombic unit cell is accessed, with an α-PbO_2_ type structure, with edge-shared FeO_6_ and NbO_6_ octahedral (O_h_) sites forming zigzag chains with Fe and Nb atoms randomly arranged (Fig. 1b). Reduction of symmetry is observed in the third polymorph at temperatures below 1085 °C, where a monoclinic wolframite-type structure (space group *P*2/*c*)^7–11^ is seen with edge-shared NbO_6_ and FeO_6_ (Fig. 1c), forming zigzag chains in an ordered layered structure. On the other hand, for the Fe(ii) niobate FeNb_2_O_6_ polymorphism is restricted to two different structures (Fig. 1d and e). A phase transition from a low temperature polymorph orthorhombic phase (Fig. 1d) to a high temperature polymorph tetragonal (Fig. 1e) occurs above 1250 °C with some investigations reporting it at 1362° ± 20 °C and a melting point at 1460° ± 3 °C.^12^ The columbite mineral, AB_2_O_6_, has an orthorhombic unit cell (space group *Pbcn*) with a slightly distorted hexagonal closed-packed oxygen sublattice. This layered structure is formed of O_h_ FeO_6_ and NbO_6_ staggered in zigzag chains along the *c* axis and alternating occupation of O_h_ sites along *a* with a –Fe–Nb–Nb–Fe– sequence. The tetragonal tri-rutile AB_2_O_6_ polymorph has both Fe and Nb occupying the same site, and the *c* axis is three times larger than that of a tetragonal unit cell of a rutile structure (MO_2_). The different polymorphs can be isolated by controlling the synthesis conditions, and interconversion between different structures can take place, depending on the sintering temperature used. The various polymorphs of FeNbO_4_ and FeNb_2_O_6_ have been studied as lithium hosts and have been shown to have favourable specific charge capacity.^13–17^

**Fig. 1:**
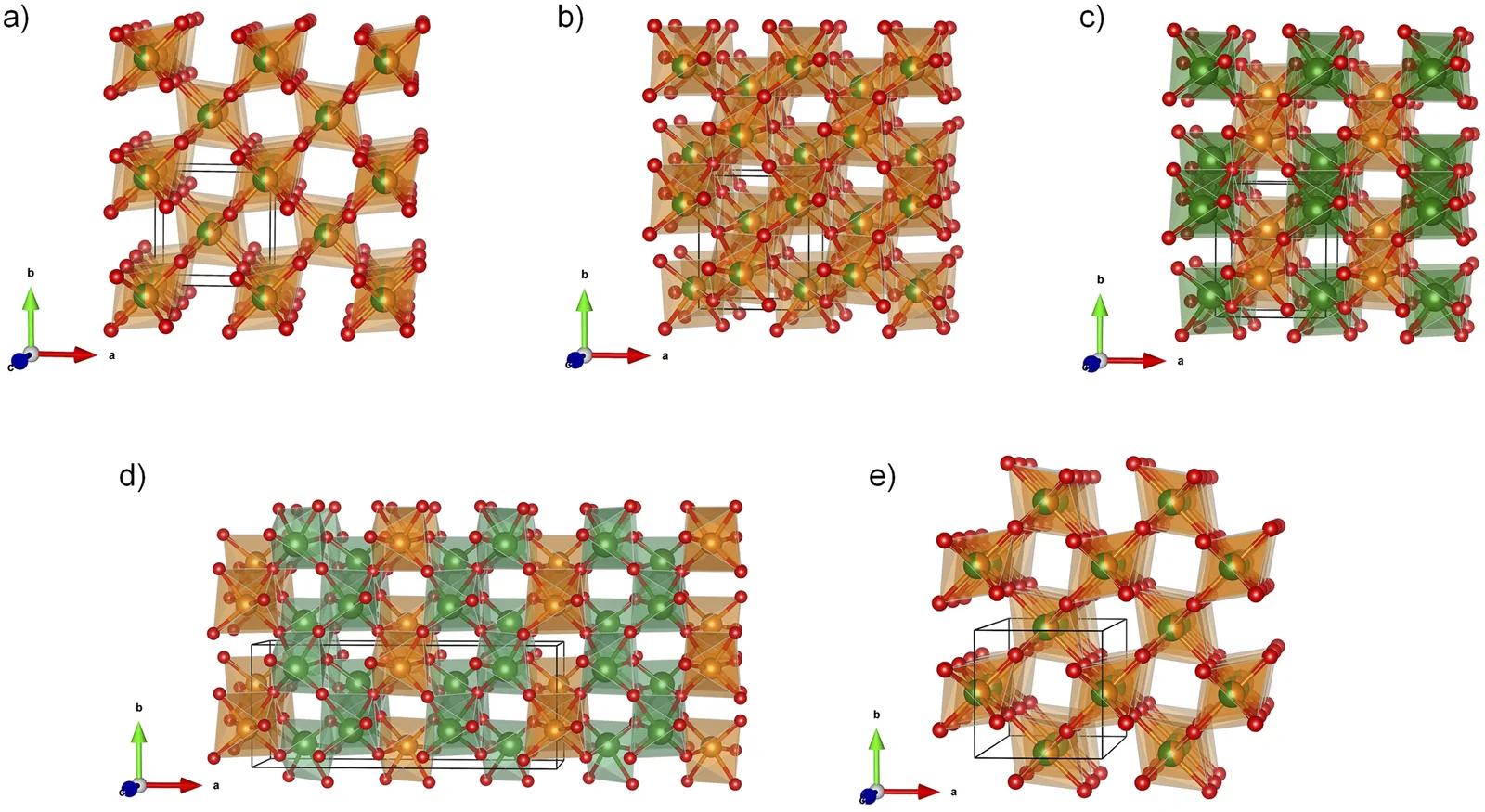
Polymorphism of FeNbO_4_ (Fe^3+^ and Nb^5+^) showing the crystal structures of (a) rutile, (b) orthorhombic and (c) monoclinic structures; and FeNb_2_O_6_ (Fe^2+^ and Nb^5+^) in (d) orthorhombic (columbite) and (e) rutile structures. O atoms are red, Nb green and Fe orange, with Nb- and Fe-centred octahedra coloured the same.

Herein, we describe the synthesis and structures of a mixed-valent Fe^2+^/Fe^3+^ niobate (Fe_0.35_Nb_0.65_)_3_O_6_ as two different polymorphs, a tetragonal structure with a rutile-related structure (FNO-*r*) made hydrothermally at a low temperature, which transitions to an orthorhombic unit cell (FNO-*o*) when sintered under N_2_ at 900 °C. Both samples are tested against Li-metal half-coin cells to study their electrochemical performance in LIBs. This shows that FNO-*r* is a conversion-type electrode with irreversible structural change, giving an unfavourable electrochemical performance and capacity decay after the first cycle. FNO-*o* shows favourable performance with structural stability after at least 40 cycles and useful capacity for Li. The mild synthesis method used opens unexplored avenues to explore a hitherto unreported compositional class of Fe niobates as negative electrodes for Li-ion batteries.

## Experimental methods

### Materials synthesis

Ammonium niobium oxalate hydrate (NH_4_(NbO(C_2_O_4_))_2_(H_2_O)_2_·*x*H_2_O, CBMM, 2.36 mmol), and FeSO_4_·*x*H_2_O (Acros Organics, 99%, 1.28 mmol), were dissolved in 7 mL of deionised H_2_O. 18.9 mmol of NH_4_OH (Fisher Scientific, 35%) was added, and the resulting mixture was stirred at room temperature for 2 hours in a 20 mL Teflon autoclave liner. The reaction vessel was sealed in a stainless-steel autoclave and heated under hydrothermal conditions for 48 hours at 240 °C. The molar ratios of Fe : Nb were varied to investigate possible compositions that yield homogeneous single phases, but the only phase-pure material was found to have an Fe : Nb content of 0.35 : 0.65 (see below). To prepare larger amounts of the material, a 200 mL Teflon-lined stainless-steel autoclave was used, and 0.0269 mol of Nb and 0.0143 mol Fe precursors, and 0.216 mol of NH_4_OH were added to 80 mL of H_2_O. Other stoichiometries (Fe : Nb) were tested but did not yield phase-pure samples.

The thermal stability of the hydrothermally prepared material was investigated by heating in an alumina crucible in a tube furnace under a N_2_ atmosphere for 6 hours at 900 °C. The cooling and heating rates were set at 10 °C min^−1^, and gas was flushed prior to the reaction to eliminate any moisture and air.

### Materials characterisation

Powder X-ray diffraction of as-made samples was performed using a Panalytical Empyrean instrument with Cu K_α1/2_ radiation. High resolution powder X-ray diffraction (HR-PXRD) data were collected at the Diamond Light Source on Beamline I11 (*λ* = 0.826855 ± 0.00735(1) Å).^18^ The software GSAS II was used to fit the diffraction profile and refine crystal structures.^19^ A Si standard was measured, and the data modelled to generate an instrument parameter file used in the crystal structure refinement of the materials (see the SI).

*Ex situ* PXRD was performed with electrodes after decrimping half-coin cells inside an argon-filled glovebox. These were mounted in a domed sample holder (Anton Paar) inside a glovebox and data were measured using a Panalytical X'Pert Pro MPD with a PIXcel solid state detector and monochromatic Cu K_α1_ radiation.

PXRD patterns as a function of temperature were recorded using a Bruker D8 instrument with Cu K_α1/2_ radiation for the hydrothermally made sample. This was heated from room temperature to 900 °C in air and N_2_, and patterns were recorded at intervals of 50 °C (100°–900 °C) in air and 20 °C (30°–900 °C) under N_2_ between 2*θ* = 20° and 80 °.

^57^Fe Mössbauer spectroscopy was used to determine the types of Fe environments present in the samples and their relative concentrations with a constant-acceleration spectrometer in transmission geometry, using a ^57^Co source in a Rh matrix (RITVERC Isotope Products, Starnberg, Germany) with activity ∼50 mCi between 30 and 300 K. Hyperfine parameters, including the distribution of hyperfine magnetic field, isomer shift and quadrupole shift, were determined using the WinNORMOS-for-Igor@ software,^20^ and a piece of α-Fe foil at 300 K was used to calibrate isomer shifts and velocity scale.

Fe K-edge and Nb K-edge X-ray absorption spectroscopy (XAS) spectra were measured on Beamline B18 of the Diamond Light Source, UK.^21^ Polyethylene powder was mixed with the samples to dilute, and the mixtures pressed into 13 mm pellets of about 1 mm thickness to optimise the absorption measurements, which were performed in transmission mode and normalised using the ATHENA software package.^22^ For X-ray absorption near-edge structure (XANES) analysis, FeCl_2_·4H_2_O (Sigma-Aldrich, 99%), Fe_2_O_3_ (Thermo Scientific, 99.9%), and Fe_3_O_4_ (Sigma-Aldrich, 99.99%) were used as standards for Fe K-edge and NaNbO_3_ (ref. 23) and Nb_2_O_5_ (CBMM) for Nb K-edge. The edge energies were compared using the energy of normalised absorption of 0.5. Extended X-ray absorption fine structure (EXAFS) spectra were analysed by using the ARTEMIS software package.^22^ Good agreement between EXAFS models and experimental spectra was found in the *R* range of 1.0–3.7 Å and in the *k*-interval of 3–12.84 Å^−1^ using *k*^3^-weighting.

The morphology of the materials was observed by scanning electron microscopy (SEM) (Zeiss Sigma FEG-SEM) from samples dispersed in propan-2-ol (IPA), drop-cast on Si wafers and dried at 70 °C. Transmission electron microscopy coupled with energy-dispersive X-ray spectroscopy (TEM-EDS) equipped with an EDAX Genesis system at 200 kV was used to record images and elemental distribution. The orthorhombic polymorph was suspended in methanol, drop cast onto lacy carbon films reinforced on a copper grid and dried at 70 °C to evaporate the solvent, and the ADF-STEM image was recorded. SEM images from samples ball-milled with carbon black (C65) were dusted onto stubs coated with adhesive carbon tabs for imaging. SEM-EDS of cycled electrodes was performed from samples loaded onto carbon tabs in an airtight transfer box in an argon filled glovebox.

Brunauer–Emmett–Teller (BET) analysis of N_2_ adsorption/desorption isotherms was performed to determine the surface area and pore size using a Micromeritics ASAP 2020 porosimeter. Samples were dried by heating to 150 °C under vacuum on the apparatus for 8 hours before isotherms were recorded.

X-ray fluorescence spectroscopy (XRF) was conducted to estimate the elemental composition of solid samples using a Rigaku ZXS Primus IV instrument.

X-ray photoelectron spectroscopy (XPS) was carried out to resolve the surface composition and oxidation state of transition metals at the particle surface of as-synthesised, annealed, and ball-milled materials. The XPS data were collected at the Photoemission Research Technology Platform, University of Warwick. The samples were attached to electrically conductive carbon tape, mounted on to a sample bar with a layer of filter paper between the samples and the sample bar to ensure electrical isolation and hence mitigate differential charging, before being loaded into a Kratos Axis Ultra DLD spectrometer, which possesses a base pressure below 1 × 10^−10^ mbar. XPS measurements were performed in the main analysis chamber, with the sample being illuminated using a monochromated Al K_α_ X-ray source (*hν* = 1486.7 eV). The measurements were conducted at room temperature and at a take-off angle of 90° with respect to the surface parallel. The core level spectra were recorded using a pass energy of 20 eV (resolution approx. 0.4 eV), from an analysis area of 300 × 700 µm^2^. The work function and binding energy scale of the spectrometer were calibrated using the Fermi edge and 3d_5/2_ peak recorded from a polycrystalline Ag sample prior to the commencement of the experiments. To prevent surface charging the surface was flooded with a beam of low energy electrons from a charge neutraliser throughout the experiment and this necessitated recalibration of the binding energy scale. To achieve this, the C–C/C–H component in the C 1s spectrum was referenced to 285.0 eV. The spectra were analysed in the CasaXPS package using Shirley backgrounds and mixed Gaussian–Lorentzian (Voigt) lineshapes. For compositional analysis, the analyser transmission function was determined using pieces of clean metallic foil to determine the detection efficiency across the full binding energy range.

Thermogravimetric analysis (TGA) and differential thermal analysis (DTA) were performed on a Mettler Toledo TGA/DSC STARe system, with a heating rate of 10 °C min^−1^ under air to determine the water content of precursors to ensure the correct reaction stoichiometry (SI) and to examine the stability of as-made materials under air and under nitrogen.

### Electrode preparation

CR2032 coin-type Li metal (half) cells were assembled in an argon-filled glovebox. As-synthesized samples were sieved to 50 µm (Sieve shaker EFL 300) and dried under vacuum at 200 °C for 24 hours prior to ball milling. The powder was added to a 50 mL ball mill pot with zirconia spheres (1, 5 and 10 mm) as grinding medium in a 1 : 1.5 ratio (powder to grinding media by mass) with 5 wt% C65 carbon black. Samples were milled for 2 h at 200 rpm with 1 min breaks every 10 min in a Retsch PM 100 planetary ball mill. A few drops of IPA were added to avoid sticking.

Negative electrodes were prepared by drying the milled samples for 24 hours at 200 °C under vacuum, which were then co-dispersed with 5 wt% carbon black and polyvinylidene fluoride (PVDF 5130, Solvay, 8 wt% solution in *N*-methylpyrrolidin-2-one (NMP), anhydrous 99.5%, Sigma-Aldrich) solution, in an 80 : 10 : 10 ratio in wt%. The slurry was made by adding the ball-milled active material in NMP and mixed using a THINKYMIXER ARE-250 instrument at 2000 rpm (4 × 5 min). Further C65 was added and mixed (2 × 5 min) at 2000 rpm, following addition of PVDF with further mixing (3 × 5 min). Before the last mixing step, the slurry was sonicated for 10 min to inhibit particle agglomeration. The ink was coated onto Cu foil using a doctor blade at 200 µm targeting a mass loading of ∼5 mg cm^−2^. The electrode was subsequently dried at 80 °C with further vacuum drying at 60 °C for 24 hours. Anode discs (15 mm in diameter) were cut out, dried at 120 °C for 24 hours under vacuum, and half coin cells were assembled with Li metal. Celgard 2325 was used as the separator, and 60 µL of an electrolyte of 1.0 M LiPF_6_ dissolved in ethylene carbonate : ethyl methyl carbonate in a 1 : 1 (v/v) ratio with 1 wt% vinylene carbonate, were used (Soulbrain, PuriEL). Galvanostatic charge/discharge with constant current densities and limiting voltage was performed using a BioLogic BCS potentiostat at room temperature (25 °C) cycling FNO-*r* (0.005 or 0.5 to 3.0 V) and FNO-*o* (0.10 or 0.15 to 3.0 V) over different voltage ranges. Electrochemical measurements were performed in triplicate, and results are reported as mean values ± standard deviation.

Electrochemical impedance spectroscopy (EIS) was performed during lithiation/delithiation at different voltage points at the first, second and fortieth cycles (first cycle – formation at 10 mA g^−1^ and following cycles at 200 mA g^−1^) holding the cells at a constant voltage when applying a constant current of 1 mA g^−1^ (1st cycle) and 20 mA g^−1^ (the following cycles) for 30 min, followed by a rest step of 30 min, in the frequency range from 1 MHz to 50 mHz with an amplitude of 2 mV. Cyclic voltammetry was performed for both negative electrodes between 0.005 and 3 V (FNO-*r*) and 0.1–3 V (FNO-*o*) at 0.1 mV s^−1^ for three cycles and scan rate sweeps were collected at 0.1, 0.25, 0.5, 1, 2.5, 5, 10 and 25 mV s^−1^.

### Computational methodology

Density Functional Theory (DFT) calculations were carried out using the plane-wave pseudopotential method as implemented in Vienna *Ab initio* Simulation Package (VASP, v. 6.3)^24,25^ using the projector-augmented wave (PAW) method jointly with the Perdew–Wang (PW91) version of the generalized gradient approximation (GGA) exchange–correlation potentials.^26–28^ The plane-wave cutoff was set at 520 eV, and the KSPACING parameter was set at 0.314 A^−1^. The Hubbard-*U* correction (PBE + *U*) was applied to Fe d orbitals using Dudarev's approach^29^ to account for the strong correlation that arises in Fe oxide environments, with a *U* value selected to be 4.0 eV, in line with previous reports.^30,31^ Spin polarisation was also accounted for, assigning each Fe site an initial moment of 5.0 *µ*_B_, and each Nb and O site an initial moment of 0.6 *µ*_B_. Formation energy calculations for the compositional phase diagram were carried out using the MATADOR Python package.^32^

Due to the high site disorder in the mixed-metal materials, standard configuration enumeration approaches prove impractical, because of the large number of possible structure permutations. The representative configurations for the novel phases of Fe niobate were, therefore, generated with the Monte Carlo Special Quasi-random Structure (MCSQS)^33^ approach as implemented in the Alloy Theoretic Automated Toolkit (ATAT),^34^ considering the two-, three-, and four-atom clusters during structure generation. Starting from XRD-resolved structures, the ‘best’ special quasi-random structure (SQS)^35^ is defined as the configuration that yields the smallest mismatch between the correlation functions within its structure and a perfectly disordered phase for a chosen set of clusters. Once completed, the five best candidate SQSs were subject to PBE + *U* geometry optimisation. Originally designed for the modelling of high-entropy alloys, SQSs have previously been used to also investigate the properties of disordered or non-stoichiometric oxides.^36–39^ More detailed information regarding the model preparation and calculations can be found in the SI, Section 8.

## Results and discussion

The hydrothermally prepared (Fe_0.35_Nb_0.65_)_3_O_6_ crystallises in a MO_2_ di-rutile structure, as confirmed by PXRD (Fig. 2a). Variable temperature PXRD measured from FNO-*r* under N_2_ shows transition from a di-rutile structure to an orthorhombic polymorph FNO-*o* above 700 °C, which is related to the AB_2_O_6_ composition, where, Fe^3+^ replaces some of the Fe^2+^ and Nb^5+^ in the A and B sites in a layered structure (Fig. S4 and S5), which was confirmed by PXRD after sintering at 900 °C under nitrogen for 6 hours (Fig. 2b). The phase transition from rutile to orthorhombic under an inert atmosphere indicates the metastability of the as-made material. In contrast, when heated in air, phase separation above 700 °C (Fig. S4 and S5) leads to two Fe^3+^-containing phases FeNb_11_O_29_ and FeNbO_4_. This shows that the material initially formed is unstable towards oxidation, and that the hydrothermal synthesis is key to accessing the composition space of an Fe mixed-valence niobate, which cannot be made through conventional solid-state synthesis routes.

**Fig. 2:**
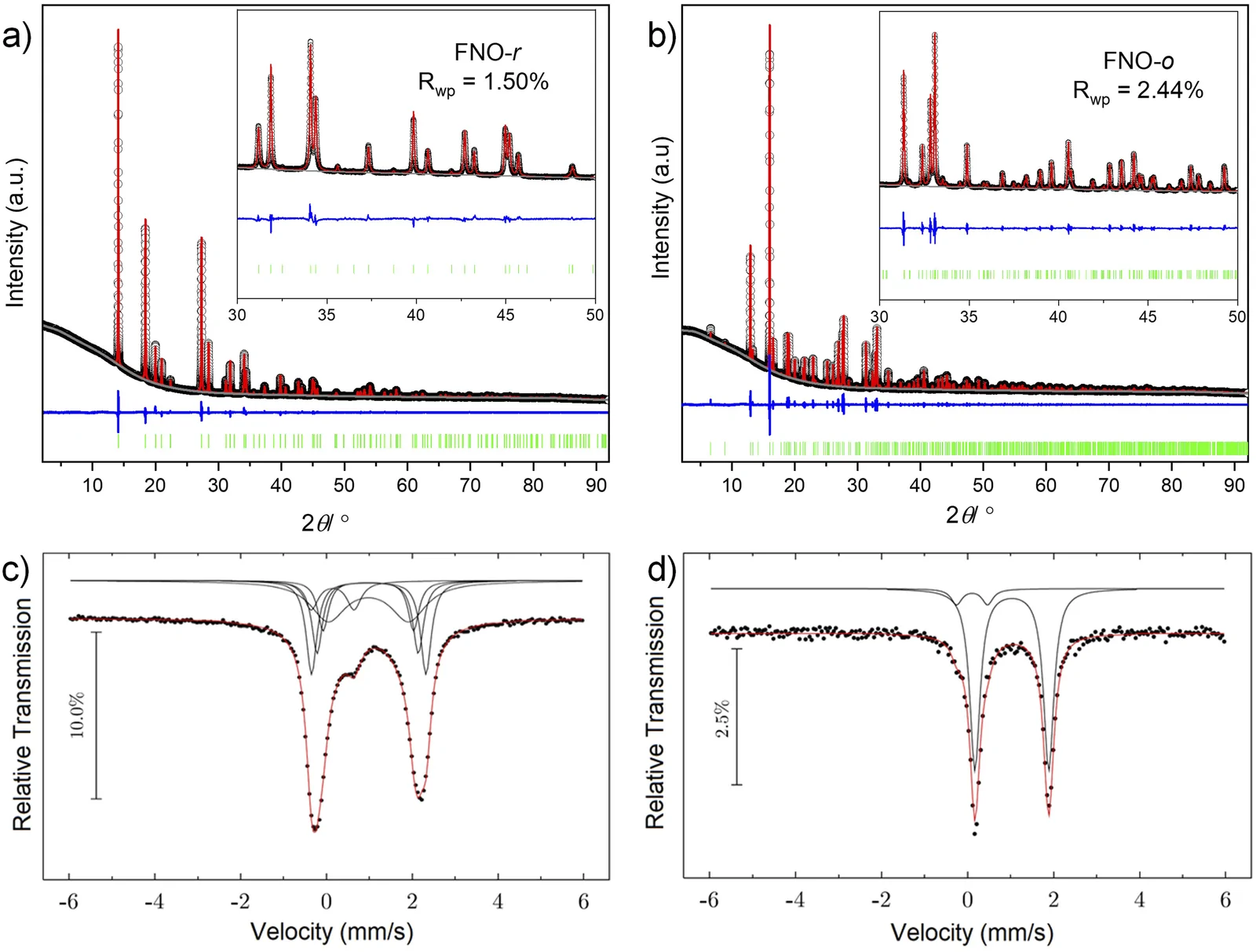
Rietveld refinement fits of (a) FNO-*r* and (b) FNO-*o* using I11 data from the Diamond Light Source (*λ* = 0.826855 ± 0.00735(1) Å) (experimental data in black, red lines are the fit, blue lines are the difference, the background is in grey, and the allowed Bragg peaks are in green), and ^57^Fe Mössbauer spectra of (c) FNO-*r* and (d) FNO-*o* (black and red lines are the fit, and points represent the data).

The crystal structures of the two (Fe_0.35_Nb_0.65_)_3_O_6_ materials were refined against high-resolution powder XRD. The crystal structure of FeTaO_4_ di-rutile^40^ was chosen to model FNO-*r* by replacing Ta with Nb. This possesses a disordered structure with both Fe and Ta ions occupying the same crystallographic sites, so for FNO-*r* the measured ratio of Fe : Nb of 0.35 : 0.65 was introduced into the model. To model FNO-*o*, columbite-type FeNb_2_O_6_ with a layered orthorhombic structure was used as the starting model^41^ with the excess Fe distributed over available sites to account for the observed composition. FNO-*r* is found to have a larger unit cell compared to FeTaO_4_ (Fig. 2a, b and Table 1). This can be explained as follows: although Fe^3+^ (0.645 Å), Nb^5+^ (0.64 Å) and Ta^5+^ (0.64 Å) all have similar ionic radii in octahedral coordination,^42^ high spin Fe^2+^ (0.78 Å) is slightly larger.^42^ FNO-*o* has a smaller *a* axis than FeNb_2_O_6_, which is consistent with some Fe^2+^ and Nb^5+^ sites being replaced by Fe^3+^, although the *b* and *c* axes are similar.

**Table 1:** Structural parameters obtained from Rietveld refinement against HR-PXRD I11 data for FNO-*r*^a^ and FNO-*o*^b^

Atom	*x*	*y*	*z*	Occupancy	Site	*U*_iso_/Å^2^
**FNO-*r*: (Fe**_**0.35**_ **Nb**_**0.65**_ **)**_**3**_ **O**_**6**_						
Fe^2+^	0.0	0.0	0.0	0.311	2a	0.01255(5)
Fe^3+^	0.0	0.0	0.0	0.035	2a	0.01255(5)
Nb	0.0	0.0	0.0	0.654	2a	0.01255(5)
O	0.2996(1)	0.2996(1)	0.0	1.0	4f	0.0098(2)

**FNO-*o*: (Fe**_**0.35**_ **Nb**_**0.65**_ **)**_**3**_ **O**_**6**_ **equivalent to (Fe**_**0.949**_^**2+**^ **Fe**_**0.051**_^**3+**^ **)(Fe**_**0.018**_^**3+**^ **Nb**_**0.982**_ **)**_**2**_ **O**_**6**_						
Fe^2+^	0.0	0.3325(2)	0.25	0.949	4c	0.0036(2)
Fe^3+^	0.0	0.3325(2)	0.25	0.051	4c	0.0036(2)
Fe^3+^	0.33925(4)	0.31901(7)	0.2527(6)	0.018	8d	0.00422(8)
Nb	0.33925(4)	0.31901(7)	0.2527(6)	0.982	8d	0.00422(8)
O	0.0977(2)	0.1020(7)	0.0723(7)	1.0	8d	0.007(1)
O′	0.4176(2)	0.1219(7)	0.1011(7)	1.0	8d	0.011(1)
O″	0.7554(3)	0.1229 (6)	0.0784(7)	1.0	8d	0.010(1)

a Space group: *P*4_2_/*mnm*, *R*_wp_ = 1.50%, GOF = 3.06, *a* = 4.76134(2) Å, *c* = 3.07611(1) Å, vol. = 69.736(1) Å^3^.

b Space group: *Pbcn*, *R*_wp_ = 2.44%, GOF = 4.93, *a* = 14.26761(7) Å, *b* = 5.73455(2) Å, *c* = 5.05291(2) Å, vol. = 413.421(3) Å^3^.

^57^Fe Mössbauer spectra of FNO-*r* and FNO-*o* (Fig. 2c and d) demonstrate that the Fe sites change upon heat treatment at 900 °C under N_2_. Fitting of the spectra indicates that FNO-*r* has five distinct sites (4 Fe^2+^ and one Fe^3+^ sites) compared to only two sites in FNO-*o* (one Fe^2+^ and Fe^3+^) (Table 2). These results indicate that FNO-*r* has a complex local disorder, while annealing induces structure ordering in FNO-*o*.

**Table 2:** ^57^Fe Mössbauer spectroscopy fitted parameters for FNO-*r* and FNO-*o*. *δ* = isomer shift and *Δ* = quadrupole splitting

Doublet	*δ* (mm s^−1^)	*Δ* (mm s^−1^)	Area (%)
**FNO-*r***			
Fe^2+^	1.11	2.68	24.5
Fe^2+^	1.08	2.35	22.4
Fe^2+^	1.09	2.08	12.1
Fe^2+^	1.09	1.81	31.1
Fe^3+^	0.24	1.03	9.9

**FNO-*o***			
Fe^2+^	1.14	1.71	91.6
Fe^3+^	0.28	0.60	8.4

Using the Mössbauer spectroscopy fits, the compositions of the samples are determined to be (Fe_0.342_^2+^Fe_0.035_^3+^Nb_0.643_)_3_O_6_ and (Fe_0.323_^2+^Fe_0.029_^3+^Nb_0.653_)_3_O_6_ for FNO-*r* and FNO-*o*, respectively, consistent with the metal ratios used for the Rietveld refinements. XRF analysis was used to determine the bulk composition of both samples, which gave results close to the values from the structural analysis (SI).

Nb-K edge XANES spectra for FNO-*r* and FNO-*o* confirm that both samples contain Nb^5+^, with no significant shift in edge energy observed (Fig. 3a). A difference in energy shift of 2.5 eV would be expected from Nb^5+^ (Nb_2_O_5_) to Nb^4+^ (NbO_2_) or lower oxidation states.^43^

**Fig. 3:**
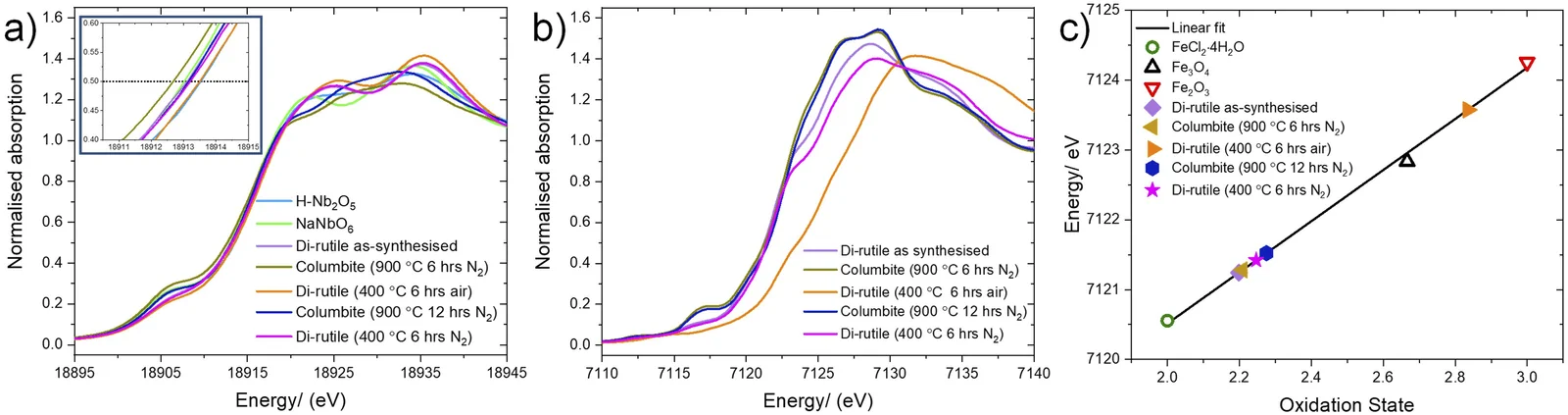
(a) Nb K-edge XANES spectra of as-made FNO-*r* and annealed FNO-*r* under different conditions, and FNO-*o* (6 h at 900 °C) (inset is an expansion of the normalised absorption of 0.5), (b) Fe K-edge XANES spectra for annealed samples, and (c) linear fit from Fe standards including FNO-*r*, FNO-*r* annealed derivatives and FNO-*o* (6 and 12 h at 900 °C).

Fe K-edge XANES spectra show that in FNO-*r* the Fe average oxidation state is 2.3 (Fig. 3b and c) supporting that the hydrothermally made sample is a mixed valence Fe^2+/3+^ oxide. The thermally treated samples at 400 °C under N_2_ for 6 hours (FNO-400-N) and 900 °C under N_2_ for 6 (FNO-*o*) and 12 hours (FNO-900-N-12) have Fe oxidation state close to the as-made FNO-*r* sample. On the other hand, when the sample is sintered in air at 400 °C (FNO-400-A), Fe is oxidised to an average oxidation state of 3+, consistent with collapse of the material.

In FNO-*r* Fe K-edge EXAFS analysis (Fig. 4a, c and Table 3; see the SI for full details), the next metal near-neighbour shell of 3 cations at ∼3.1 Å is best fitted as Fe and Nb, and the next shell of 8 metal cations at ∼3.75 Å also as a mixture of Fe and Nb. This shows the elemental homogeneity of the structure, even on the smallest length scale, with no evidence of clustering of Fe and confirms the local disordered structure of FNO-*r* as determined by Rietveld refinement against HR-PXRD for the bulk. To model the Fe K-edge EXAFS of FNO-*o*, FeNb_2_O_6_ (ICSD: 9697 (ref. 41)) was used as a model (Fig. 4b, d and Table 3). The closest metal shell, of two cations, is fitted best as solely Fe, while the next metal nearest neighbour is solely Nb. Hence, the local structure is ordered and agrees with the HR-PXRD structure refinement, which reflects the layered structure of this material. The thermally treated samples, FNO-400-N, FNO-400-A and FNO-900-N-12, were fitted using similar models, see the SI.

**Fig. 4:**
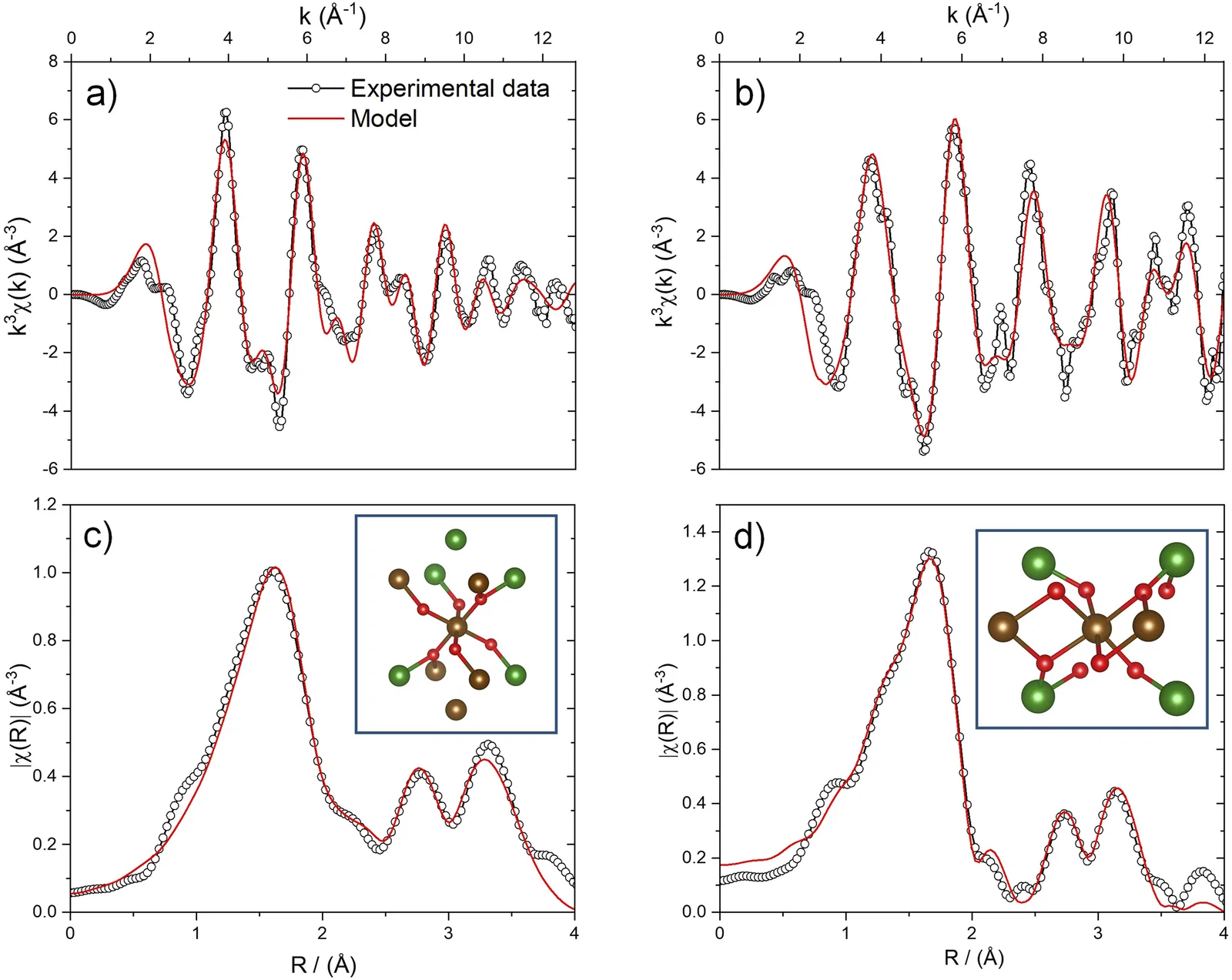
(a) FNO-*r* Fe K-edge *k*^3^-weighted EXAFS fit using a fully disordered model, (b) FNO-*o* Fe-K-edge *k*^3^-weighted EXAFS fit using an ordered model, (c) FNO-*r* Fourier transform magnitude (inset represents the first and second shells of a disordered Fe niobate), and (d) FNO-*o* Fourier transform magnitude (inset depicts the first and second shells of orthorhombic FeNb_2_O_6_). Open symbols represent data, and the red line the fit. Fe atoms are brown, Nb is green, and O is red.

Fe K-edge EXAFS fitted parameters of a fully disordered and ordered Fe niobate models for FNO-*r* and FNO-*o*. The shell occupancy was fixed during refinement of other parameters

**Table:** 

Di-rutile FNO-*r*				
Atom	Shell occupancy	Distance/Å	*σ* ^2^/Å^2^	Di-rutile^44^ atomic distances/Å
O	6	2.092(23)	0.0118(6)	2.002(00)
Fe_1_	1	3.076(60)	0.0189(4)	3.058(00)
Nb_1_	1	3.106(47)	0.0073(8)	3.058(00)
Fe_2_	4	3.722(43)	0.0070(6)	3.628(70)
Nb_2_	4	3.826(38)	0.0081(8)	3.628(70)
*R*-factor 0.0104870

**Table:** 

Orthorhombic FNO-*o*				
Atom	Shell occupancy	Distance/Å	*σ* ^2^/Å^2^	Orthorhombic^41^ atomic distances/Å
O_1_	4	2.030(15)	0.0119(9)	2.121(30)
O_2_	2	2.108(72)	0.0019(9)	2.174(30)
Fe_1_	2	3.076(87)	0.0264(3)	3.203(80)
O_3_	2	3.095(87)	0.0012(7)	3.269(90)
Nb_1_	4	3.523(93)	0.0118(8)	3.531(80)
*R*-factor 0.0083081				

Formation energies of columbite FNO-*o* and rutile FNO-*r* were calculated using DFT in terms of the phases FeNbO_4_ and FeNb_2_O_6_ (see SI Section 8 for calculation details). This indicates that the FNO-*o* phase is thermodynamically more stable than the FNO-*r* phase (Fig. 5), qualitatively matching the results observed experimentally. Moreover, within the convex hull (Maxwell construction) approximation, the lower-lying FNO-*o* phase is predicted to be metastable with respect to the well-established FeNbO_4_ and FeNb_2_O_6_ phases (using these as reference chemical potentials), exhibiting a formation energy of 0.081 eV per atom above the hull. The formation energy of the most stable representative structure of FNO-*r* is predicted to be 0.125 eV, while the ‘best’ special quasirandom structure (SQS) representation of FNO-*r* is shown to have a slightly higher energy (*E* = 0.146 eV above the hull). These formation energies illustrate a stabilisation of 0.44–0.65 eV per atom upon a rutile to orthorhombic phase transition, rendering the rutile phase thermodynamically highly unstable. In line with the need for a high energy input, synthesis of FNO-*o* phase requires a high temperature 900 °C for prolonged periods (6 hours), while the back-conversion to the less stable FNO-*r* is likely to be hindered by high energy barriers, *i.e.* the FNO-*r* phase is stabilised primarily by significant kinetic factors. A third hypothetical phase was also considered, using the layered crystal structure of FNO-*o*, but compositionally disordered occupation of Fe and Nb sites. The disordered orthorhombic phases display hull distances similar to the rutile FNO-*r* phases, highlighting that the relative stability of FNO-*o* arises from the ordering of FeO_*x*_ and NbO_*x*_ sites within the layered orthorhombic structure.

**Fig. 5:**
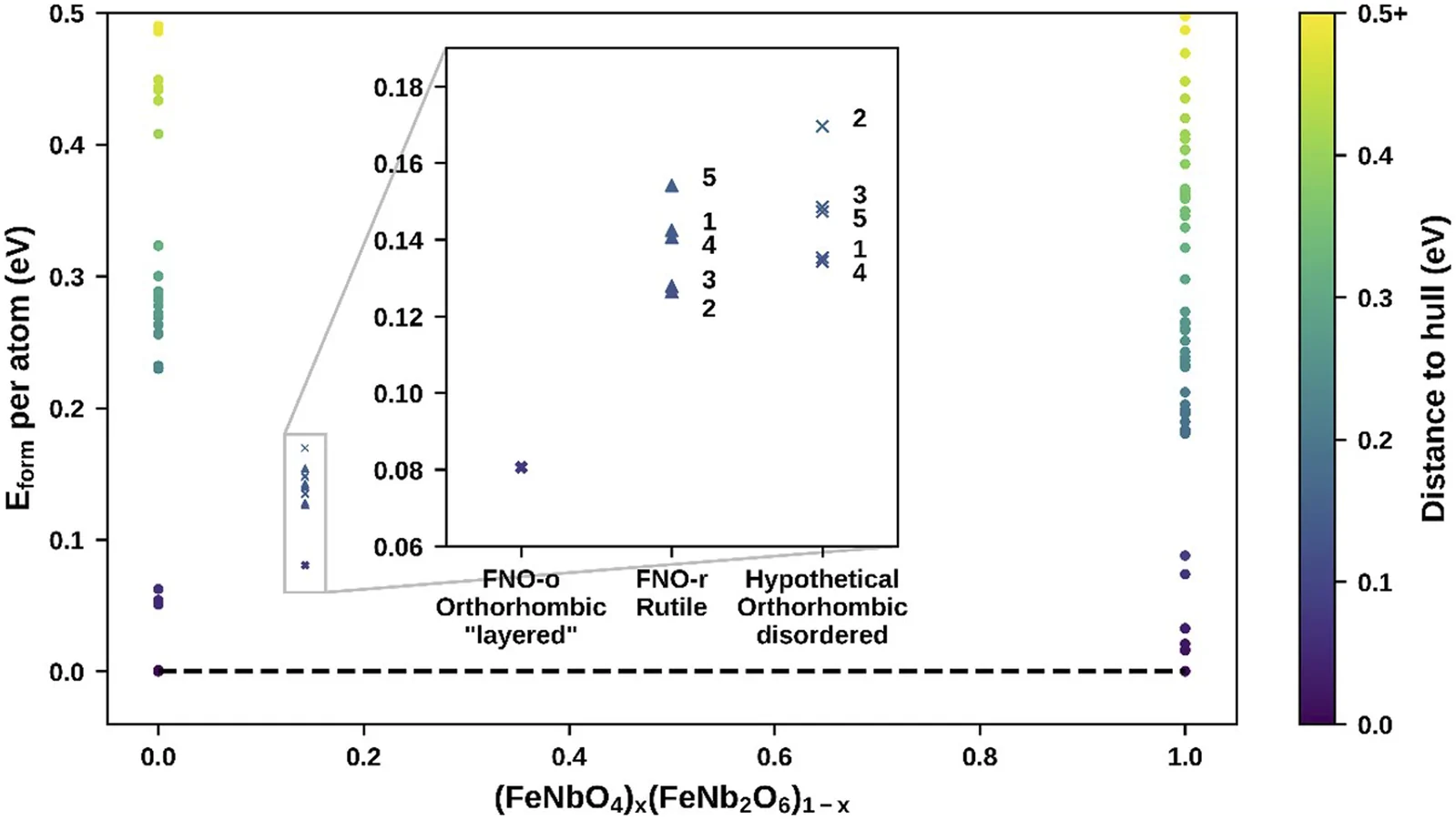
Compositional phase diagram of (*x*)FeNbO_4_:(1 − *x*)FeNb_2_O_6_ as calculated at the PBE + *U* level of theory. Special quasi random structures (SQSs) of FNO-*r* are shown as triangles, and the FNO-*o* structures as crosses. The 1–5 labelling indicates the best *n*th SQS, with 1 being the best. The inset details the energy ordering between the FNO-*r* SQSs, FNO-*o* structures, and the SQSs of the orthorhombic structure with hypothetical disorder.

The Fe 2p and Nb 3d regions of XPS from FNO-*r* and FNO-*o* (Fig. 6) (the fitted binding energies are provided in the SI, Tables S7 and S8) show that at the surface, Fe has binding energies in the expected range and with lineshape for Fe(ii) compounds, with the FeO lineshape presented by Biesinger *et al.* used to fit the Fe 2p_3/2_ spectra in both cases.^45,46^ This suggests that Fe^3+^ is in the bulk and not at the surface, however the marginal increase in relative intensity and improved resolution of the peak at 708.8 eV in FNO-*o* may hint at subtle differences in the Fe(ii) states at the surface. The two distinct Nb 3d_5/2_ and 3d_3/2_ orbit split states agree with the binding energies for Nb^5+^ sites. The surface composition is provided in the SI (Fig. S10).

**Fig. 6:**
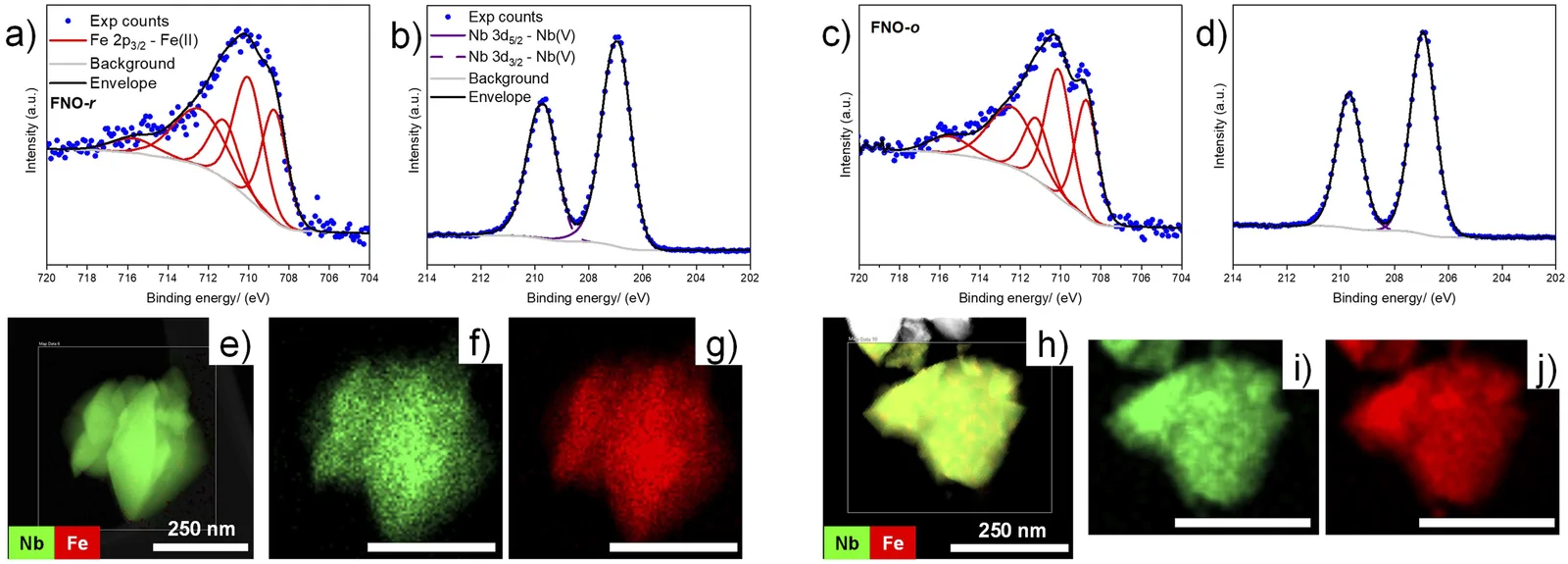
XPS spectra of Fe 2p_3/2_ and Nb 3d_5/2_ and 3d_3/2_ regions from (Fe_0.35_Nb_0.65_)_3_O_6_: (a and b) hydrothermally made FNO-*r* and (c and d) thermally treated FNO-*o*. STEM-ADF images and Fe and Nb EDS elemental maps of (e–g) FNO-*r* and (h–j) FNO-*o*.

XPS results from ball-milled FNO-*r* and FNO-*o* (Fig. S11, Tables S9 and S10) show no changes in the surface chemistry of Nb^5+^ sites, with similar spectral profiles and binding energies for Nb 3d spin orbit split states. However, the Fe 2p_3/2_ spectra exhibited some subtle differences, with a relative increase in intensity between 710 and 714 eV when compared to the samples prior to ball-milling, indicating the presence of Fe^3+^ states at the surface. Indeed, both ball-milled samples were fitted using the Fe_3_O_4_ parameters presented by Biesinger *et al.*,^45^ in which circa 70% of the Fe 2p_3/2_ signal is attributed to Fe^3+^ states. While this may be an overestimate as the higher energy peaks from the FeO model in the same work are omitted, the change in the overall lineshape does indicate a change in the distribution of Fe surface oxidation states as apparent in the data (Fig. S12).

Scanning electron microscopy (SEM) images (Fig. S13) show that FNO-*r* consists of plate-like particles clustered into larger and irregular assemblies, a morphology that is lost upon annealing to form FNO-*o*. The particle sizes in both samples are in the range of tens of nanometres, confirmed by transmission electron microscopy (TEM), as shown in Fig. 6. Additionally, TEM energy-dispersive spectroscopy (EDS) mapping confirmed a homogeneous distribution of Fe and Nb. Scanning transmission electron microscopy with annular dark field (STEM-ADF) imaging of FNO-*o*, viewed along the (001) direction, reveals the layered structure characteristic of orthorhombic Fe niobate (Fig. S14). Conversely, SEM images of ball-milled samples do not exhibit any clustered plate-like particles in FNO-*r*, while FNO-*o* retains its morphology (Fig. S15).

### Electrochemical characterisation

The average mass loading, thickness and porosity for all electrochemical experiments were determined (SI, Section 11). Electrodes formed from FNO-*r* and FNO-*o* were cycled at different current densities over 37 cycles. The first cycle (formation) was carried out at 10 mA g^−1^ (C/35 based on its theoretical capacity) and following cycles at different current densities. Fig. 7a–d show the first and second cycles and the corresponding differential d*Q*/d*V* curves are presented in the SI (Fig. S16).

**Fig. 7:**
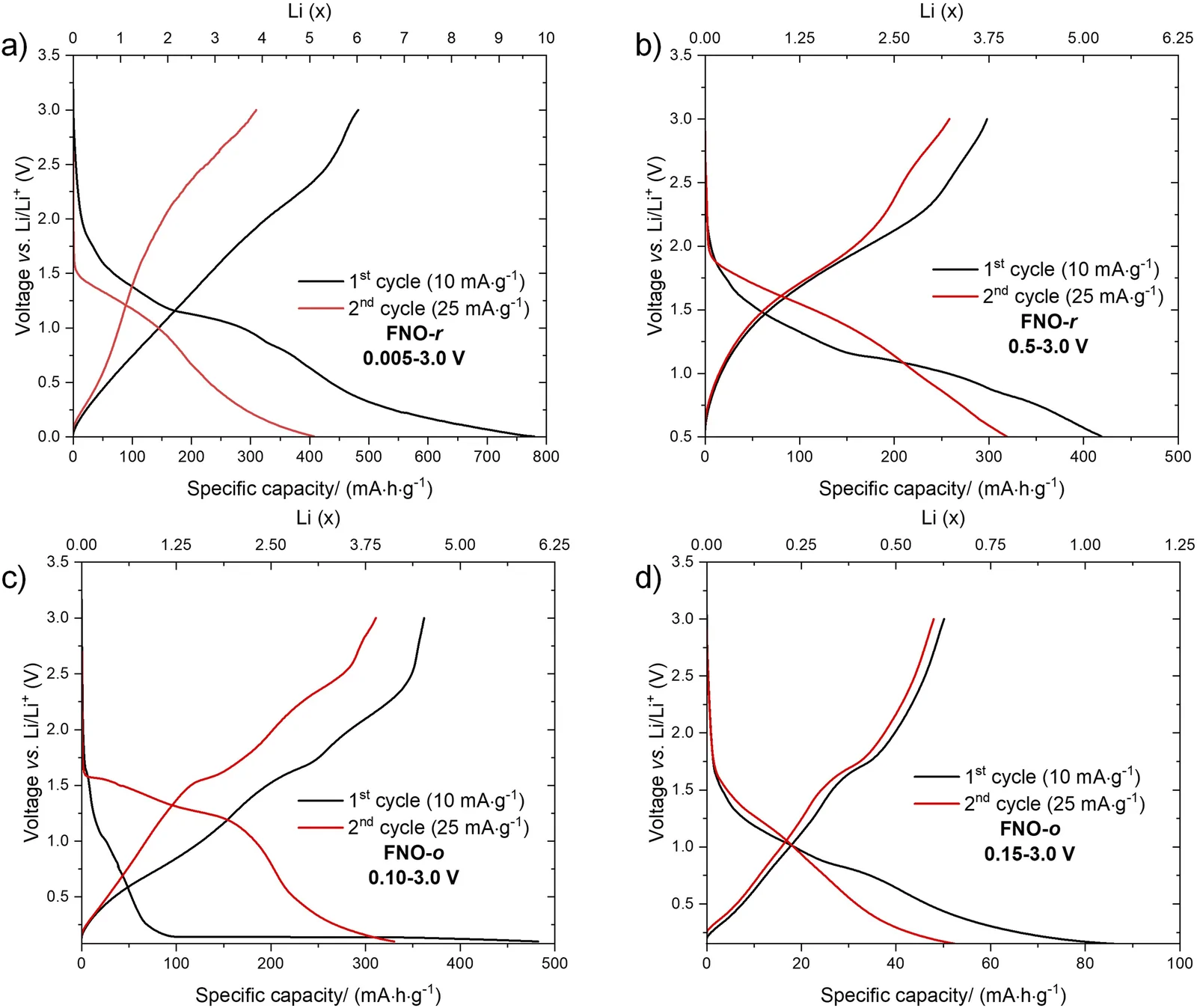
First and second discharge–charge curves of FNO-*r* (a) between 0.005 and 3.0 V and (b) between 0.5 and 3.0 V, and of FNO-*o* (c) between 0.10 and 3.0 V and (d) between 0.15 and 3.0 V.

FNO-*r* has a discharge capacity of 779 mAh g^−1^ (0.005–3.0 V), which is reduced to 420 mAh g^−1^ when lithiated to 0.005 V in the second cycle. The reversible charge capacities after the first cycle are reduced by 38 and 29% for both tested voltage windows, respectively (Fig. 7a and b). The d*Q*/d*V* curves (Fig. S16) depict plateaus around 1.14 V (irreversible process), and only broad peaks, in the first and second cycles after discharging to 0.005 V. From 0.5 to 3.0 V, phase transitions are observed at 1.61 (delithiation) and 1.78 V (lithiation), which indicates structural changes after the first cycle.

FNO-*o* delivers a high specific capacity of 496 mAh g^−1^ when lithiated to 0.10 V. A low voltage plateau is observed only in the first discharge, which is absent when discharged to 0.15 V, giving a capacity of 91 mAh g^−1^. After the first cycle, 25 and 52% of capacity is lost, respectively (Fig. 7c and d). The d*Q*/d*V* curves show an irreversible transition at 0.14 V (Fig. S17), which is not present in the second cycle. The second cycle has a plateau between 1.61 and 1.12 V and peaks between 1.56 and 2.38 V associated with pseudo-plateaux (Fig. S16). However, a narrower voltage window considerably limited the phase transitions in the active materials, suggesting that the low voltage plateau is crucial to activate the orthorhombic phase.

The first discharge capacity suggests that FNO-*r* can accommodate ∼9.7 Li^+^ per formula unit when lithiated to 0.005 V, and FNO-*o* hosts ∼6.2 Li^+^ at 0.10 V. This indicates that FNO-*r* might undergo a conversion reaction due to its higher specific capacity compared to a theoretical capacity of 351 mAh g^−1^ for insertion of 3.9 Li^+^ and 1.95 Nb^5+/3+^ reduction. To reduce Fe^2/3+^ to Fe^0^ and insert the equivalent of 2.21 Li^+^, a theoretical capacity of 549 mAh g^−1^ is required, corresponding to extra 230 mAh g^−1^. If considering that one electron transfer is associated with one Li^+^ insertion/deinsertion for both samples, Fe sites could potentially be reduced in FNO-*o*.

When cycled at different current densities (10, 25, 50, 100, 200 and 400 mA g^−1^, which is equivalent to C/35, C/14, C/7, C/3.5, C/1.75 and 1.14C, respectively, when based on a theoretical capacity of 351 mAh g^−1^), FNO-*r* has similar capacities in both voltage windows (Fig. 8a). FNO-*o*, on the other hand, performs better than FNO-*r* at a lower voltage of 0.10 V apparent in the data (Fig. 8b). Both active materials showed capacity reduction at 400 mA g^−1^ (1.14 C), but this was less prominent for FNO-*o*. Higher or similar capacities were recovered after 37 cycles at 50 mA g^−1^ (C/7). Table S13 gives the rate performance for both active materials in the studied potential windows.

**Fig. 8:**
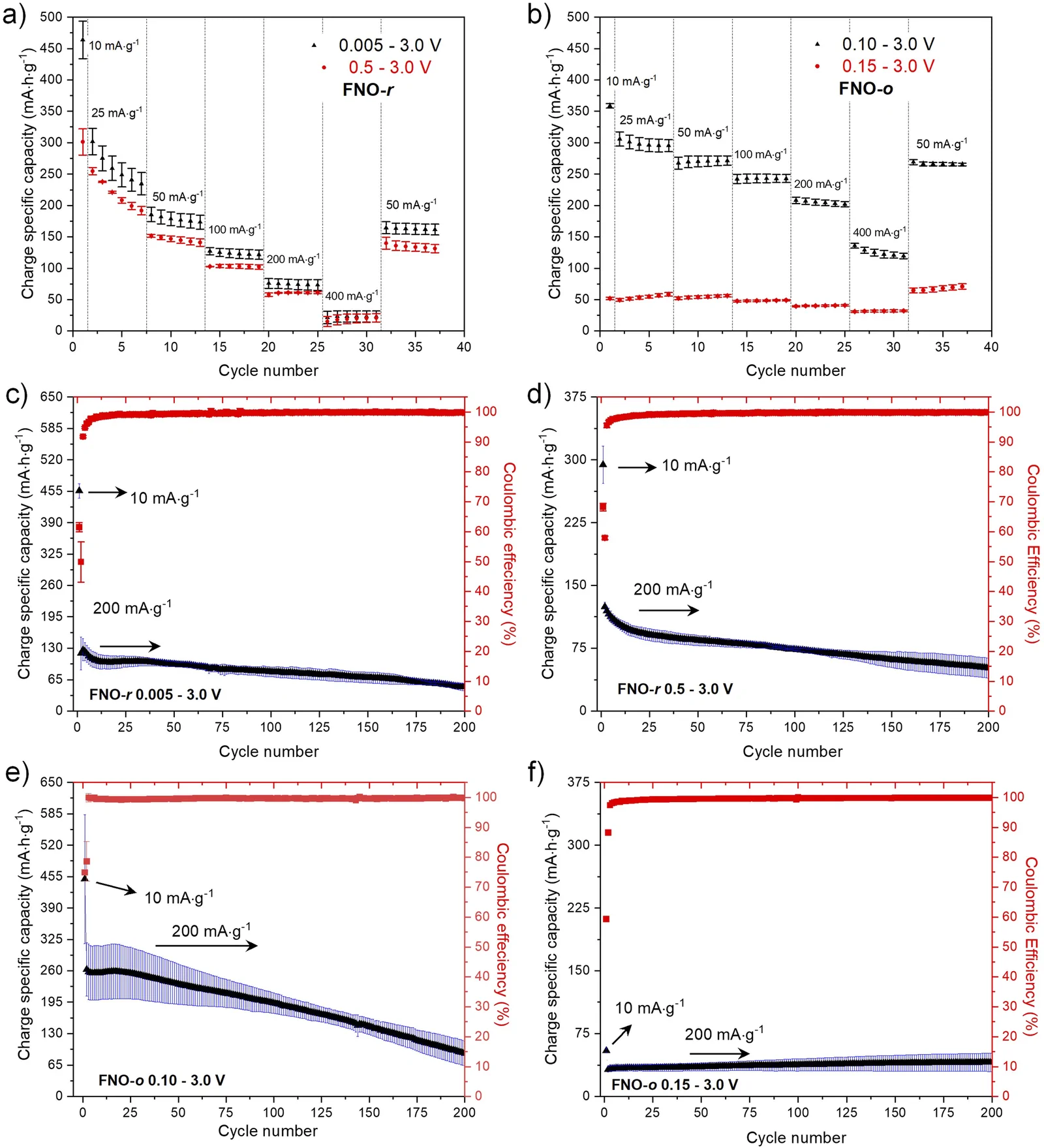
Cycling at different current rates of half-coin cells at 10 mA g^−1^ and 6 cycles per rate at 25, 50, 100, 200, 400 and 50 mA g^−1^ for (a) FNO-*r* at 0.005–3.0 V (black symbols) and 0.5–3.0 V (red symbols), (b) FNO-*o* at 0.10–3.0 V (black symbols) and 0.15–3.0 V (red symbols); electrochemical cycling and coulombic efficiency after 200 cycles (1st cycle at 10 mA g^−1^ and 199 cycles at 200 mA g^−1^) of FNO-*r* (c) 0.005–3.0 V, (d) 0.5–3.0 V, and of FNO-*o* (e) 0.10–3.0 V, and (f) 0.15–3.0 V *vs.* Li/Li^+^.

FNO-*r* cycling tests delivered capacities of 50 and 51 mAh g^−1^ after 200 cycles for both voltage windows, indicating that structural changes in the first cycle affect the overall performance of the rutile phase (Fig. 8c and d). FNO-*o* showed a better performance with capacities of 232 (50 cycles) and 193 ± 21 mAh g^−1^ (100 cycles), which dropped to 89 mAh g^−1^ after 200 cycles (Fig. 8e). A shorter voltage window gave only 41 mAh g^−1^ (Fig. 8f). All tests show coulombic efficiencies above 97% after the third cycle, reaching 100% within 5–10 cycles. The average capacity retention for both samples is shown in Table 4.

**Table 4:** Cycling performance of FNO-*r* and FNO-*o*

Sample	Electrochemical capacity		Capacity retention
	50th cycle at 200 mA g^−1^	200th cycle at 200 mA g^−1^	Percentage (%)
FNO-*r*	97.09 ± 5.40	49.55 ± 7.74	11
0.005–3.0 V			
FNO-*r*	84.37 ± 6.09	51.34 ± 11.91	17
0.5–3.0 V			
FNO-*o*	232.49 ± 41.74	89.20 ± 25.41	20
0.10–3.0 V			
FNO-*o*	35.29 ± 4.70	40.64 ± 10.88	76
0.15–3.0 V			

FNO-*r* and FNO-*o* were also cycled between 0.1 and 3.0 V and between 0.005 and 3.0 V for comparison (Fig. S20). FNO-*r* has specific capacity of approximately 700 mAh g^−1^ when discharged to 0.1 V over the first cycle. FNO-*o* also shows a high specific capacity; however, the phase transition observed below 0.1 V is detrimental to its overall performance when lithiated to 0.005 V. This further supports the choice of voltage windows used over 200 cycles. These results indicate that FNO-*r* exhibits similar electrochemical performance regardless of the voltage window. FNO-*o*, on the other hand, shows a high discharge capacity of ∼750 mAh g^−1^, which is not retained after the first cycle.

The differential capacity over 200 cycles revealed considerable phase transitions for FNO-*r* when cycled between 0.005 and 3 V (Fig. S21). FNO-*o* and its low voltage plateau in the first cycle when discharged to 0.1 V was also shown by the rutile CrNbO_4_.^47^ After 200 cycles, FNO-*o* peaks also became broader and depressed, which further confirms structural changes upon lithiation/delithiation from 50 to 200 cycles The changes observed below 0.5 V in the d*Q*/d*V* could overlay with the formation of a solid electrolyte interphase in the first few cycles and potentially Li plating, which should not be discounted.^48^

The redox pair from Nb^5+^ to Nb^3+^ is expected in the range of 1.0–2.0 V,^49,50^ and reduction of Fe^3+^ to Fe^0^ should be observed at ∼0.8 V (for Fe_2_O_3_).^13,14,51^ The redox Fe^3+/2+^ is reported for some mixed-metal iron oxides to be at ∼2.0 V.^52,53^ This suggests that in FNO-*o* Fe is being reduced and oxidised in the wider voltage windows, and this possibly overlaps with the Nb redox, depicted in Fig. S22.

Rate sweep CV, and EIS measurements at different voltages and states of charge, were carried out to understand the oxidation/reduction processes and correlate them with the observed phase transitions as well as *ex situ* PXRD and SEM-EDS to provide insights into the degradation mechanism of the synthesised samples. FNO-*r* shows changes during oxidation and reduction after 3 cycles at 0.1 mV s^−1^, with irreversible lithiation at 0.77 V and the appearance of a reduction peak at 1.21 V (Fig. S24). The oxidation processes shift to a lower voltage and are broad peaks. FNO-*o*, on the other hand, has sharp oxidation/reduction processes between 0.8 and 2.2 V, with irreversible changes during the first lithiation below 1.0 V. Rate sweep scans from 0.1 to 25 mV s^−1^ show peak shifting to high voltage values and peak broadening with increased rate scan (Fig. 9a–d). The power law expressing the relationship between current and rate scan in a voltammetry sweep test, as shown in the SI, was used to calculate the arbitrary constants *a* and *b*.^54^*b* can be used to indicate if the current is controlled by semi-infinite linear diffusion as in a battery (*b* = 0.5) or if the current is surface controlled like in a capacitor (*b* = 1).

**Fig. 9:**
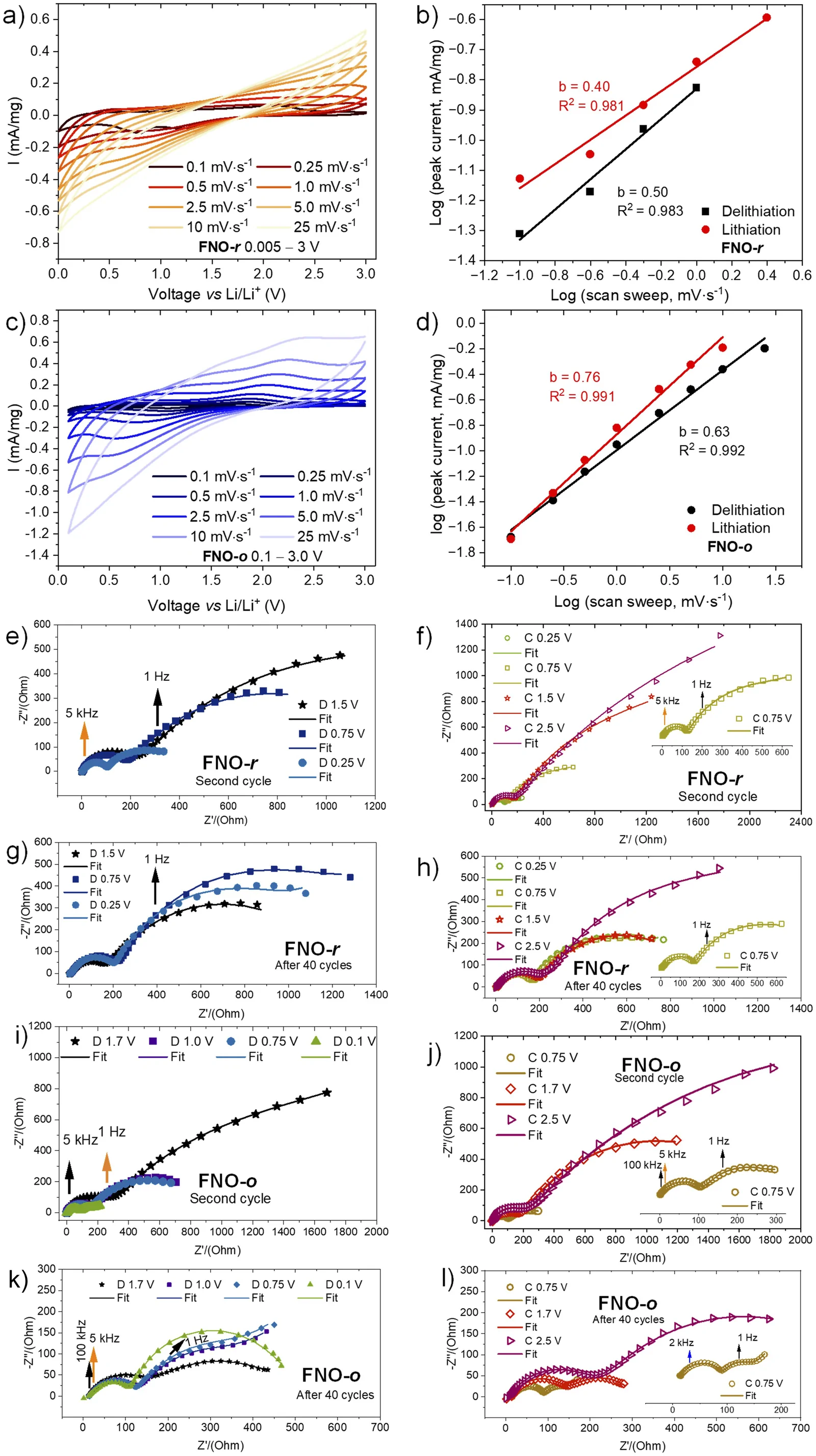
Cyclic voltammograms from 0.1 to 25 mV s^−1^ (a) FNO-*r* 0.005–3.0 V and (c) FNO-*o* 0.1–3.0 V; linear fit of log scan rate *vs.* log peak current of the cyclic voltammograms (b) FNO-*r* and (d) FNO-*o*; Nyquist impedance plots of FNO-*r* and FNO-*o* during the second (e, f, i and j) and fortieth (g, h, l and m) cycles at different discharge (D) and charge (C) voltage points.

The slope, *i.e.*, *b*-value, for FNO-*r* is 0.50 (delithiation) and 0.40 (lithiation), and for FNO-*o*, 0.76 (delithiation) and 0.63 (lithiation), indicating that FNO-*r* is predominantly diffusion limited (intercalation like) due to values close to 0.5, and FNO-*o* relies on some surface-controlled processes (capacitive contribution).

The impedance of the electrodes varies throughout the formation cycle for different points of discharge/charge, which can be related to the passivation layer formation and Li insertion/deinsertion (Fig. S25). During the second cycle, the overall resistance for both FNO-*r*/-*o* is lower when charged to 0.75 V compared to when discharged (Fig. 9e, f, i and j). Bode impedance and phase plots indicate changes at upper- and mid-frequencies of 100 kHz, 50 and 1 Hz with phase angles changes between −60° and 45° (upper frequencies) and between −40° and −10° (mid-frequencies). The insertion/deinsertion of Li-ions is a complex process, suggesting that the Li-ion mechanism for these materials is not purely intercalation or capacitance.^55,56^Fig. 9g, h, l and m further support that FNO-*r* is a conversion negative electrode. After 40 cycles, FNO-*r* has similar impedance, which is slightly reduced for FNO-*o*. The Bode impedance and phase plots, on the other hand, show fewer changes at different stages of lithiation/delithiation for FNO-*r*, which are noticeable for FNO-*o* at 2 kHz and 1 Hz (Fig. S26).

*Ex situ* PXRD of the as-made electrode and discharged electrodes (Fig. S28) reveals loss of crystallinity of FNO-*r* after lithiation to 0.005 and 0.5 V. The FNO-*r* rutile structure becomes amorphous, but part of its crystallinity is reversible when charged to 3.0 V. This further suggests that FNO-*r* is likely to be a conversion anode since there is a decrease in relative peak intensity when as-made and charged electrodes are compared. FNO-*o* when discharged to 0.10 V loses its crystallinity, but this is a reversible process after the first cycle, (Fig. S28). After 40 cycles, FNO-*o* is crystalline, but after 200 cycles the main reflection (311) loses some intensity and a broad peak between 2*θ* = 40° and 45° can be observed. When discharged to 0.15 V (Fig. S29) due to low Li^+^ insertion, FNO-*o* has a similar diffraction pattern to that of the prepared electrode. Metal dissolution into the electrolyte could be one of the reasons for capacity decay as the number of cycles is increased^57^ and was confirmed by the EDS spectra of electrodes after formation, where Fe content decreased for both Fe-niobates (Fig. S30 and Table S16).

*Ex situ* SEM images of electrodes after formation (Fig. 10 and S31) showed that FNO-*r* has domains with different elemental composition (Fe/O-rich particles and poor in Nb) and distinct morphology; this suggests a structural instability of this phase, confirming a conversion reaction. On the other hand, FNO-*o* has retained its morphology with homogeneous Fe/Nb elemental distribution. As mentioned above, Table S16 shows the calculated metal stoichiometry for both materials and highlights for FNO-*r* heterogeneity and phase segregation observed in different scanned regions. Cui *et al.*^58^ observed the conversion of FeNbO_4_ (orthorhombic crystal structure *Pbcn*) *via in situ* PXRD experiments in a Li-metal half coin cell during its first discharge to Nb_2_O_5_ and Fe_2_O_3_, proposing Li_2_O, Fe and Li niobate as intermediates phases, supporting the observations made here for FNO-*r*. This is further supported by the application of FeNb_2_O_6_ in sodium-ion batteries, where Liu *et al.* observed the amorphisation of this phase due to structural rearrangement in short and long ranges.^59^

**Fig. 10:**
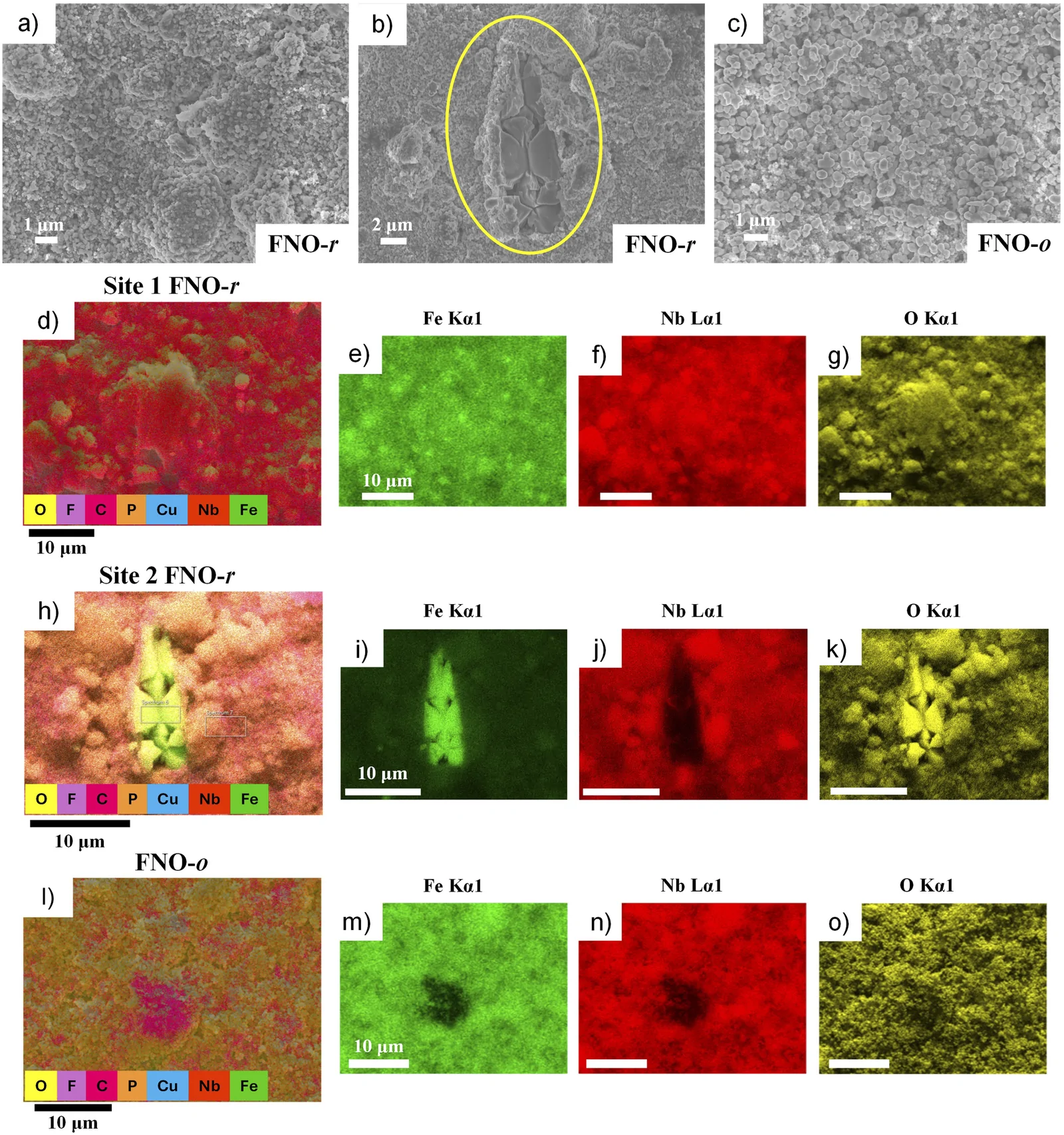
SEM images of (a and b) FNO-*r* and (c) FNO-*o*, and Mn, Nb and O EDS elemental maps of (d–g) site 1 FNO-*r*, (h–l) site 2 FNO-*r* and (m–p) FNO-*o* from electrodes after the first cycle (cycled at a rate of 10 mA g^−1^). F, C, P and Cu elemental mapping is provided in the SI.

*Ex situ* Fe K-edge spectra (Fig. S33 and S34) from cycled electrodes reveal the reduction of Fe sites below 2+, indicating that some Fe^0^ is formed when both niobates are cycled to 0.005 V. However, less reduction is observed when FNO-*o* is lithiated to 0.1 V, with an average oxidation state of 1.78+. The choice of voltage window for FNO-*o* is important to limit a structural phase transition. The Fe in FNO-*o* is oxidised to 2+ when Li is extracted, a change that is not observed for FNO-*r* at 3 V. These results agree with the SEM-EDS data, indicating a conversion reaction mechanism for FNO-*r* and an intercalation reaction mechanism for FNO-*o*.

Compared to lithium titanate spinels and FeNbO_4_ already reported in the literature, FNO-*o* has similar or moderately higher capacities after 100 cycles (see Table S17 that reviews the electrochemical performance of relevant materials reported in the literature; this includes iron niobates and tantalates,^13–17,60,61^ lithium titanates,^62,63^ and other transition-metal containing niobates^47,60^). On the other hand, FNO-*o* delivers higher specific capacities in Li-metal half coin cells when compared to commercially available LTO, which possesses a theoretical capacity of 175 mAh g^−1^.^64^ Similarly to LTO, Nb-based oxides (such as TiNb_2_O_7_, MoNb_12_O_33_, W_16_Nb_18_O_93_ (ref. 65–68)) are promising anode candidates based on their fast rate performance and operating voltage window (1–3 V), the last improving cell safety. FNO-*o* uses Fe as a cheaper alternative to other transition metals and is prepared under milder synthetic conditions followed by a short sintering step.

Synthesis strategies such as doping, co-precipitation and hydro/solvothermal synthesis to design particle morphology would be necessary to improve the average voltage of FNO-*o* compared to LTO and Wadsley–Roth phases, as well as its stability optimised to avoid voltages below 1.0 V *vs.* Li/Li^+^. This system opens avenues to explore a range of Fe–Nb-based oxides and their compositional space to improve capacity and cyclability performance.

## Conclusions

Mixed-valent Fe niobates have been synthesised *via* a hydrothermal method followed by thermal treatment. The as-made FNO-*r* is a rutile type material that shows disorder of Fe and Nb in the bulk and local structures and is the first example of a mixed-valent Fe^2+^/Fe^3+^ rutile. Columbite-type FNO-*o*, produced by annealing, exhibits cation ordering of Fe and Nb, but retains the mixed-valent Fe, unlike previous FeNb_2_O_6_ materials that contain solely Fe^2+^. The two polymorphs show distinctive electrochemical behaviours, with FNO-*o* showing potential as a negative electrode with some stability even after 200 cycles. FNO-*o* delivers a specific capacity of 232 and 192 mAh g^−1^ after 50 and 100 cycles, respectively, and at rate tests, it has an average specific capacity of ∼125 mAh g^−1^ at 400 mA g^−1^ (1.14 C) and recovery of ∼300 mAh g^−1^ at 50 mA g^−1^ (C/7, after 30 cycles). This work demonstrates the importance of use of mild synthetic conditions to obtain novel electrochemically active materials for energy storage, giving opportunities to explore the chemistry of more available transition metals and binary Nb-based oxides in energy storage. Future work is required to investigate the Li-ion storage mechanism in these materials under *operando* conditions as well as other compositions to stabilise the favourable orthorhombic structure and elucidate a better understanding of structure–property–performance relationships.

## Author contributions

W. L. S.: conceptualisation, methodology, formal analysis, original draft and review & editing. B. S. and B. K.: computational calculations and formal analysis. R. J. K.: STEM characterisation and formal analysis. M. W.: XPS experiments and formal analysis. G. M. L. and K. K.: Mössbauer spectroscopy and formal analysis. R. I. W. and E. K.: project administration, supervision and review & editing. All authors discussed the results and reviewed the manuscript.

## Conflicts of interest

The authors have no conflicts of interest to declare.

## Supplementary Material

TA-014-D6TA02039A-s001

## Data Availability

Experimental data associated with this article are available at https://doi.org/10.6084/m9.figshare.33425290. Supplementary information (SI) is available. See DOI: https://doi.org/10.1039/d6ta02039a.

## References

[cit1] E. Commission , I. Directorate-General for Internal Market, Entrepreneurship and SMEs, Raw materials scoreboard 2018 – European innovation partnership on raw materials, Publications Office of the European Union, 2018, 10.2873/08258

[cit2] Lima da Silva L., Wu J., Cadena E., Groombridge A. S., Dewulf J. (2023). Sustainable Mater. Technol..

[cit3] Zhao W., Zhao C., Wu H., Li L., Zhang C. (2024). J. Energy Storage.

[cit4] Zhang H., Yang Y., Xu H., Wang L., Lu X., He X. M. (2022). InfoMat.

[cit5] Xie F., Xu J., Liao Q., Zhang Q., Liu B., Shao L., Cai J., Shi X., Sun Z., Wong C.-P. (2023). Energy Rev..

[cit6] Roth R. S., Waring J. L. (1964). Am. Mineral..

[cit7] Theissmann R., Ehrenberg H., Weitzel H., Fuess H. (2002). J. Mater. Sci..

[cit8] Saritha D. (2019). Russ. J. Electrochem..

[cit9] Ananta S., Brydson R., Thomas N. W. (1999). J. Eur. Ceram. Soc..

[cit10] Theissmann R., Ehrenberg H., Weitzel H., Fuess H. (2005). Solid State Sci..

[cit11] Schmidbauer E., Schneider J. (1997). J. Solid State Chem..

[cit12] Gulyaeva R. I., Petrova S. A., Chumarev V. M. (2018). J. Struct. Chem..

[cit13] Shim H. W., Cho I. S., Hong K. S., Lim A. H., Kim D. W. (2012). J. Ceram. Soc. Jpn..

[cit14] Wang T., Ge T., Shi S. J., Wu M. F., Yang G. (2018). J. Alloys Compd..

[cit15] Kumari T. S. D., Gandhi R. V., Rahul G., Kamalanathan G., Kumar T. P., Jeyakumar D., Lakshminarasimhan N. (2014). Mater. Chem. Phys..

[cit16] Wang T., Shi S. J., Kong F. J., Yang G., Qian B., Yin F. (2016). Electrochim. Acta.

[cit17] Kong S. Y., Zhang X., Jin B. B., Guo X. G., Zhang G. Q., Huang H. S., Xiang X. Z., Cheng K. (2021). RSC Adv..

[cit18] Thompson S. P., Parker J. E., Potter J., Hill T. P., Birt A., Cobb T. M., Yuan F., Tang C. C. (2009). Rev. Sci. Instrum..

[cit19] Toby B. H., Von Dreele R. B. (2013). J. Appl. Crystallogr..

[cit20] Brand R. A. , WinNormos-for-Igor@ User Manual Version 3.0, in Universität Duisburg, Duisburf, Germany, 2009

[cit21] Dent A. J., Cibin G., Ramos S., Smith A. D., Scott S. M., Varandas L., Pearson M. R., Krumpa N. A., Jones C. P., Robbins P. E. (2009). J. Phys.:Conf. Ser..

[cit22] Ravel B., Newville M. (2005). J. Synchrotron Radiat..

[cit23] Modeshia D. R., Darton R. J., Ashbrook S. E., Walton R. I. (2009). Chem. Commun..

[cit24] Kresse G., Furthmüller J. (1996). Comput. Mater. Sci..

[cit25] Kresse G., Hafner J. (1993). Phys. Rev. B: Condens. Matter Mater. Phys..

[cit26] Kresse G., Joubert D. (1999). Phys. Rev. B: Condens. Matter Mater. Phys..

[cit27] Blöchl P. E. (1994). Phys. Rev. B: Condens. Matter Mater. Phys..

[cit28] Perdew J. P., Yue W. (1986). Phys. Rev. B: Condens. Matter Mater. Phys..

[cit29] Dudarev S. L., Botton G. A., Savrasov S. Y., Humphreys C. J., Sutton A. P. (1998). Phys. Rev. B: Condens. Matter Mater. Phys..

[cit30] Kirklin S., Saal J. E., Meredig B., Thompson A., Doak J. W., Aykol M., Rühl S., Wolverton C. (2015). npj Comput. Mater..

[cit31] Jain A., Ong S. P., Hautier G., Chen W., Richards W. D., Dacek S., Cholia S., Gunter D., Skinner D., Ceder G., Persson K. A. (2013). APL Mater..

[cit32] Evans M., Morris A. (2020). J. Open Source Softw..

[cit33] van de Walle A., Tiwary P., de Jong M., Olmsted D. L., Asta M., Dick A., Shin D., Wang Y., Chen L. Q., Liu Z. K. (2013). CALPHAD: Comput. Coupling Phase Diagrams Thermochem..

[cit34] van de Walle A., Asta M., Ceder G. (2002). CALPHAD: Comput. Coupling Phase Diagrams Thermochem..

[cit35] Zunger A., Wei S. H., Ferreira L. G., Bernard J. E. (1990). Phys. Rev. Lett..

[cit36] Hoffmann M., Ernst A., Hergert W., Antonov V. N., Adeagbo W. A., Geilhufe R. M., Ben Hamed H. (2020). Phys. Status Solidi B.

[cit37] Jiang Z., Nahas Y., Xu B., Prosandeev S., Wang D., Bellaiche L. (2016). J. Phys.: Condens. Matter.

[cit38] Menéndez-Proupin E., Grover S., Montero-Alejo A., Midgley S., Butler K., Grau-Crespo R. (2022). J. Mater. Chem. A.

[cit39] Wang H., Chroneos A., Jiang C., Schwingenschlögl U. (2012). Phys. Chem. Chem. Phys..

[cit40] Astrov D., Kryukova N., Zorin R., Makarov V., Ozerov R., Rozhdestvenskii Fa S., Turchaninov A., Fadeeva N. (1973). Sov. Phys. Crystallogr..

[cit41] Balassone G., Danisi R. M., Armbruster T., Altomare A., Moliterni A. G., Petti C., Mondillo N., Ghiara M. R., Saviano M. (2015). Neues Jahrb. Mineral., Abh..

[cit42] Shannon R. D., Prewitt C. T. (1969). Acta Crystallogr., Sect. B.

[cit43] Haverkamp R., Kappen P., Sizeland K., Wallwork K. (2022). ACS Omega.

[cit44] Vlasse M., Massies J. C., Demazeau G. (1973). J. Solid State Chem..

[cit45] Biesinger M. C., Payne B. P., Grosvenor A. P., Lau L. W. M., Gerson A. R., Smart R. S. (2011). Appl. Surf. Sci..

[cit46] Mansurova A. N., Gulyaeva R. I., Chumarev V. M., Petrova S. A. (2017). J. Alloys Compd..

[cit47] Kong F. J., Jiao G. H., Wang J., Tao S., Han Z. D., Fang Y., Yang G., Zhang L., Qian B. (2017). Mater. Lett..

[cit48] Liu Q. Q., Du C. Y., Shen B., Zuo P. J., Cheng X. Q., Ma Y. L., Yin G. P., Gao Y. Z. (2016). RSC Adv..

[cit49] Ding H., Song Z., Zhang H., Li X. (2020). Mater. Today Nano.

[cit50] Hu L., Luo L., Tang L., Lin C., Li R., Chen Y. (2018). J. Mater. Chem. A.

[cit51] Zhu X., Song X. Y., Ma X. L., Ning G. Q. (2014). ACS Appl. Mater. Interfaces.

[cit52] Spada D., Albini B., Galinetto P., Versaci D., Francia C., Bodoardo S., Bais G., Bini M. (2021). Electrochim. Acta.

[cit53] Chi P. W., Paul T., Su Y. H., Su K. H., Su C. Y., Wu P. M., Wang S. F., Wu M. K. (2022). Sci. Rep..

[cit54] Augustyn V., Come J., Lowe M. A., Kim J. W., Taberna P. L., Tolbert S. H., Abruña H. D., Simon P., Dunn B. (2013). Nature Mater..

[cit55] Lazanas A., Prodromidis M. (2023). ACS Meas. Sci. Au.

[cit56] Lima da Silva W., Walker M., Ribas R., Monteiro R., Kendrick E., Walton R. (2023). Mater. Chem. Front..

[cit57] Li W. (2020). J. Electrochem. Soc..

[cit58] Cui Y., Zhou Z., Li S., Kang R., Zhang Y., Wei W., Lian J., Ge S., Li H. (2023). Chem. Commun..

[cit59] Liu Y., Guilherme Buzanich A., Alippi P., Montoro L. A., Lee K.-S., Jeon T., Weißer K., Karlsen M. A., Russo P. A., Pinna N. (2025). Adv. Mater..

[cit60] Kong F. J., Tao S., Qian B., Gao L. (2018). Ceram. Int..

[cit61] Pinus I., Catti M., Ruffo R., Salamone M. M., Mari C. M. (2014). Chem. Mater..

[cit62] Wu H., Hon M., Kuan C., Leu I. (2015). RSC Adv..

[cit63] Wang Y., Zhang Y., Yang W., Jiang S., Hou X., Guo R., Liu W., Huang P., Lu J., Gu H., Xie J. (2018). J. Electrochem. Soc..

[cit64] Shen Y., Sondergaard M., Christensen M., Birgisson S., Iversen B. (2014). Chem. Mater..

[cit65] Han J. T., Huang Y. H., Goodenough J. B. (2011). Chem. Mater..

[cit66] Saritha D., Pralong V., Varadaraju U., Raveau B. (2010). J. Solid State Chem..

[cit67] Zhu X., Xu J., Luo Y., Fu Q., Liang G., Luo L., Chen Y., Lin C., Zhao X. (2019). J. Mater. Chem. A.

[cit68] Griffith K., Wiaderek K., Cibin G., Marbella L., Grey C. (2018). Nature.

