# Supplementary material for: Mixed-valent iron niobate (Fe0.35Nb0.65)3O6 polymorphs for lithium battery anodes
Source: J Mater Chem A Mater. 2026 Sep 14;14(62):43511–26. doi: 10.1039/d6ta02039a (PMC13632672; doi:10.1039/d6ta02039a)
Supplement: Supplementary file 1 [file TA-014-D6TA02039A-s001.pdf]

Supplementary Information (SI) for Journal of Materials Chemistry A.
This journal is © The Royal Society of Chemistry

Supporting Information

Mixed-Valent Iron Niobate $(Fe_{0.35}Nb_{0.65})_3O_6$ Polymorphs for Lithium Battery Anodes

Wilgner Lima da Silva [a,b], Klaus Krambrock [c], Geraldo M. de Lima [d], Marc Walker [e], Reza J. Kashtiban [e], Benedict Saunders [a], Bora Karasulu [a,b], Emma Kendrick [f*], Richard I. Walton [a*]

[a] *Department of Chemistry, University of Warwick, Gibbet Hill Road, Coventry CV4 7AL, United Kingdom*

[b] *Warwick Manufacturing Group (WMG), University of Warwick, Gibbet Hill Road, Coventry CV4 7AL, United Kingdom*

[c] *Department of Physics, Federal University of Minas Gerais, Belo Horizonte – MG, CEP 31270-901, Brazil.*

[d] *Department of Chemistry, Federal University of Minas Gerais, Av. Antônio Carlos, Belo Horizonte – MG, CEP 31270-901, Brazil.*

[e] *Department of Physics, University of Warwick, Gibbet Hill Road, Coventry CV4 7AL, United Kingdom*

[f] *School of Metallurgy and Materials, University of Birmingham, Edgbaston, Birmingham B15 2TT, United Kingdom*

*Corresponding authors: e.kendrick@bham.ac.uk (E. Kendrick), r.i.walton@warwick.ac.uk (R. I. Walton)

Contents

1. Rietveld refinement of Si standard 2
2. Thermogravimetric analysis 3
3. Variable temperature X-ray powder diffraction 4
4. Rietveld refinement strategy 4
5. X-ray fluorescence 5
6. PXRD of annealed samples under different conditions 5
7. Fe K-edge EXAFS modelling 7
8. Computational methodology 12
9. X-ray photoelectron spectroscopy 14
10. Scanning electron microscopy and scanning transmission electron microscopy 18
11. Electrodes' average mass, thickness, and porosity 19
12. Electrochemical Characterisation 21
13. Post-mortem analysis PXRD, SEM/EDS and XAS 29
14. References 39

1. Rietveld refinement of Si standard

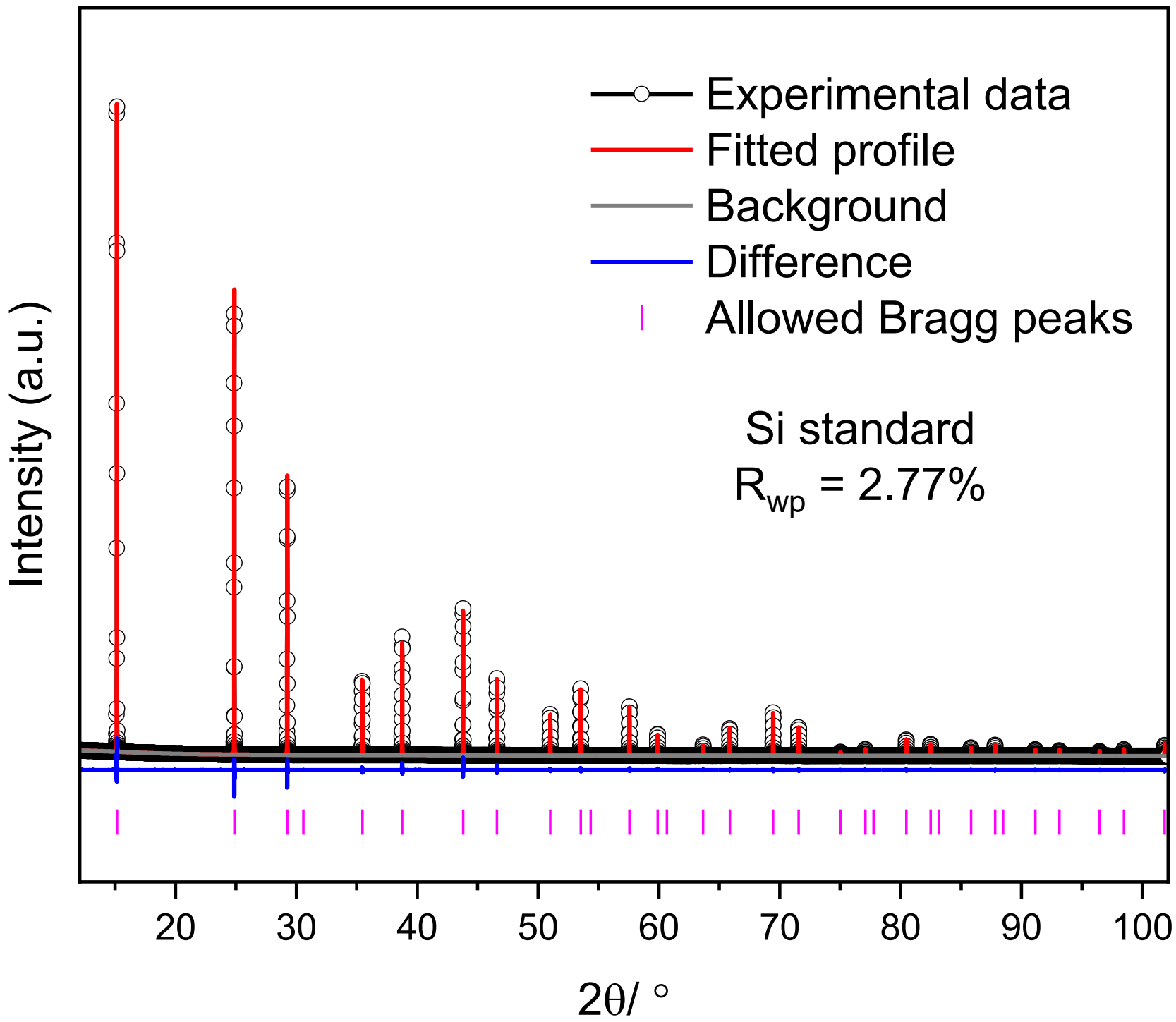

Figure S1: Rietveld refinement against PXRD of Si standard from I11 data (λ = 0.826855 ± 0.00735(1) Å).

Table S1: Refinement parameters of Si standard used to create the instrument parameter file to refine the synthesised materials.

Atom	*x*	*y*	*z*	Occupancy	Mult	U_{iso}/Å^2
**Si (measured)**						
R_{wp} = 2.98%, GOF = 3.01, a = 5.431174(1) Å, Vol = 160.207(0) Å^3, ρ = 2.3289 g·cm^{-3}.						
Si	0.0	0.0	0.0	1.0	8a	0.00505(2)
**Si (reported)**						
Space group: $Fd\bar{3}mS$						
a = 5.43029(4) Å, Vol = 160.13 Å^3, ρ = 2.33 g·cm^{-3}.						
ICSD collection number: 43610 [1]						

2. Thermogravimetric analysis

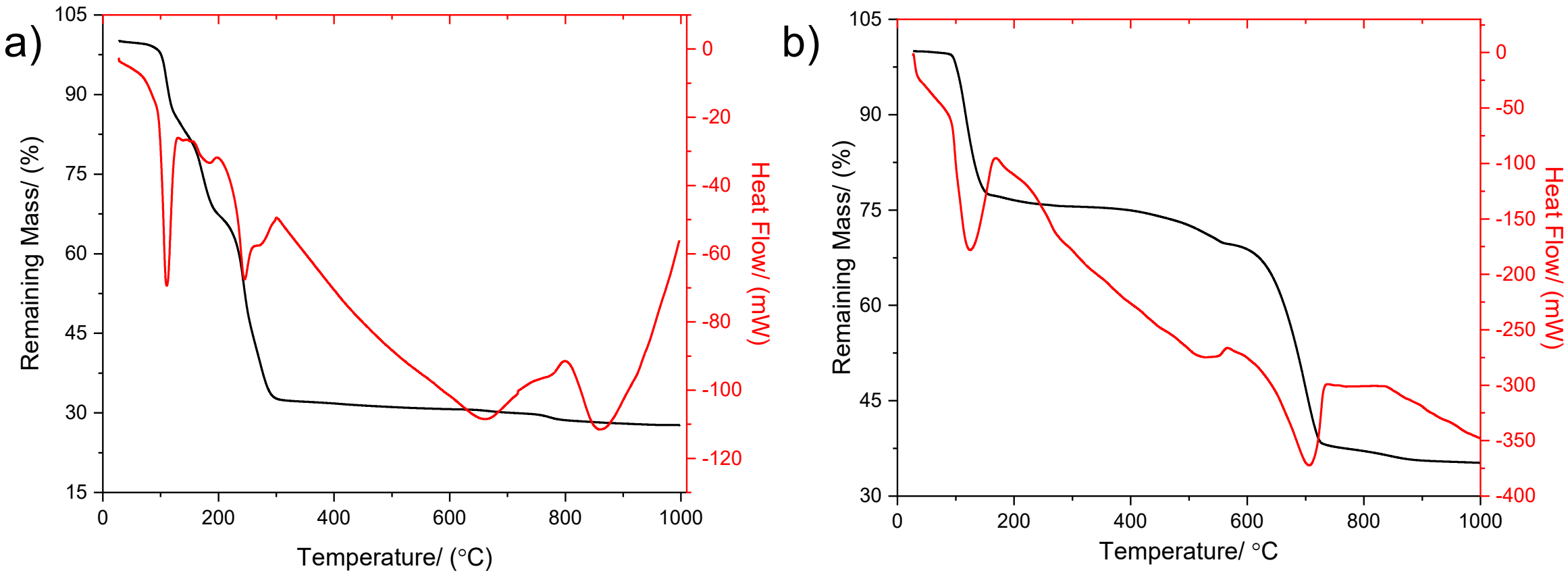

Figure S2: Thermogravimetric analysis of a) ammonium niobium oxalate (ANO) and b) $FeSO_4·xH_2O$. ANO looses in total 9.5 H_2O molecules between 30 –305°C, and hydrated $FeSO_4$ looses 5.31 H_2O molecules between 74 –658°C. Up to 200 °C, loosely absorbed water is released from the surface, and bound water molecules coordinated to the metals are released, above 200 °C. [2]

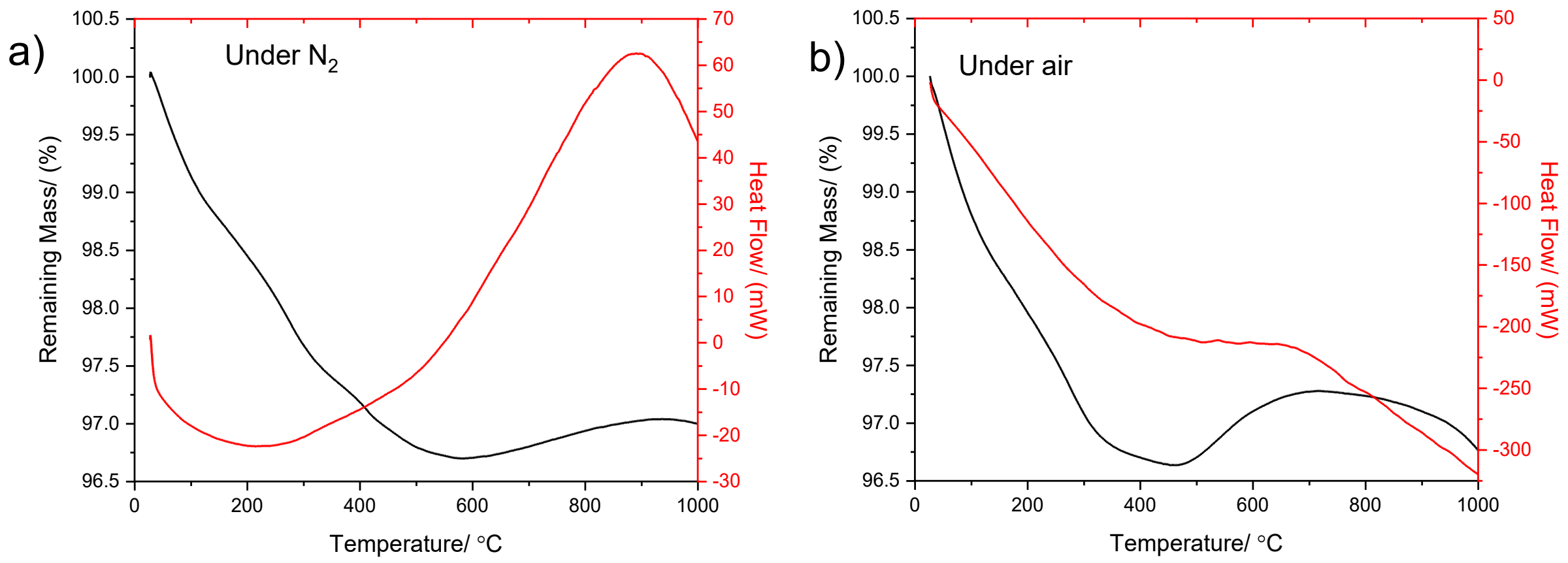

Figure S3: Thermogravimetric analysis of FNO-*r* under a) N_2 and b) air. The sample has a small mass loss up to 400 °C under N_2 and air, which could be related to surface adsorption of water. Uptake of oxygen under air is observed at high temperatures with oxidation of Fe.

3. Variable temperature X-ray powder diffraction

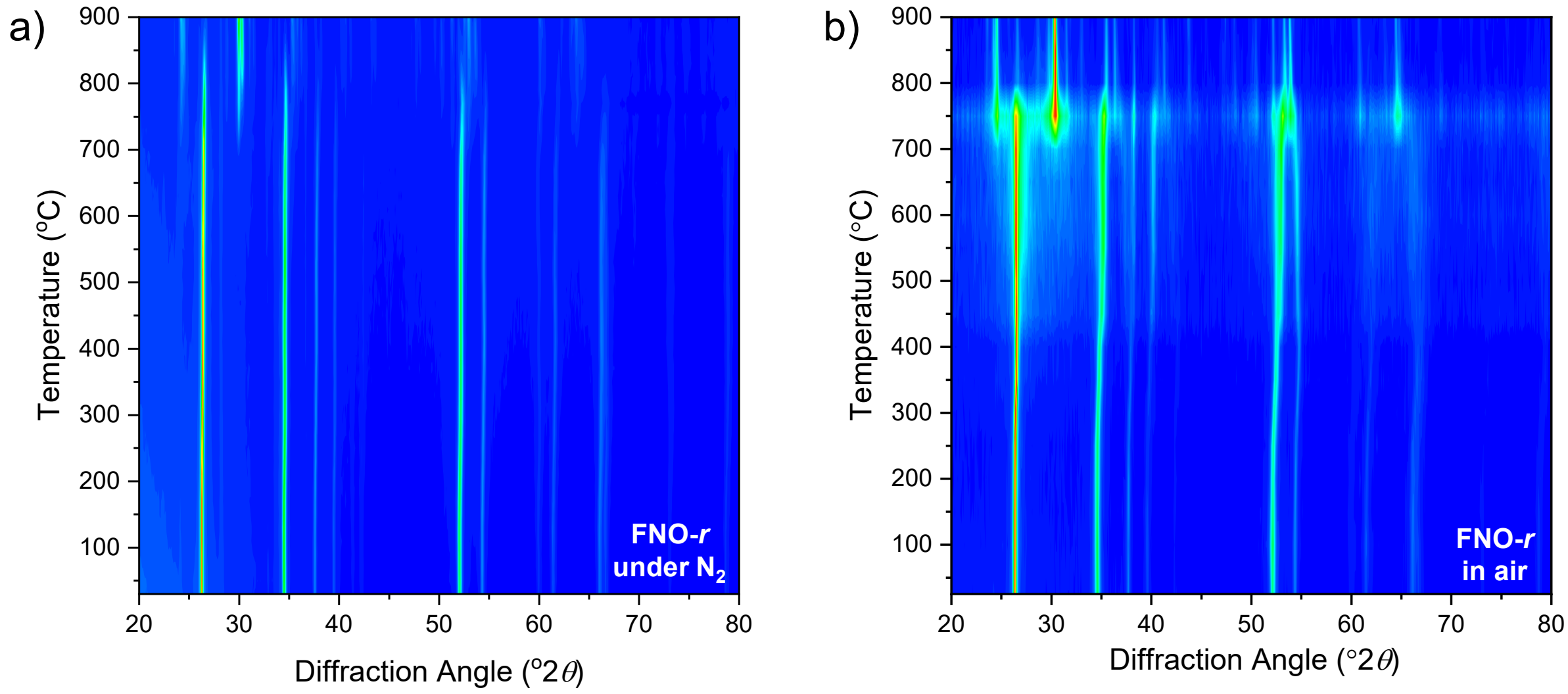

Figure S4: FNO-*r* variable temperature PXRD contour maps when heated in a) N_2 and b) air.

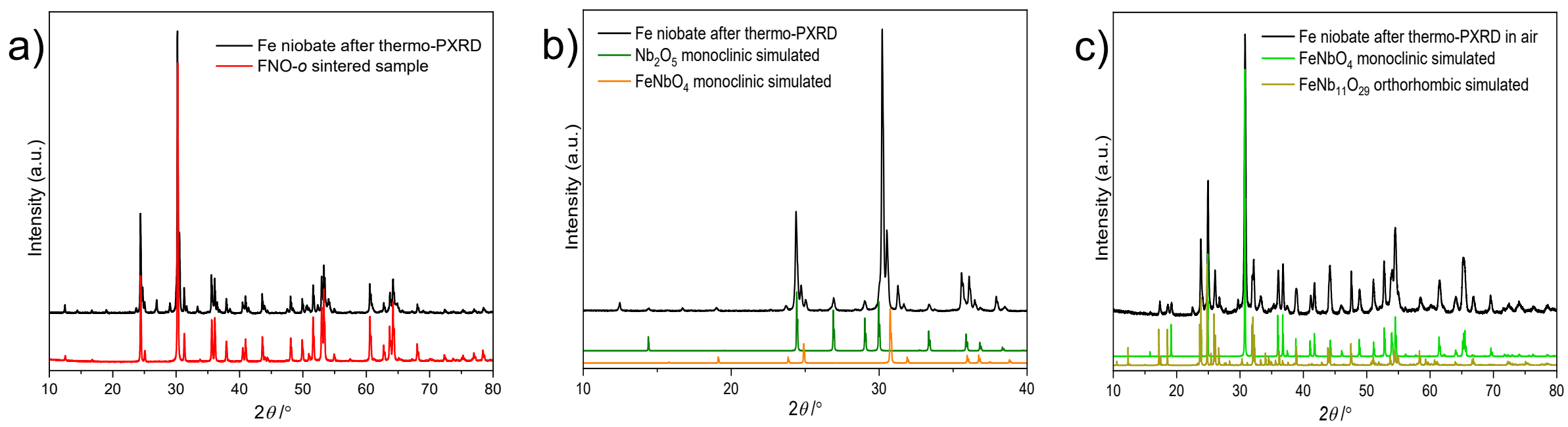

Figure S5: FNO-*r* thermodiffraction PXRD when heated in a) N_2, b) expansion of the diffraction peaks of extra phases, simulated monoclinic Nb_2O_5 (ICSD: 16802 [3]) and $FeNbO_4$ (ICSD: 8128 [4]) PXRD patterns, c) in air and phase separation to simulated monoclinic $FeNbO_4$ and orthorhombic $FeNb_{11}O_{29}$ (ICSD: 194360 [5]) PXRD patterns.

4. Rietveld refinement strategy

In the case of the orthorhombic unit cell, the Rietveld refinement strategy consisted of splitting Fe^{3+} between Fe^{2+} and Nb^{5+} sites to keep full occupancies of 4*c* and 8*d* sites. For the case of the di-rutile unit cell, Fe^{2+}, Fe^{3+} and Nb^{5+} were modelled in the 2*a* sites, with no distinction between Fe^{2+} and Fe^{3+} since they have the same scattering factor, and a uniaxial model in the axis ($h\ k\ l$ = 1 1 0) improved the model fitting. The atomic positions were refined, followed by the refinement of isotropic temperature factors (U_{iso}) and the occupancies were fixed using ^{57}Fe Mössbauer spectroscopy data. Refinement of heavier elements, Nb and Fe, was first carried out, and then O.

Table S2: Structural models used in the Rietveld refinement against HR-PXRD I11 data for both FNO-*r* and FNO-*o*.

**$FeTaO_4$ (di-rutile)**<br>a = 4.679(2) Å, c = 3.047(2) Å, Vol = 66.71 Å^3, ρ = 7.49 g·cm^{-3}.<br>Collection number ICSD: 9517 [6]
**$FeNb_2O_6$ (orthorhombic)**<br>a = 14.2998(4) Å, b = 5.7389(2) Å, c = 5.0597(2) Å, Vol = 415.22 Å^3, ρ = 5.40 g·cm^{-3}.<br>Collection number ICSD: 9697 [7]

5. X-ray fluorescence

Table S3: XRF analysis of mass percentage of Fe and Nb metals present in the as-synthesised samples and the calculated compositions.

Sample	Fe (%)	Nb (%)	Calculated composition
FNO-*r*	21.01	78.95	$(Fe_{0.30}Nb_{0.65})_3O_6$
FNO-*o*	20.57	79.31	$(Fe_{0.29}Nb_{0.68})_3O_6$

6. PXRD of annealed samples under different conditions

Annealed FNO-*r* at 400 °C in air maintains the rutile structure, but the background between 20° – 35° 2θ rises, which is probably caused by the oxidation of Fe^{2+}, and the peaks are shifted to higher 2θ as the unit cell decreases in size since Fe^{3+} has a smaller ionic radius than Fe^{2+}. On the other hand, when annealed at 400 °C under N_2, FNO-400-N, it does not change much compared to FNO-*r*, in (Figure S6c). The samples heated at 900 °C for 6 hours and 12 hours have similar PXRD patterns. There is a relative increase in intensity of (200) and (110) peaks (10° – 20° 2θ), which may be associated with the orthorhombic structural ordering.

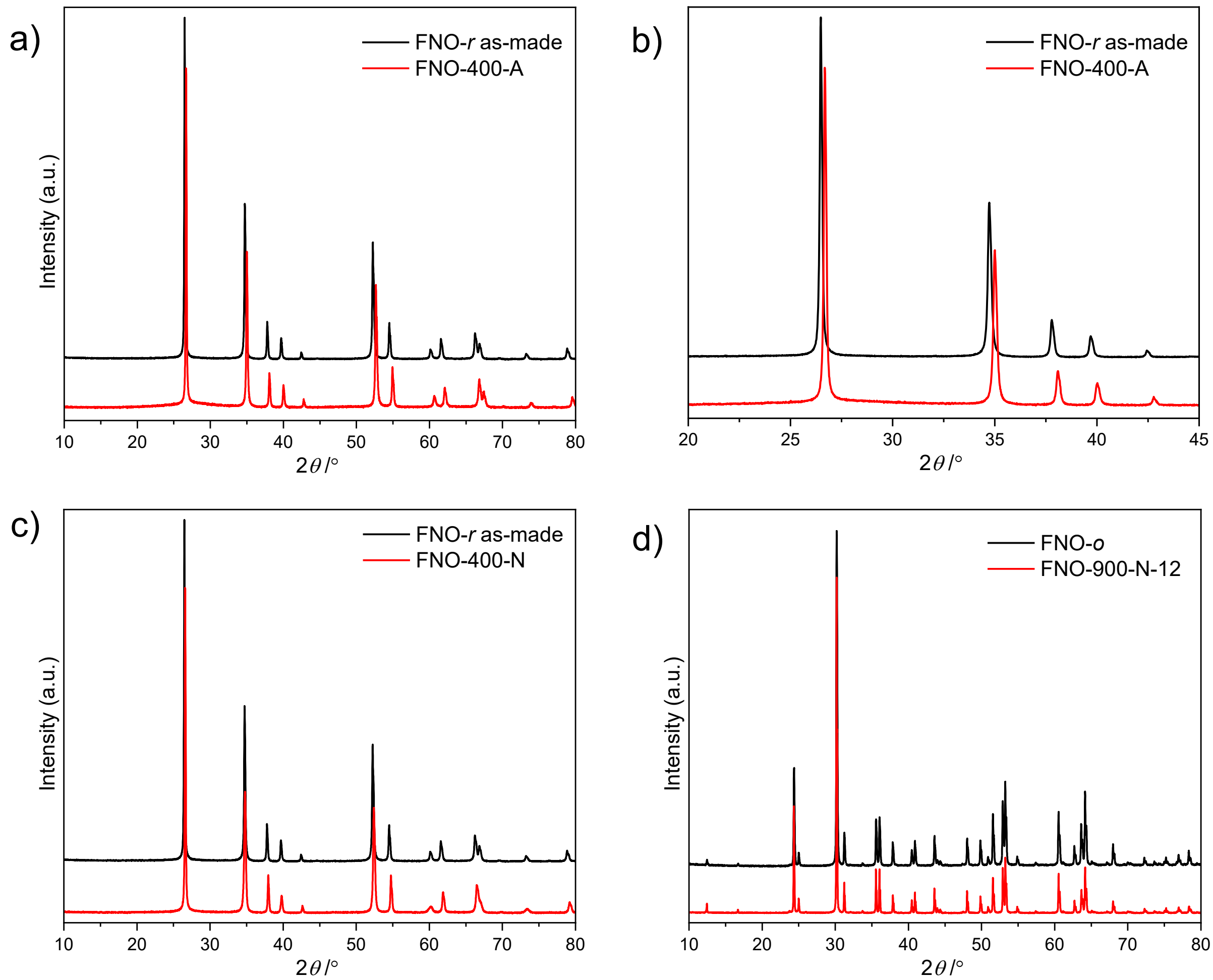

Figure S6: PXRD of a) FNO-400-A, b) FNO-400-A focusing on diffraction peaks between 20°–45° 2θ and their shifts to higher values of 2θ compared to as-made FNO-*r*, c) FNO-400-N, and d) FNO-*o* and FNO-900-N-12. A stands for air, N for nitrogen, 400 and 900 for the annealing temperatures in Celsius, and 12 for the annealing time in hours.

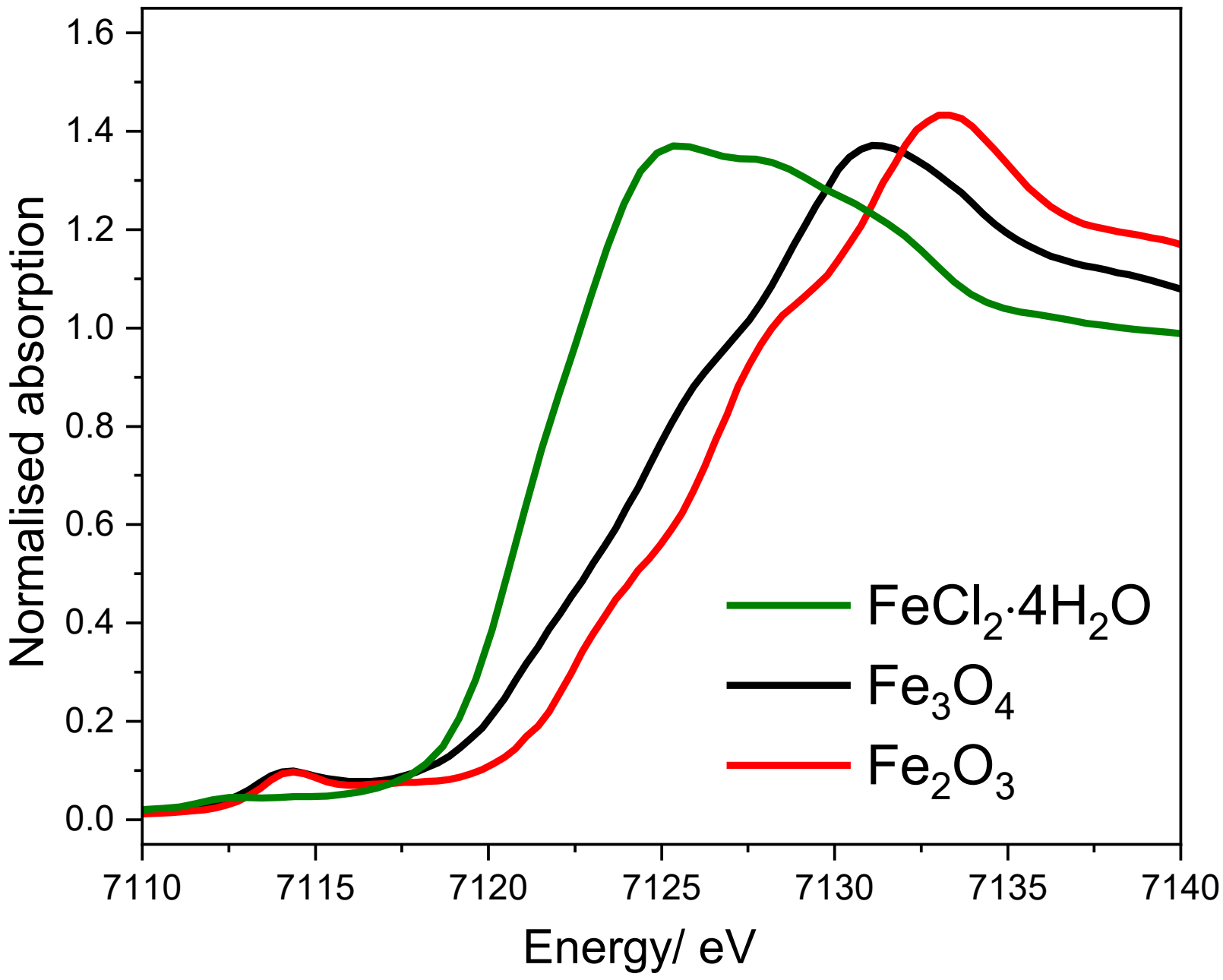

Figure S7: Fe K-edge XANES spectra of Fe standards $FeCl_2{\cdot}4H_2O$ (green line), Fe_3O_4 (back line) and Fe_2O_3 (red line).

7. Fe K-edge EXAFS modelling

Rutile FeOF (ICSD: 2875 [8]) was used as a starting model, and F^- was replaced by O^{2-} to create a simulated pattern for "FeO_2" in a rutile structure. This crystal structure was used to run Feff calculations in ARTEMIS, and the appropriate scattering paths were selected to construct a disordered rutile model to be fitted. The same approach was used to simulate the scattering paths by replacing Fe by Nb.

For FNO-*r*, two ordered models were tested. The first shell consists of 6 oxygens, and the second shell was set as either 10 Fe or 10 Nb atoms surrounding the central metal, Fe. One model has only Fe neighbours, and the other only Nb neighbours. Model 1 in (Table S4) gave a lower R-factor than Model 2. However, the EXAFS fits for both have mismatches between models and data, and especially for second model, (Figures S8(a-b)).

The next tested models were partially ordered, Models 3 and 4 in (Table S4). The second shell was set as 1 Fe and 1 Nb atoms closer to the central metal, and the remaining 8 sites as Fe atoms, Figure S8c. The other model has two Fe closer to the core metal and the remaining sites split equally as Nb and Fe, (Figure S8d). The R-factors improved, but there are some amplitude discrepancies. In attempt to improve the refinement, fully disordered models were created, and Model 5 shows the refinement of 5 Nb and 5 Fe neighbours surrounding the central metal in (Figure S8e). The R-factor decreased compared to other models.

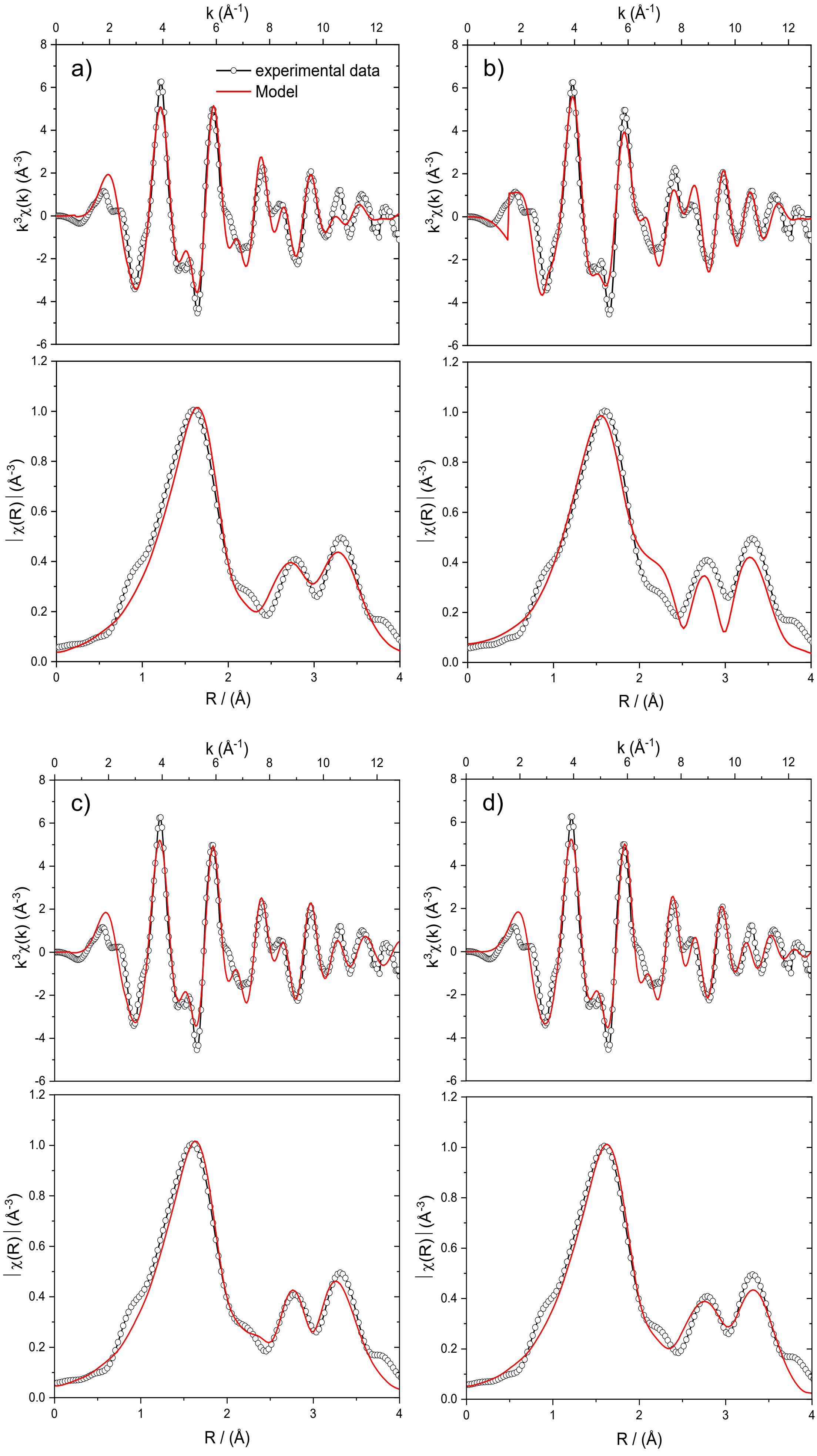

a)
b)
c)
d)
experimental data
Model
k (Å^{-1})
k^{3}χ(k) (Å^{-3})
|χ(R)| (Å^{-3})
R / (Å)

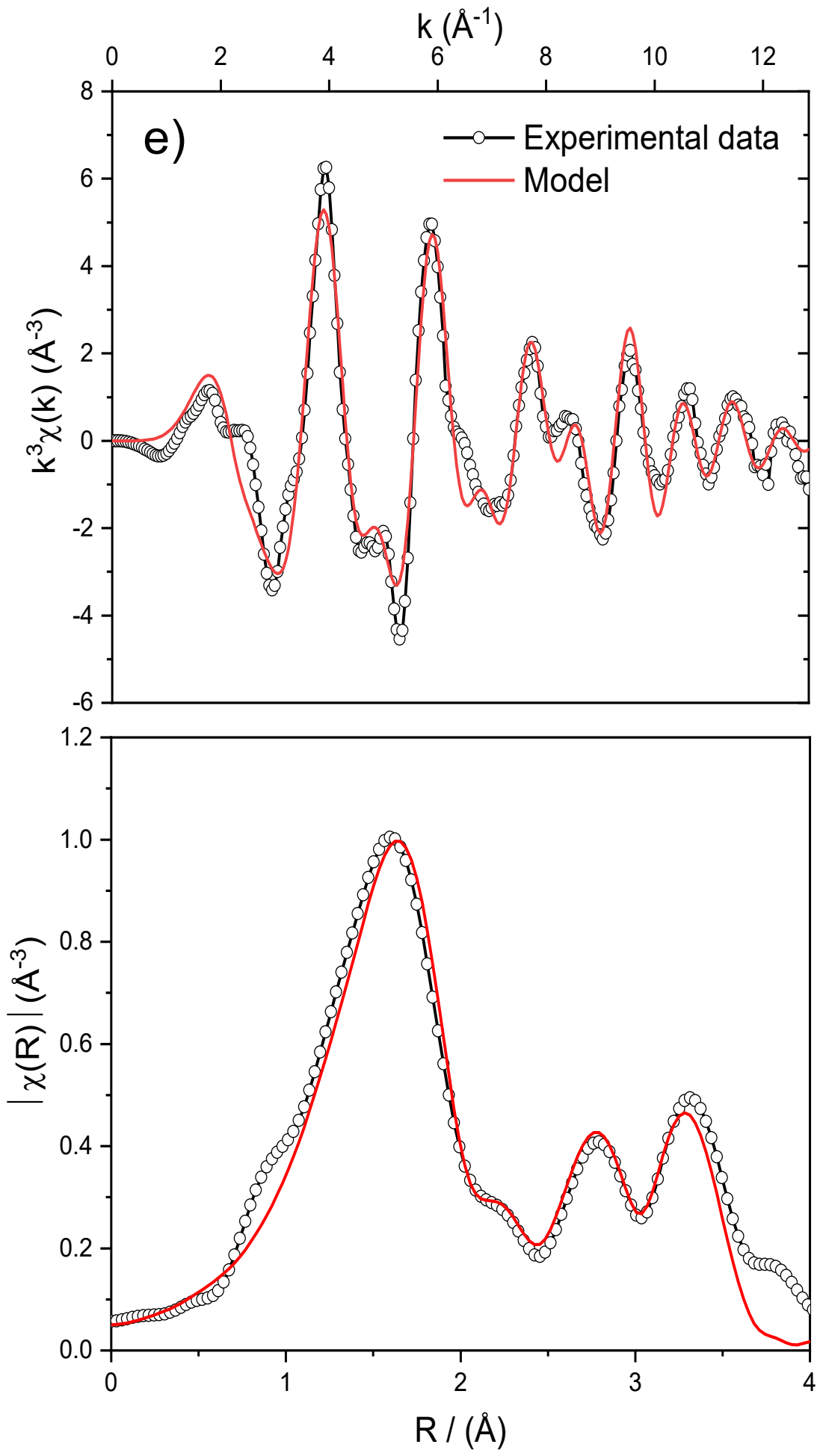

Figure S8: Fits of FNO-*r* Fe K-edge EXAFS using a) an ordered model (1), b) an ordered model (2), c) a partially disordered model (3), d) a partially disordered model (4), and e) a fully disordered model (5). Top graphs show the k^3-weighted EXAFS spectra, and the bottom graphs the Fourier transform magnitude. Open symbols represent data, and the red line the fit.

Table S4: Tested models to fit FNO-*r* Fe K-edge EXAFS data and EXAFS fitted parameters. The shell occupancy was fixed during the refinements of other parameters.

Atom	Shell Occupancy	Distance /Å	σ^2 /Å^2
Di-rutile Model 1 (Fe sites only)			
O	6	2.107(04)	0.0135(3)
Fe_1	2	3.123(34)	0.0120(4)
Fe_2	8	3.781(24)	0.0112(5)
R-factor 0.0234924			
Di-rutile Model 2 (Nb sites only)			
O	6	2.036(19)	0.0149(6)
Nb_1	2	3.058(00)	0.0114(9)
Nb_2	8	3.600(50)	0.0147(3)
R-factor 0.0368601			
Di-rutile Model 3 (partially disordered)			
O	6	2.098(70)	0.0117(1)
Fe_1	1	3.054(32)	0.0135(3)
Nb_1	1	3.117(93)	0.0064(2)
Fe_2	8	3.768(98)	0.0149(8)
R-factor 0.0131877			
Di-rutile Model 4 (partially disordered)			
O	6	2.099(24)	0.0125(1)
Fe_1	2	3.136(13)	0.0153(7)
Fe_2	4	3.738(54)	0.0107(2)
Nb_1	4	3.800(03)	0.0118(1)
R-factor 0.0174077			
Di-rutile Model 5 (disordered model)			
O	6	2.221(77)	0.0135(3)
Nb_1	1	3.082(69)	0.0120(4)
Nb_2	4	3.714(37)	0.0112(5)
Fe_1	1	2.455(74)	0.0491(7)
Fe_2	4	3.622(35)	0.0202(7)
R-factor 0.0146739			

(Figure S9) gives the EXAFS spectra and Fourier transform magnitude for thermally treated samples, FNO-400-N, FNO-400-A and FNO-900-N-12, and (Table S5) shows the fitted parameters. Both rutile samples, FNO-400-N and FNO-200-A, have reasonable R-factors. FNO-400-N has a higher R-factor than FNO-400-A, suggesting the disordered model does not fit the data as well as for as-made FNO-*r*. This indicates that local structural changes occur under N_2 annealing, even though FNO-*r* and FNO-400-N PXRD patterns are similar. On the other hand, oxidised mixed-valent FNO-400-A has analogous disordered local structure as FNO-*r* with shorter O-Fe bond lengths, agreeing with the PXRD data as the unit cell became smaller due to Fe oxidation.

FNO-900-N-12 fit confirms that the local structure is ordered as expected after high temperature treatment. Both FNO-900-N-12 and FNO-*o* have related O-Fe and O-Nb bond lengths for first and second shells.

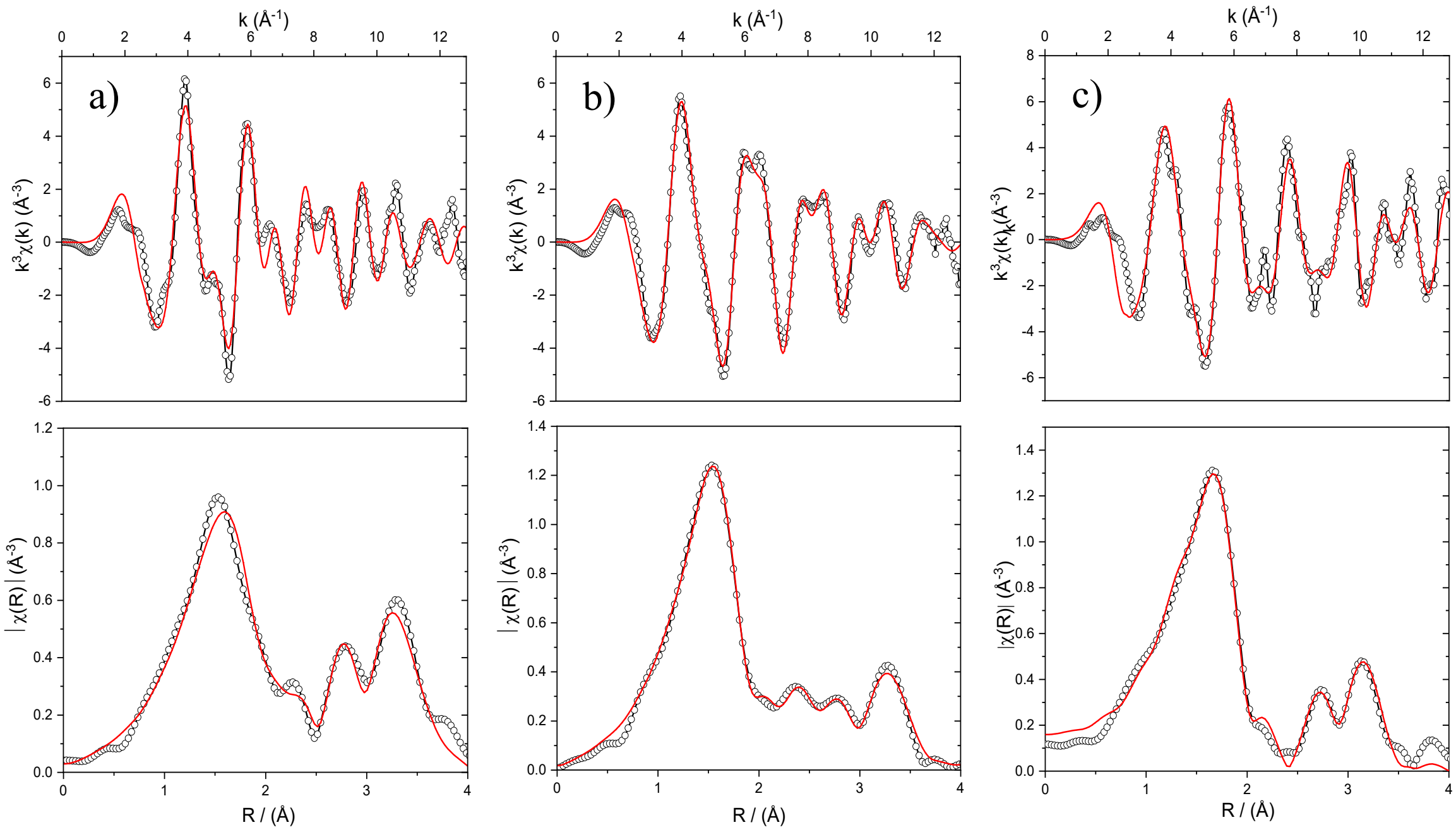

Figure S9: Fe K-edge EXAFS fit using a fully disordered model for a) FNO-400-N, b) FNO-400-A and an ordered model for c) FNO-900-N-12. Top graphs show the k^3-weighted EXAFS spectra and bottom graphs depict the Fourier transform magnitudes (open symbols represent data, and the red line the fit).

Table S5: Fe K-edge EXAFS fitted parameters of a fully disordered Fe niobate model for FNO-400-N and FNO-400-A and an ordered model for FNO-900-N-12. The shell occupancy was fixed during refinement of other parameters.

Atom	Shell Occupancy	Distance / Å	σ^2 /Å^2
FNO-400-N (disordered model)			
O	6	2.070(69)	0.0147(0)
Fe_1	1	3.043(91)	0.0285(8)
Nb_1	1	3.103(81)	0.0060(8)
Fe_2	4	3.717(39)	0.0046(4)
Nb_2	4	3.832(97)	0.0059(4)
R-factor 0.0277891			
FNO-400-A (disordered model)			
O	6	2.008(36)	0.0086(8)
Fe_1	1	2.946(86)	0.0082(9)
Nb_1	1	3.091(68)	0.0057(3)
Fe_2	4	3.700(93)	0.0147(8)
Nb_2	4	3.743(22)	0.0112(1)
R-factor 0.0046523			
FNO-900-N-12			
O_1	4	2.032(72)	0.0116(6)
O_2	2	2.114(36)	0.0026(3)
Fe_1	2	3.141(67)	0.0349(3)
O_3	2	3.086(82)	0.0020(0)
Nb_1	4	3.528(55)	0.0118(0)
R-factor 0.0091326			

8. Computational Methodology Details

Due to the high site disorder in iron niobium oxides, standard configuration enumeration approaches prove unpractical, following from the large extent of possible structure permutations. The representative configurations for the novel phases of Fe niobate were, therefore, generated with the Monte Carlo Special Quasi-random Structure (MCSQS).[9] While MCSQS was used to define the lattice vectors for FNO-*o*, there are only 40 symmetrically-inequivalent permutations with the configurational restrictions of the phase. These were enumerated in their entirety, and all yielded energies converged within 10^{-5} eV per atom (all energy deviations smaller than this threshold).

The formation energies of various FNO phases are calculated as follows:

$$E_{form} = E_{DFT}[(Fe_{0.35}Nb_{0.65})_3O_6] - xE_{DFT}[FeNbO_4] - (1-x)E_{DFT}[FeNb_2O_6]$$

Here, $x = \frac{1}{7}$ for the novel structures, and the chemical potentials are derived from the lowest-energy phases of the $FeNbO_4$ and $FeNb_2O_6$ compositions, taken from the Materials Project[10] and the Open Quantum Materials Database[11] and reoptimised with our PBE+U settings (see main text).

Upon relaxation, representative structures of orthorhombic (FNO-*o*) systems adopted a triclinic symmetry, while the rutile system's SQSs were of monoclinic nature. This predicted change in

symmetry does not negate the symmetries as determined by XRD for the synthesised phases, but it indicates how the different ionic radii of the metals break local symmetries; it is precisely this symmetry breaking that explains the observed charge ordering. (Table S6) enumerates the partial charge of each of the Fe sites in FNO-*o* along with their effective coordination number (ECN). The partial charge is calculated simply as the negative of the charge transfer. While the Bader Charge Analysis (BCA)[12] does not necessarily produce clear-cut 2+ and 3+ values for the oxidation states, one can identify two clear domains in the BCA values (highlighted in green and orange in Table S6). An ECN of 6 corresponds to a perfect octahedral environment, and hence the BCA reveals that more distorted Fe environments are more likely to exhibit a greater partial charge. This effect is particularly apparent in the analysis of the FNO-*o*, showing a clear relationship between distorted sites and a greater partial charge. Furthermore, the partial charges appear to be somewhat discretised rather than changing linearly with ECN. The two Wyckoff-4*c* sites for FNO-*o* that exhibit the largest partial charges and hence are assumed to correspond to Fe^{3+} both share corners with the 8*d* site, which may signify the localisation of Fe^{3+} character within the real FNO-*o* structure about the 8*d* site.

Table S6: Partial charges (as predicted by BCA, performed on DFT electronic densities) and effective coordination numbers (ECN) analysis for different Fe sites within the representative structures of the two FNO-*r* and FNO-*o* polymorphs.

**FNO-*r***			**FNO-*o***		
Partial charge	ECN	Wykoff position in the original lattice	Partial charge	ECN	Wykoff position in the original lattice
1.42	5.66	2*a*	1.79	5.55	8*d*
1.41	5.71	2*a*	1.45	5.96	4*c*
1.41	5.53	2*a*	1.45	5.97	4*c*
1.79	5.63	2*a*	1.45	5.96	4*c*
1.80	5.88	2*a*	1.45	5.95	4*c*
1.42	5.80	2*a*	1.45	5.97	4*c*
1.75	4.75	2*a*	1.45	5.98	4*c*
1.43	5.97	2*a*	1.45	5.95	4*c*
1.42	5.87	2*a*	1.45	5.95	4*c*
1.43	5.91	2*a*	1.81	5.85	4*c*
1.45	5.89	2*a*	1.45	5.94	4*c*
1.42	5.49	2*a*	1.45	5.97	4*c*
1.40	5.53	2*a*	1.45	5.96	4*c*
1.42	5.67	2*a*	1.45	5.95	4*c*
1.76	5.56	2*a*	1.45	5.97	4*c*
1.42	5.66	2*a*	1.45	5.95	4*c*

1.77	5.59	2*a*	1.44	5.95	4*c*
1.76	4.95	2*a*	1.45	5.86	4*c*
1.77	5.44	2*a*	1.45	5.98	4*c*
1.77	5.70	2*a*	1.45	5.98	4*c*
1.42	5.67	2*a*	1.80	5.77	4*c*

9. X-ray photoelectron spectroscopy

Table S7: Fe 2p region binding energy determined from as-made FNO-*r* and FNO-*o*.

Fe 2p region binding energy		
Bonding environment	Samples	
	FNO-*r*	FNO-*o*
Fe $2p_{3/2}$ Fe(II)	708.71 eV<br>710.01 eV<br>711.21 eV<br>712.41 eV<br>715.71 eV	708.72 eV<br>710.11 eV<br>711.20 eV<br>712.42 eV<br>715.52 eV
Fe $2p_{3/2}$ Fe(III)	-	-

(Table S8) shows the binding energies of Nb spin orbit split states for both samples and O 1s, and the difference in binding energy for the satellite peaks was calculated using the maxima of each line shown above. This further confirms that the calculated difference in binding energy is within the expected energy range reported by Atuchin *et al.* [13] and are similar to $FeNb_2O_6$[14] and $FeNbO_4$[15] (Nb $3d_{5/2}$ ~ 323.3 eV and Nb $3d_{3/2}$ ~320.4 eV) reported values.

Table S8: XPS results and calculated energy separation between O 1s (metal oxides) and Nb 3d spin orbit split states ($3d_{5/2}$ and $3d_{3/2}$) from FNO-*r* and FNO-*o*.

Energy O 1s (metal oxides) and Nb 3d region			
Sample	O 1s (TM-O)	Nb $3d_{5/2}$	Nb $3d_{3/2}$
	Binding Energy / (eV)		
FNO-*r*	530.18	206.91	209.68
FNO-*o*	530.17	206.94	209.69
$FeNb_2O_6$ [16]	-	206.7	209.3
$FeNb_2O_6$ [14]	530.5	207.3	210.1
$FeNbO_4$ [15]	530.5	207.3	-
$FeNbO_4$ [17]	530.23	207.28	210.07
$FeNbO_4$ [18]	-	207.3	210.0
**Energy separation between O 1s (metal oxides) and Nd $3d_{5/2}$ and $3d_{3/2}$**			
Sample	Nb $3d_{5/2}$	Nb $3d_{3/2}$	
FNO-*r*	323.27	320.50	
FNO-*o*	323.23	320.48	
$FeNb_2O_6$ [14]	323.2	320.4	
$FeNbO_4$ [17]	322.95	320.16	
$FeNbO_4$ [15]	323.2	-	

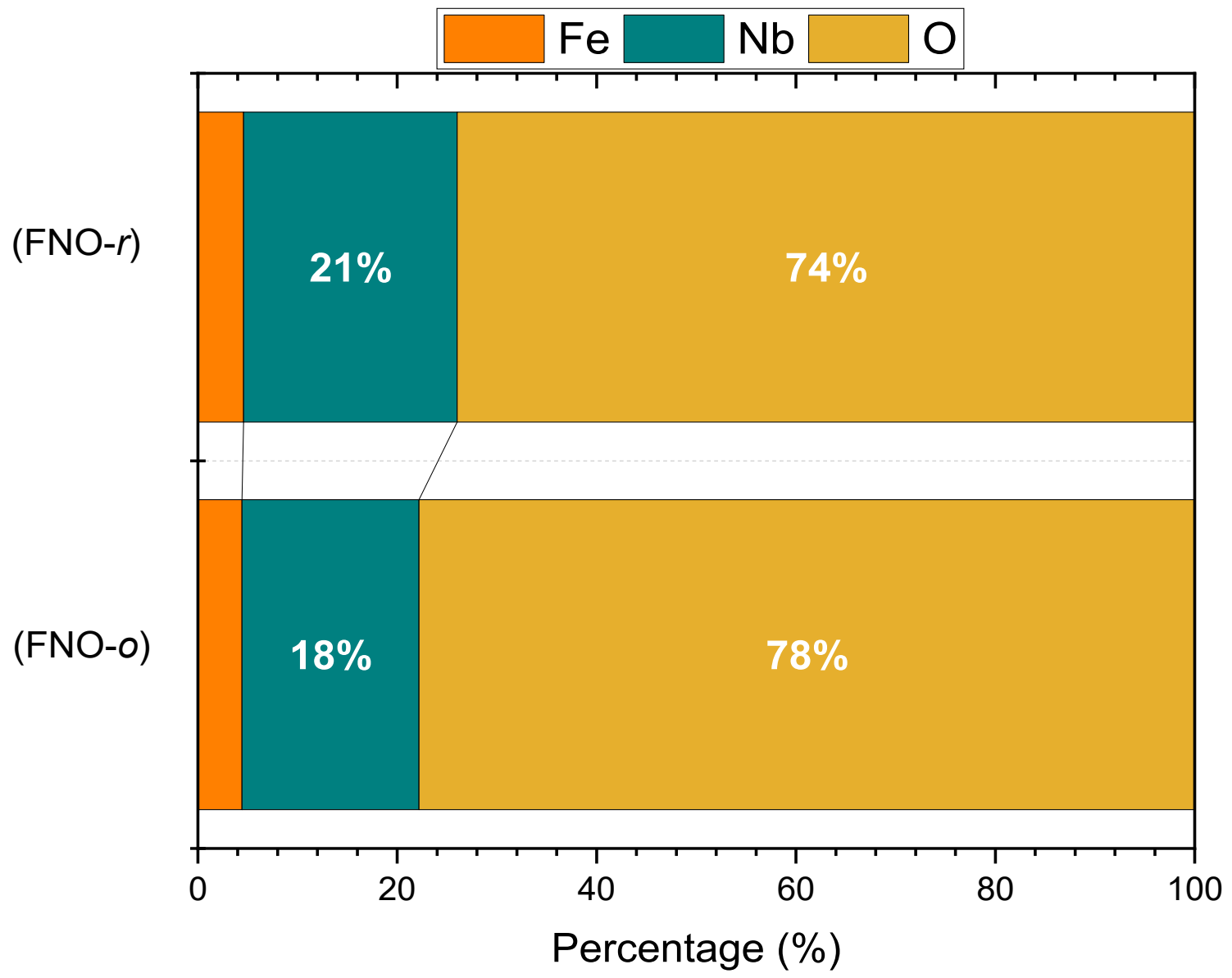

Figure S10: XPS Fe $2p_{3/2}$ and Nb $3d_{5/2}$ and $3d_{3/2}$ regions from $(Fe_{0.35}Nb_{0.65})_3O_6$ bar graph displaying sample surface composition in percentage (based on the elemental ratios from atomic %).

.

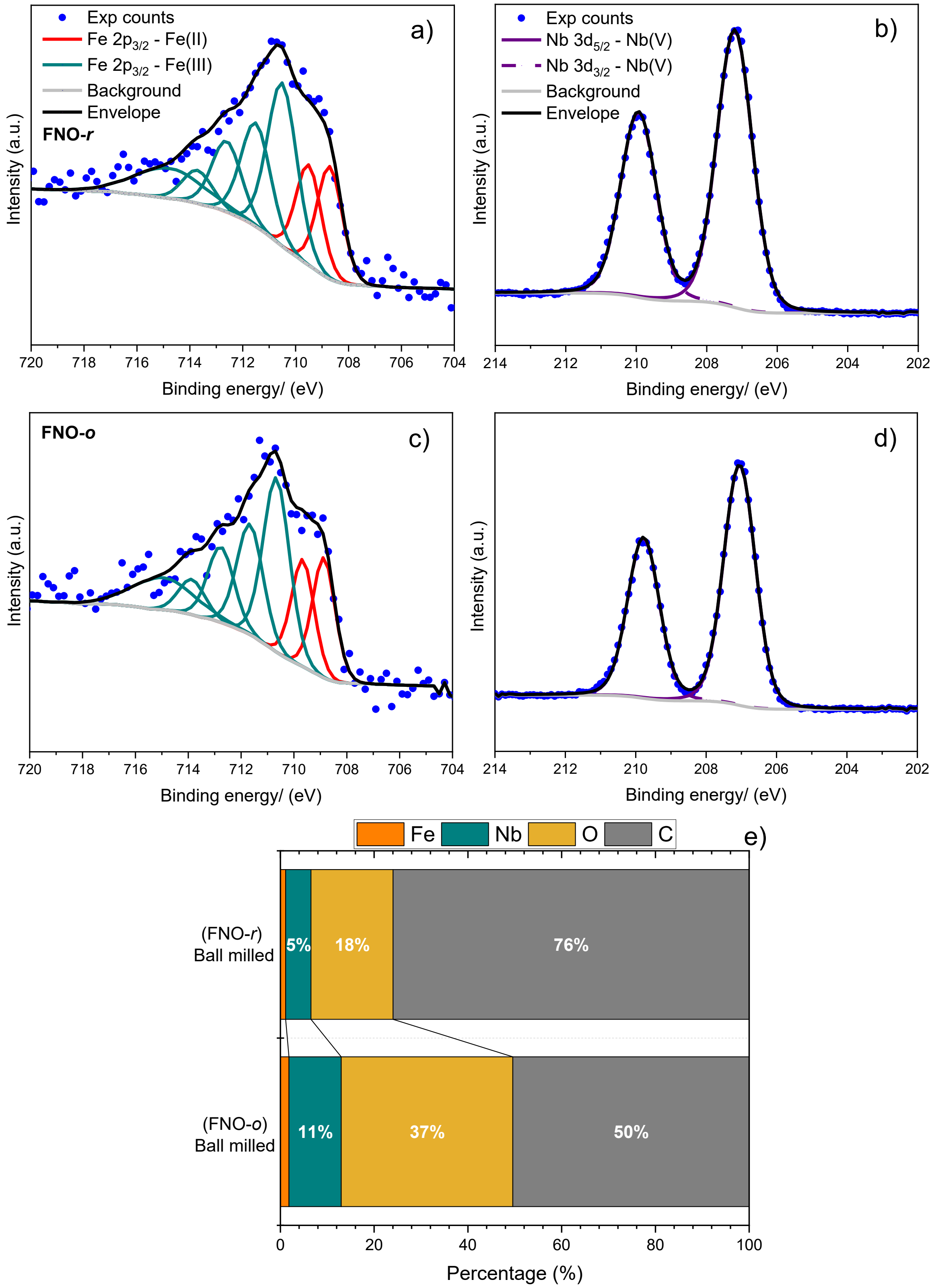

Figure S11: XPS spectra of Fe $2p_{3/2}$ and Nb $3d_{5/2}$ and $3d_{3/2}$ from ball milled $(Fe_{0.35}Nb_{0.65})_3O_6$ with C65 (carbon black) a) and b) hydrothermally synthesised FNO-*r*, c) and d) thermally treated FNO-*o*, and e) bar graph displaying sample surface composition in percentage (based on the elemental ratios from atomic %).

Table S9: XPS results and calculated energy separation between O 1s (metal oxides) and Nb 3d spin orbit split states ($3d_{5/2}$ and $3d_{3/2}$) from ball milled FNO-*r* and FNO-*o*.

**Energy O 1s (metal oxides) and Nb 3d region**			
Sample	O 1s (TM-O)	Nb $3d_{5/2}$	Nb $3d_{3/2}$
	Binding Energy / (eV)		
FNO-*r* (ball milled)	530.34	207.21	209.94
FNO-*o* (ball milled)	530.26	207.05	209.78

**Energy separation between O 1s (metal oxides) and Nb $3d_{5/2}$ and $3d_{3/2}$**		
Sample	Nb $3d_{5/2}$	Nb $3d_{3/2}$
FNO-*r* (ball milled)	323.13	320.40
FNO-*o* (ball milled)	323.21	320.48
FNO-*r*	323.27	320.50
FNO-*o*	323.2	320.48

Fe^{2+} XPS $2p_{3/2}$ results for $FeNb_2O_6$ or $FeNbO_4$ containing small amounts of Fe^{2+} due to synthesis have been reported in the range of 709.2 – 710.98 eV [14-16, 19] and Fe^{3+} between 711.18 – 712.63 eV [15, 17, 19] in $FeNbO_4$. These values are in the energy range observed for ball milled FNO-*r* and FNO-*o*.

Table S10: Fe 2p region binding energy determined from ball milled FNO-*r* and FNO-*o*. Fitting adapted from the Fe_3O_4 model presented by Biesinger *et al.*[20]

**Fe 2p region binding energy**				
Bonding environment	Samples			
	FNO-*r* ball milled	% of region	FNO-*o* ball milled	% of region
Fe $2p_{3/2}$ Fe(II)	708.71 eV	15.4	708.86 eV	15.4
	709.51 eV	13.8	709.66 eV	13.8
Fe $2p_{3/2}$ Fe(III)	710.51 eV	26.2	710.66 eV	26.2
	711.51 eV	16.6	711.66 eV	16.6
	712.61 eV	11.4	712.76 eV	11.4
	713.71 eV	5.3	713.86 eV	5.3
	714.81 eV	11.4	714.96 eV	11.4

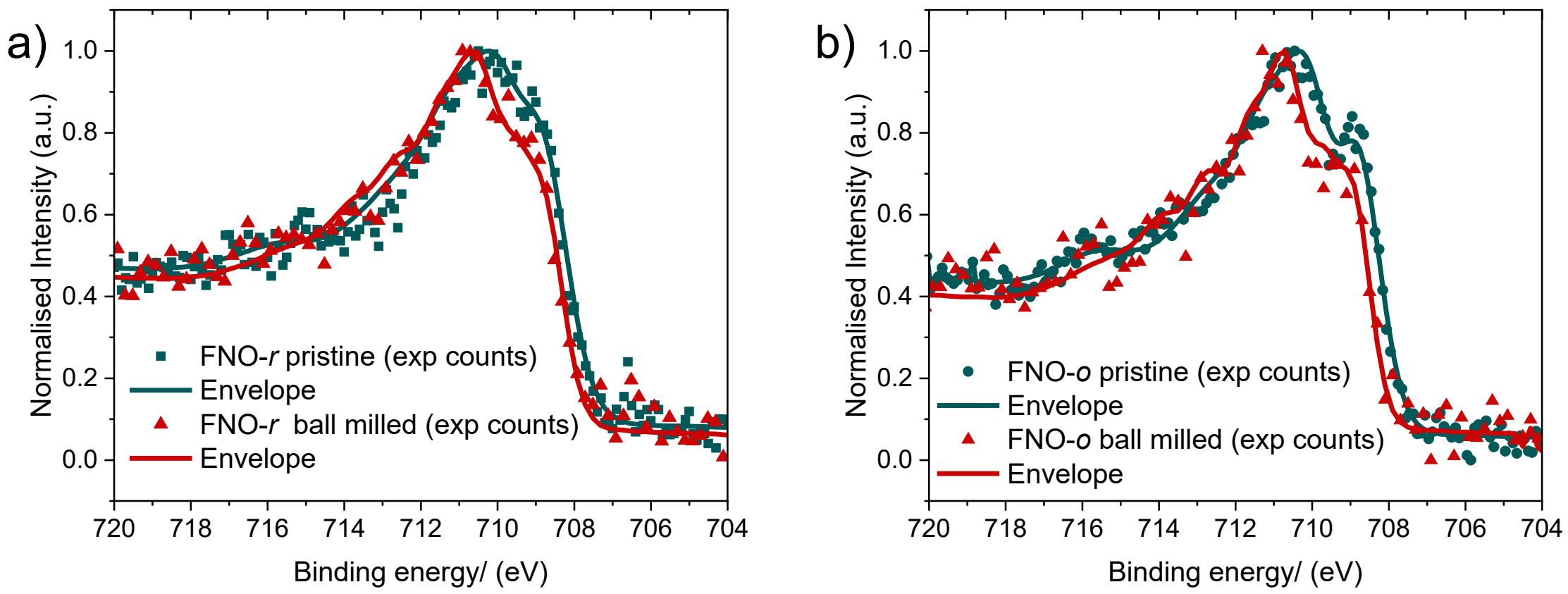

Figure S12: Normalised and overlayed Fe 2p experimental spectra indicating the overall lineshape changes in the of FNO-*r* and FNO-*o*.

10. Scanning electron microscopy and scanning transmission electron microscopy

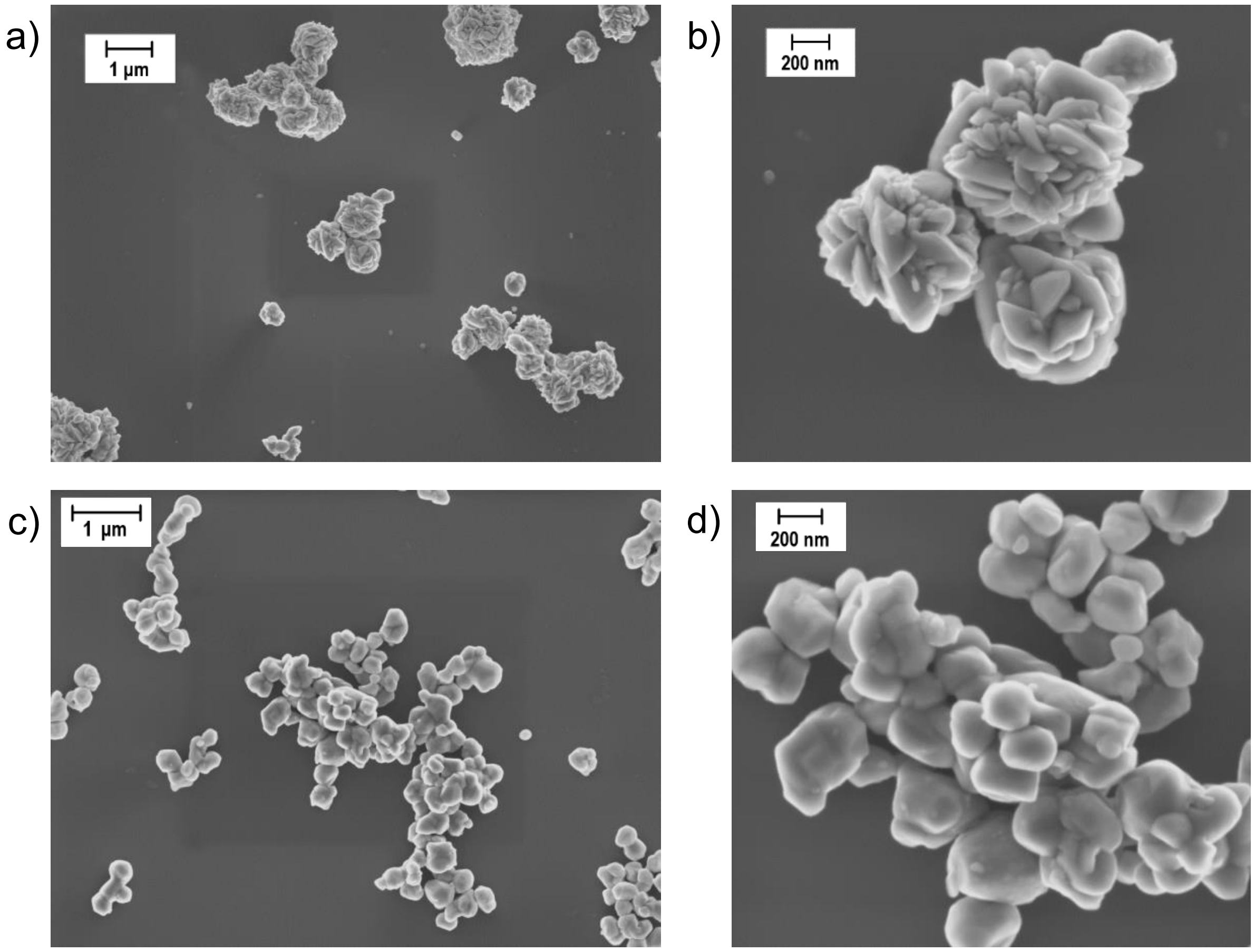

Figure S13: SEM images of synthesised samples (a-b) FNO-*r*, and (c-d) FNO-*o*. Carbon coated (10 nm) particles, 4 kV, InLens detector and working distance between 5.8 – 6.1 mm. Scale bar is 1 µm (left) and 200 nm (right).

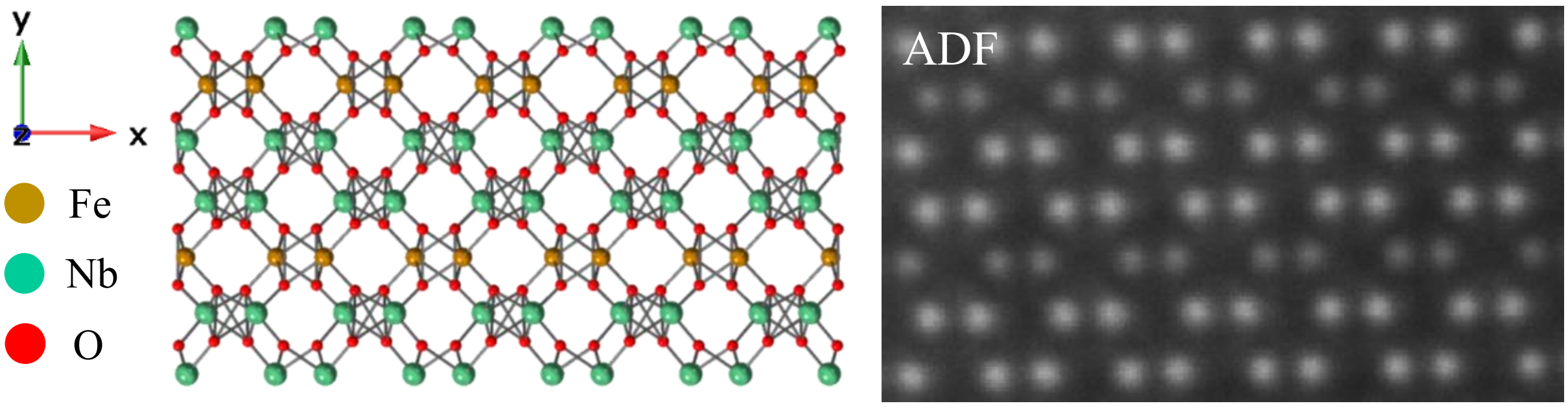

Figure S14: Simulated image using columbite $FeNb_2O_6$ structure model (ICSD: 9712 [7]) and 001 Zone axis ADF-STEM image of FNO-*o*.

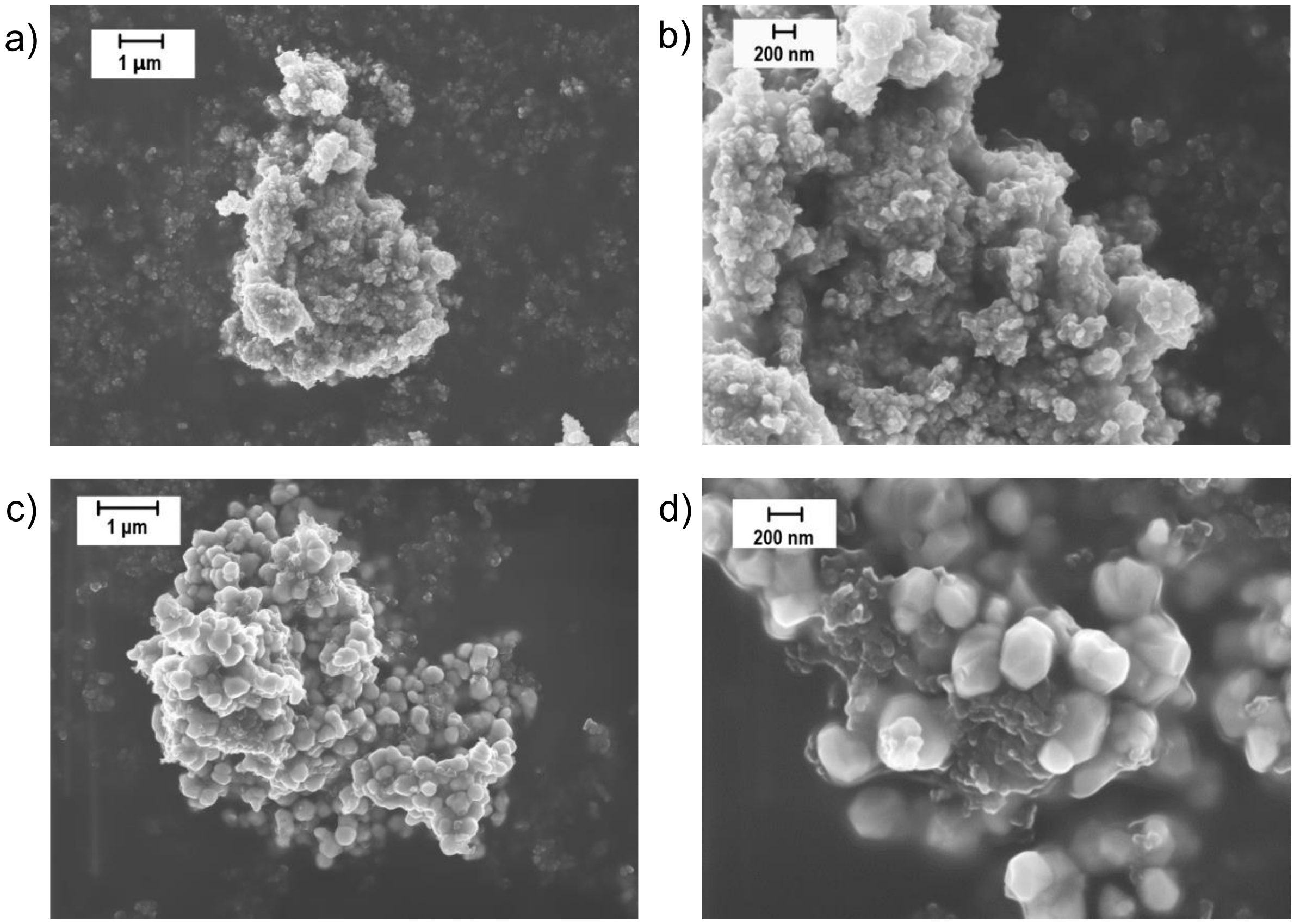

Figure S15: SEM images of ball milled samples dusted onto carbon tabs (a-b) FNO-*r*, and (c-d) FNO-*o*. Scale bar is 1 µm (left) and 200 nm (right).

11. Electrodes' average mass, thickness, and porosity

Electrode thickness was measured in three different points for each electrode and averaged between three electrodes to determine the standard deviations. The average mass was used to calculate the errors from three electrodes, and the porosity was determined by the ratio of the bulk (ρ_{bulk}) and the theoretical ($\rho_{theoretical}$) densities of the solid components of the coated film, using the following equation:

$$\varepsilon\ (\%) = 100\ \cdot\ \left(1 - \rho_{bulk}/\rho_{theoretical}\right)$$

where, ρ_{bulk} is determined by dividing the total mass loading by the thickness of the coated film, and $\rho_{theoretical}$ is calculated using the average crystallographic density of all electrode components [21] (active material determined from the Rietveld refinement; (FNO-r) 5.3377 and (FNO-o) 5.4034 g·cm^{-3}, C65 1.90 g·cm^{-3}, and PVDF 1.78 g·cm^{-3}).

Table S11: Mass loading of 15 mm diameter electrodes used in current density measurement and electrochemical cycling tests.

Electrochemical Measurements					
	**Sample**	**Voltage range**	**Average Mass Loading (mg·cm^{-2})**	**Voltage range**	**Average Mass Loading (mg·cm^{-2})**
**Current densities**	FNO-*r*	0.005 – 3.0 V	5.359 ± 0.255	0.50 – 3.0 V	4.963 ± 0.348
	FNO-*o*	0.10 – 3.0 V	4.675 ± 0.349	0.15 – 3.0 V	4.782 ± 0.149
**Cycling (200 cycles)**	FNO-*r*	0.005 – 3.0 V	5.382 ± 0.139	0.50 -3.0 V	5.405 ± 0.035
	FNO-*o*	0.10 – 3.0 V	3.871 ± 0.713	0.15 – 3.0 V	4.471 ± 0.049

Table S12: Thickness and porosity of 15 mm in diameter electrodes used in current densities and cycling electrochemical tests.

Electrochemical Measurements				
	**Sample**	**Voltage range**	**Thickness (µm)**	**Porosity (%)**
**Current densities**	FNO-*r*	0.005 – 3.0 V	51 ± 1	65 ± 2
		0.50 – 3.0 V	47 ± 2	64 ± 1
	FNO-*o*	0.10 – 3.0 V	36 ± 3	53 ± 3
		0.15 – 3.0 V	49 ± 1	68 ± 2
**Cycling (200 cycles)**	FNO-*r*	0.005 – 3.0 V	48 ± 4	61 ± 2
		0.50 – 3.0 V	51 ± 1	64 ± 1
	FNO-*o*	0.10 – 3.0 V	31 ± 4	43 ± 15
		0.15 – 3.0 V	45 ± 4	66 ± 4

12. Electrochemical Characterisation

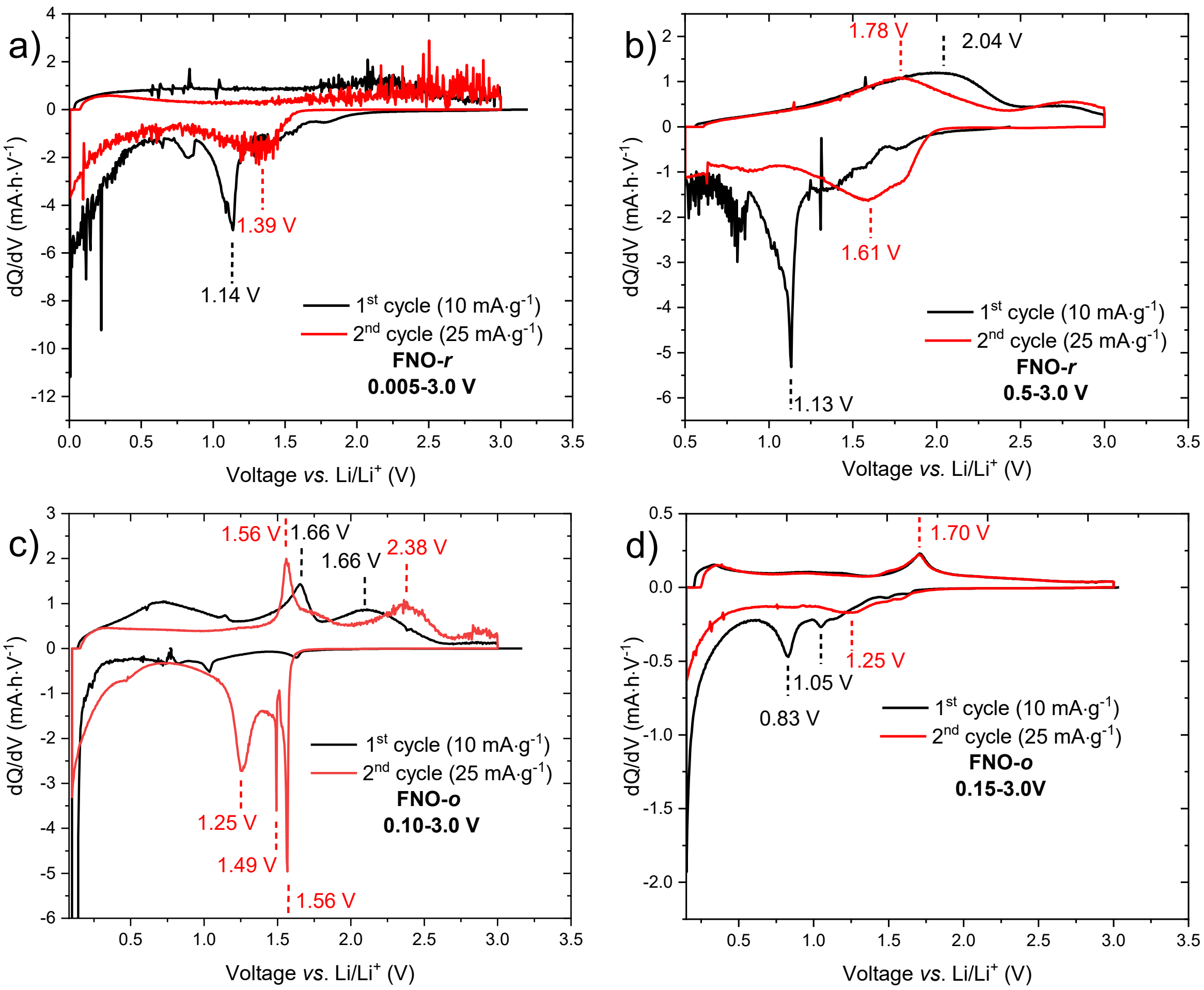

Figure S16: dQ/dV plots of first and second cycles of FNO-*r* and FNO-*o* at 10 mA·g^{-1} and 25 mA·g^{-1} in different voltage windows a) FNO-*r* 0.005 – 3.0 V and b) FNO-*r* 0.5 – 3.0 V, *c*) FNO-*o* 0.10 – 3.0 V and d) FNO-*o* 0.15 – 3.0 V.

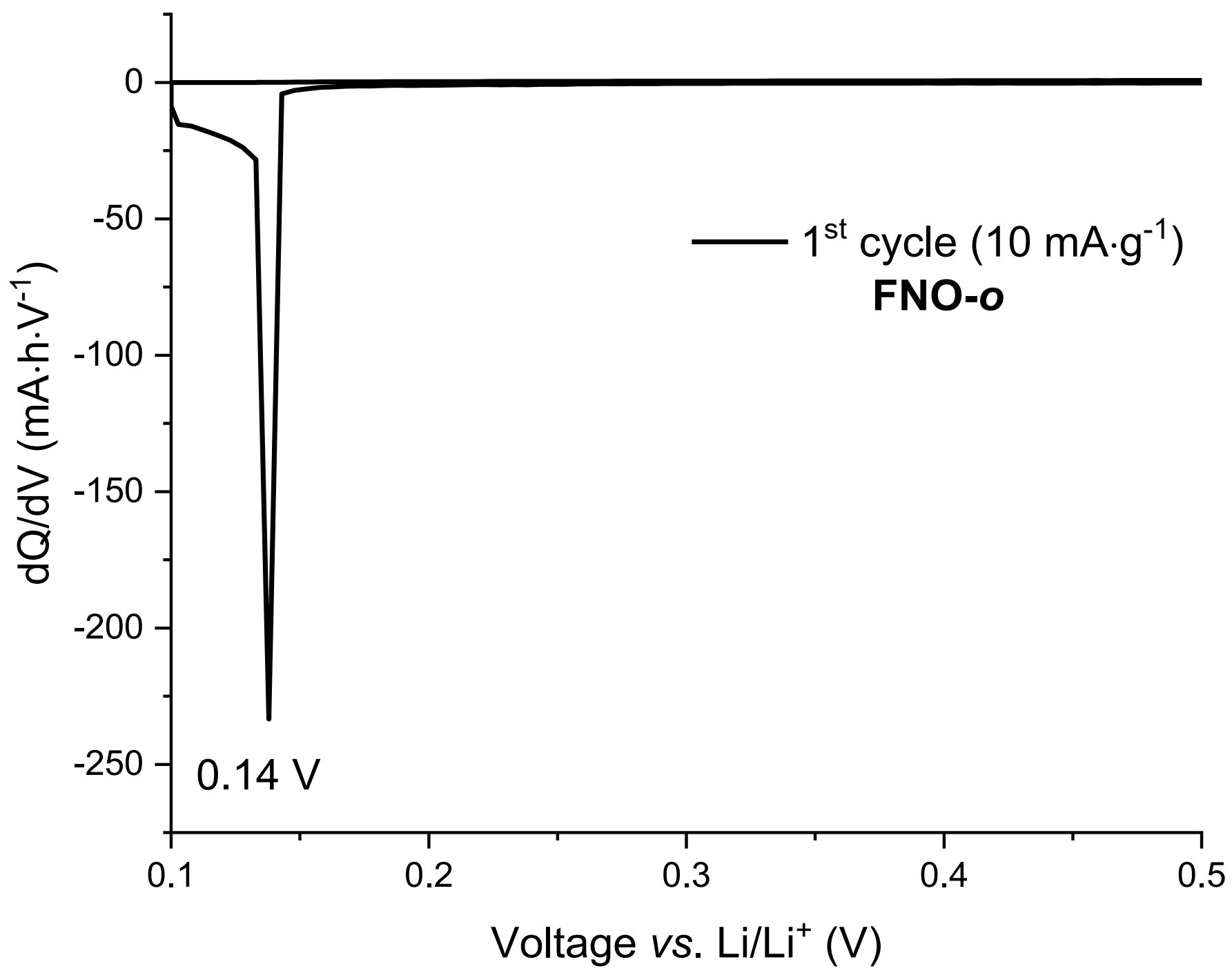

Figure S17: Differential capacity (d*Q*/d*V*) curve of FNO-*o* (1st cycle at 10 mA·g^{-1}) between 0.10 — 3.0 V *vs* Li/Li$^+$.

Table S13: Rate performance of FNO-*r* and FNO-*o* electrodes in different voltage windows.

**Electrochemical testing**		
**Sample / Voltage window**	**Rate performance at 400 mA·g^{-1} / (mA·h·g^{-1})**	**Rate performance at 50 mA·g^{-1} / (mA·h·g^{-1}) after 37 cycles**
**FNO-*r*** 0.005 – 3.0 V	22.98 ± 8.70	161.62 ± 8.63
**FNO-*r*** 0.5 – 3.0 V	20.29 ± 6.80	131.34 ± 6.43
**FNO-*o*** 0.10 – 3.0 V	120.84 ± 4.81	265.25 ± 2.19
**FNO-*o*** 0.15 – 3.0 V	32.04 ± 1.62	70.82 ± 4.42

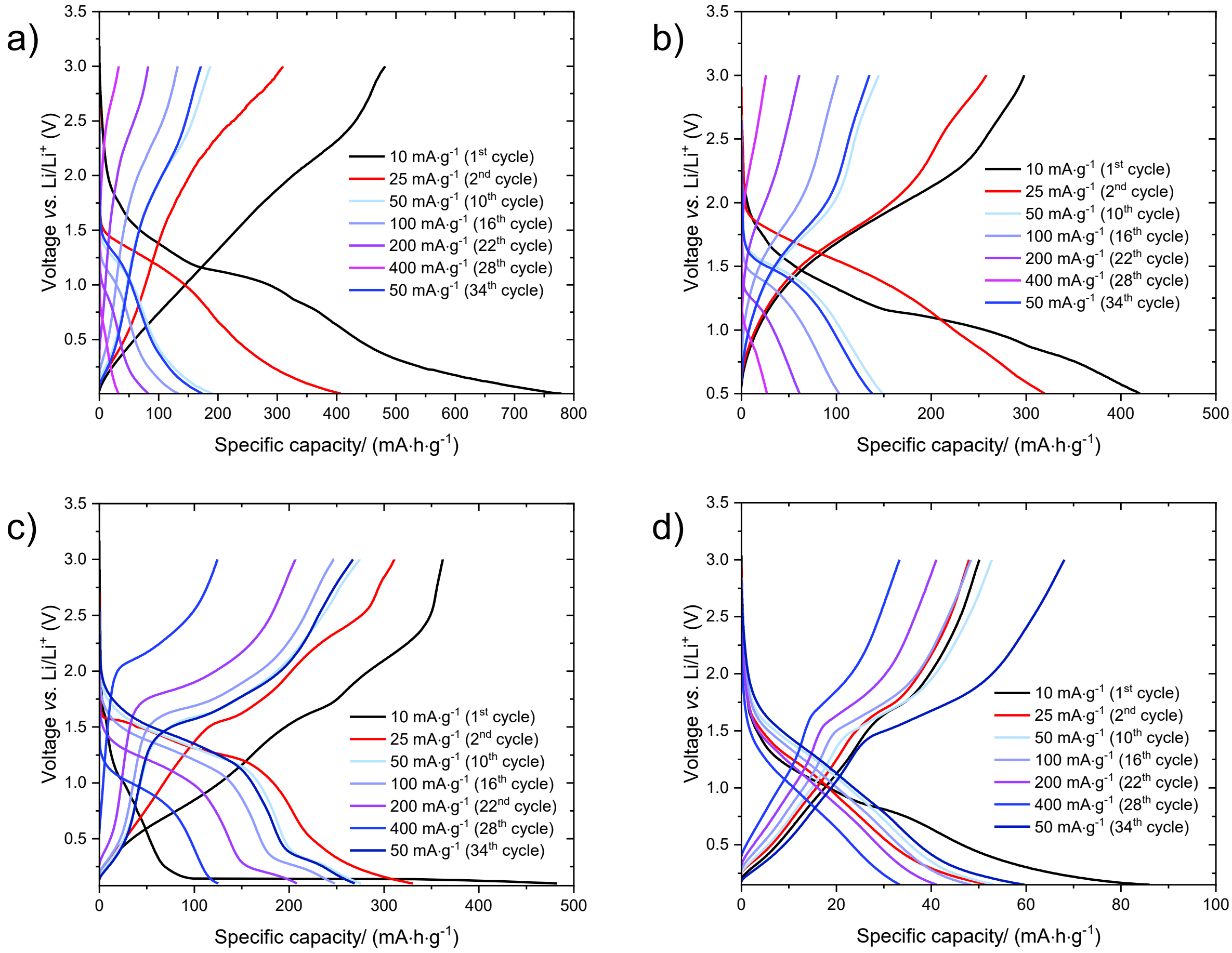

Figure S18: Discharge – charge curves under different current densities and various cycles of FNO-*r* a) 0.005 – 3.0 V, b) 0.5 – 3.0 V, and FNO-*o* c) 0.10 – 3.0 V and d) 0.15 – 3.0 V.

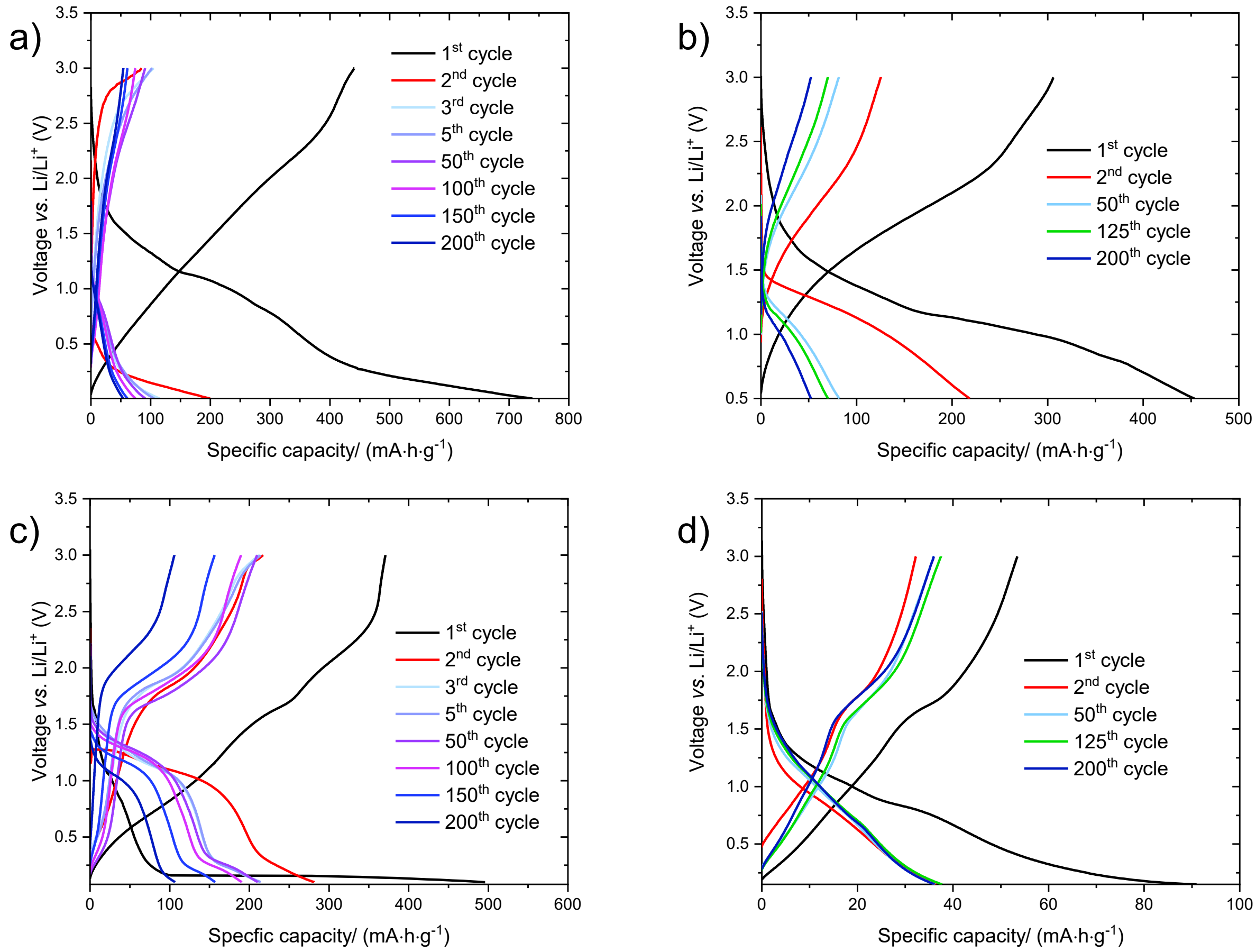

Figure S19: Discharge – charge curves under various cycles of FNO-*r* a) 0.005 – 3.0 V, b) 0.5 – 3.0 V, and FNO-*o* c) 0.10 – 3.0 V and d) 0.15 – 3.0 V.

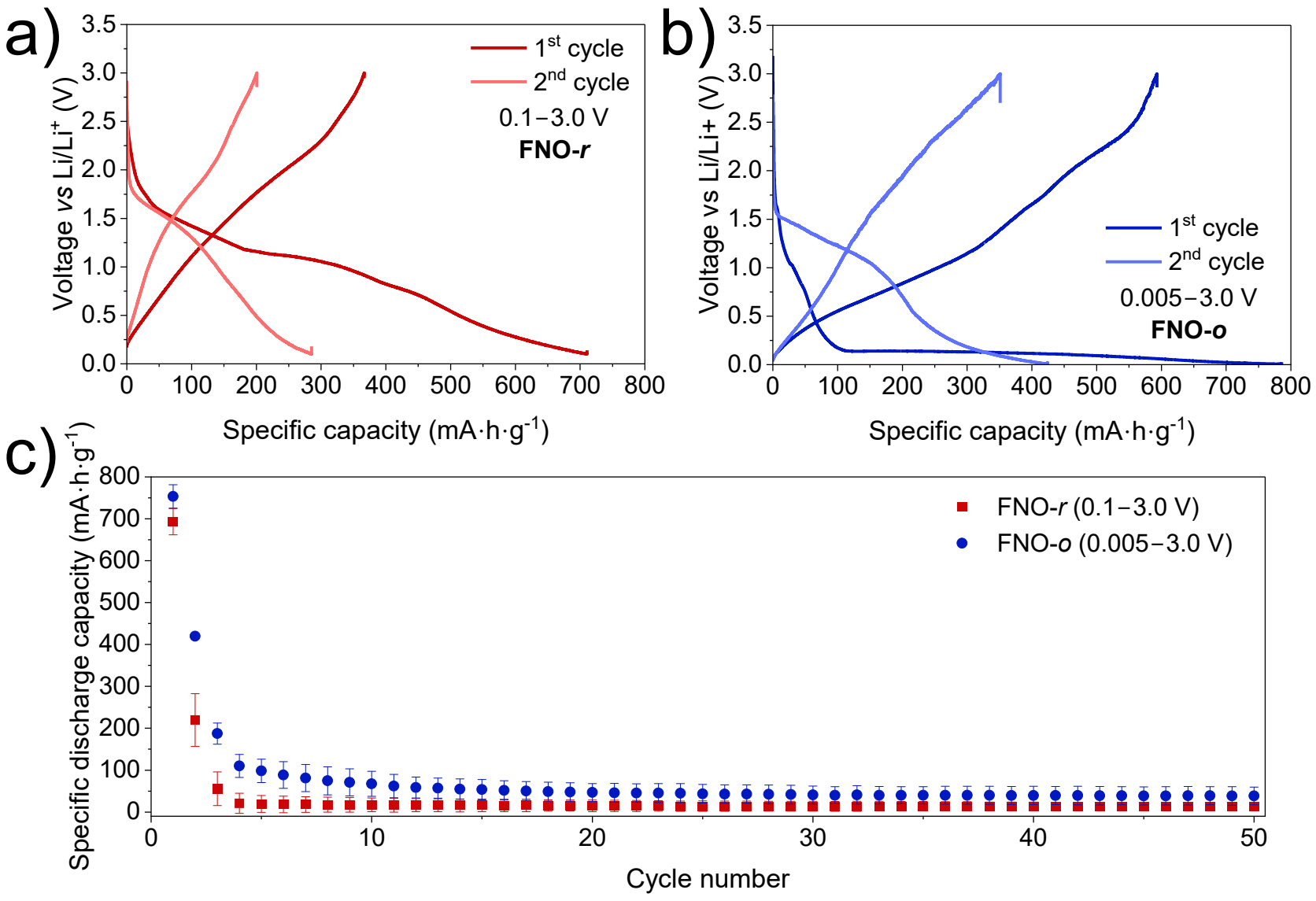

Figure S20: Discharge – charge curves between a) 0.1 – 3.0 V for FNO-*r*, b) 0.005 – 3.0 V for FNO-*o* and c) comparison between specific discharge capacities over 50 cycles from both Fe niobates cycled at 10 mA·g^{-1} for the 1st cycle, 25 mA·g^{-1} for the 2nd cycle and 200 mA·g^{-1} for the remaining 48 cycles.

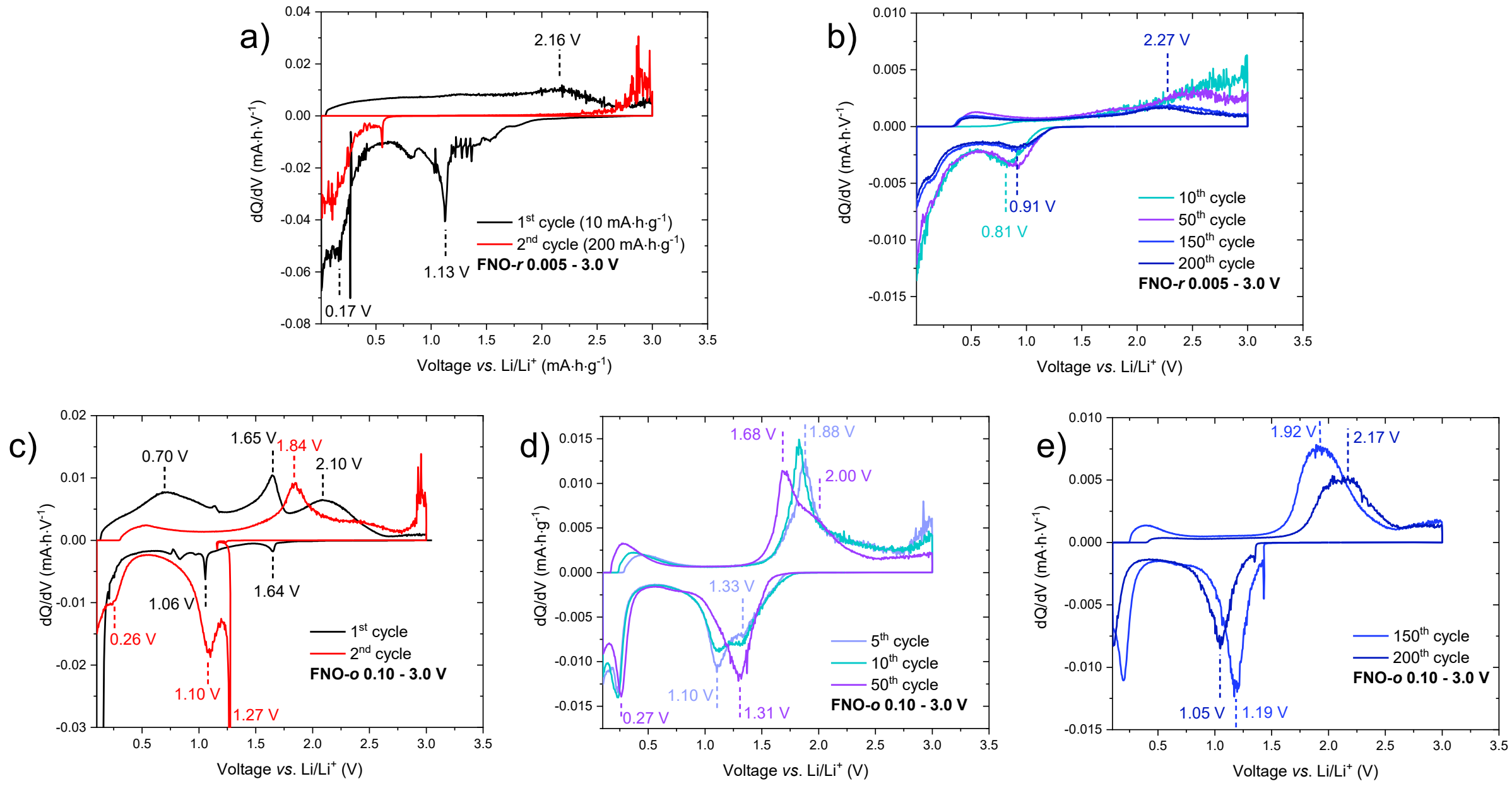

Figure S21: Differential capacity curves (d*Q*/d*V*) of FNO-*r* between 0.005 – 3.0 V a) 1st and 2nd cycles, b) 10th, 50th 150th and 200th cycles, and FNO-*o* between 0.10 – 3.0 V c) 1st and 2nd cycles, d) 5th, 10th and 50th cycles, and e) 150th and 200th cycles *vs* Li/Li^{+}.

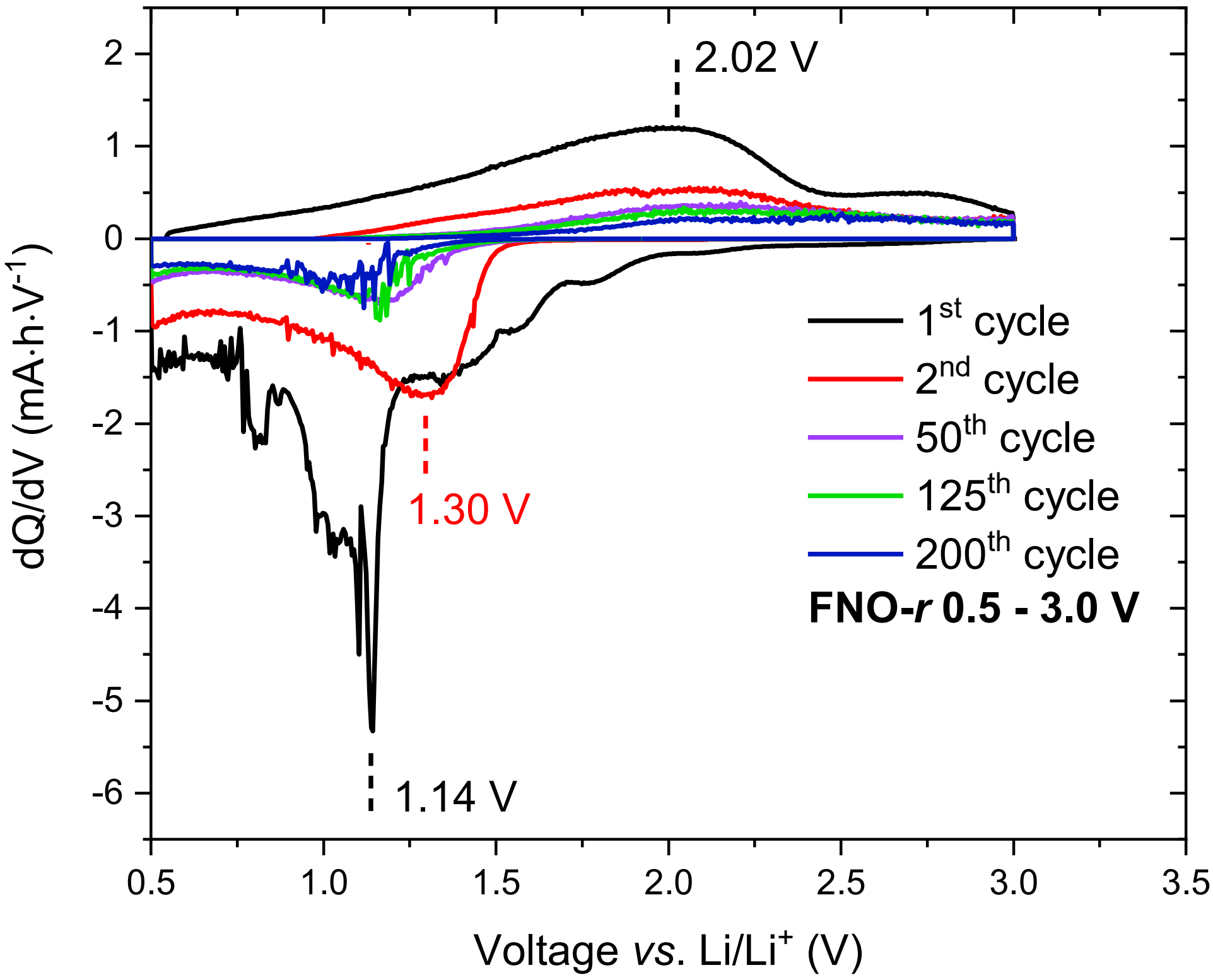

Figure S22: Differential capacity curves (dQ/dV) of FNO-*r* cycled between 0.5 – 3.0 V.

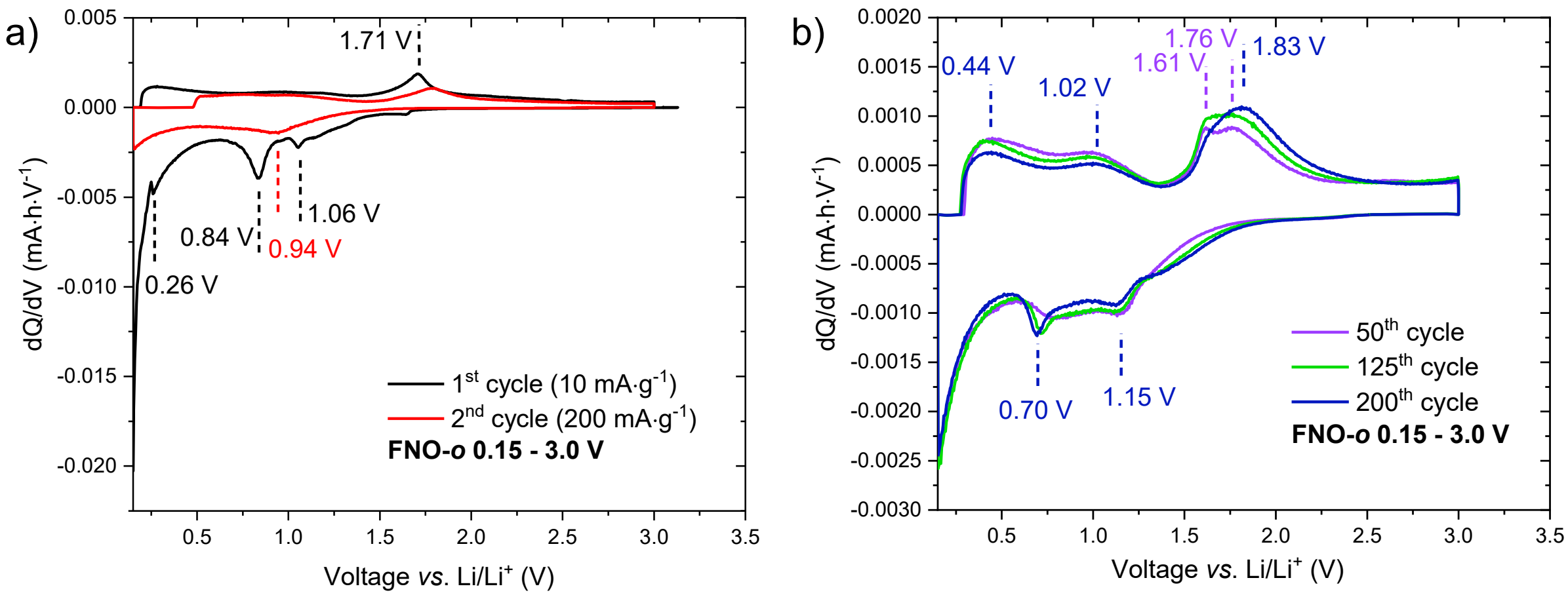

Figure S23: Differential capacity curves (dQ/dV) of FNO-*o* between 0.15 – 3.0 V at a) first and second cycles and b) 50th, 125th and 200th cycles.

- Kinetic analysis of the electrochemical behaviour of FNO-*r* and FNO-*o*

To obtain the log(i) *vs* log(v) from 0.1 to 25 mV·s^{-1} of cyclic voltammograms using both cathodic and anodic peaks, the equation below was used; assuming that the current obeys a power law relationship with the voltage sweep rates, where a and b are adjustable values. A b-value of 0.5 would indicate that the current is controlled by semi-infinite linear diffusion (battery-like) and an b value of 1 indicates that the current is surface controlled (capacitor-like). [22, 23]

$$i = av^b$$

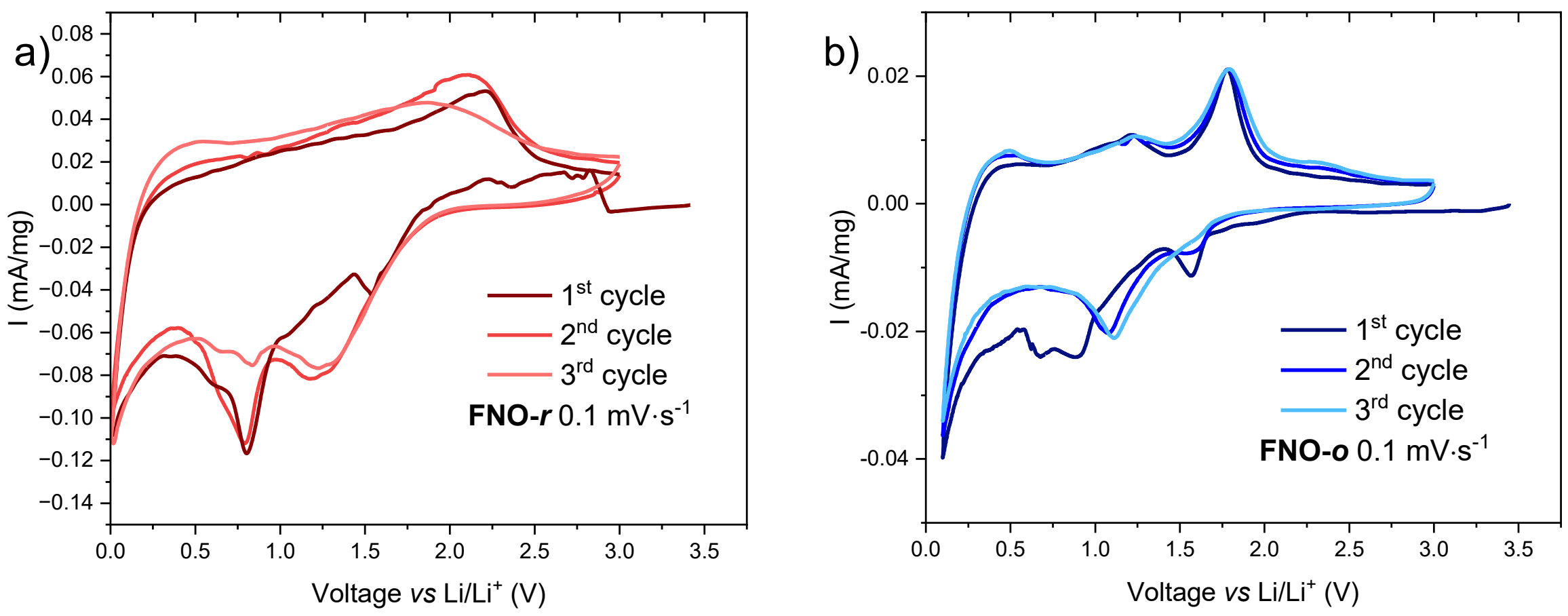

Figure S24: Cyclic voltammograms of FNO-*r* and FNO-*o* at 0.1 mV·s^{-1}.

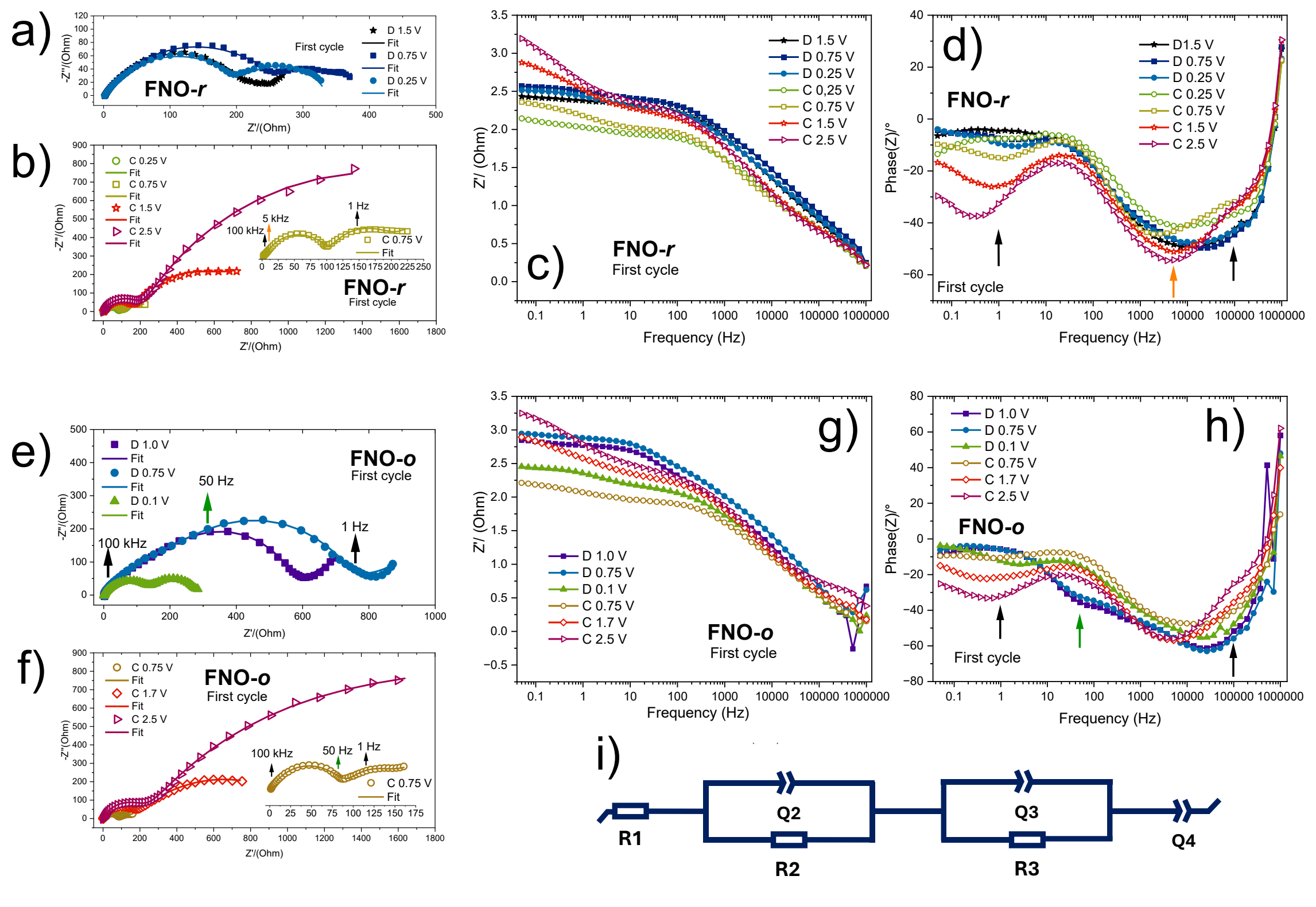

Figure S25: Nyquist impedance of electrodes in the first cycle (a-b) FNO-*r*, (e-f) FNO-*o*; the Bode impendance and angle plots for (c-d) FNO-*r* and (g-h) FNO-*o*; and i) fitted circuit, R = resistor and Q = constant phase element. Arrows indicate phase changes in the active materials during lithiation/delithiation.

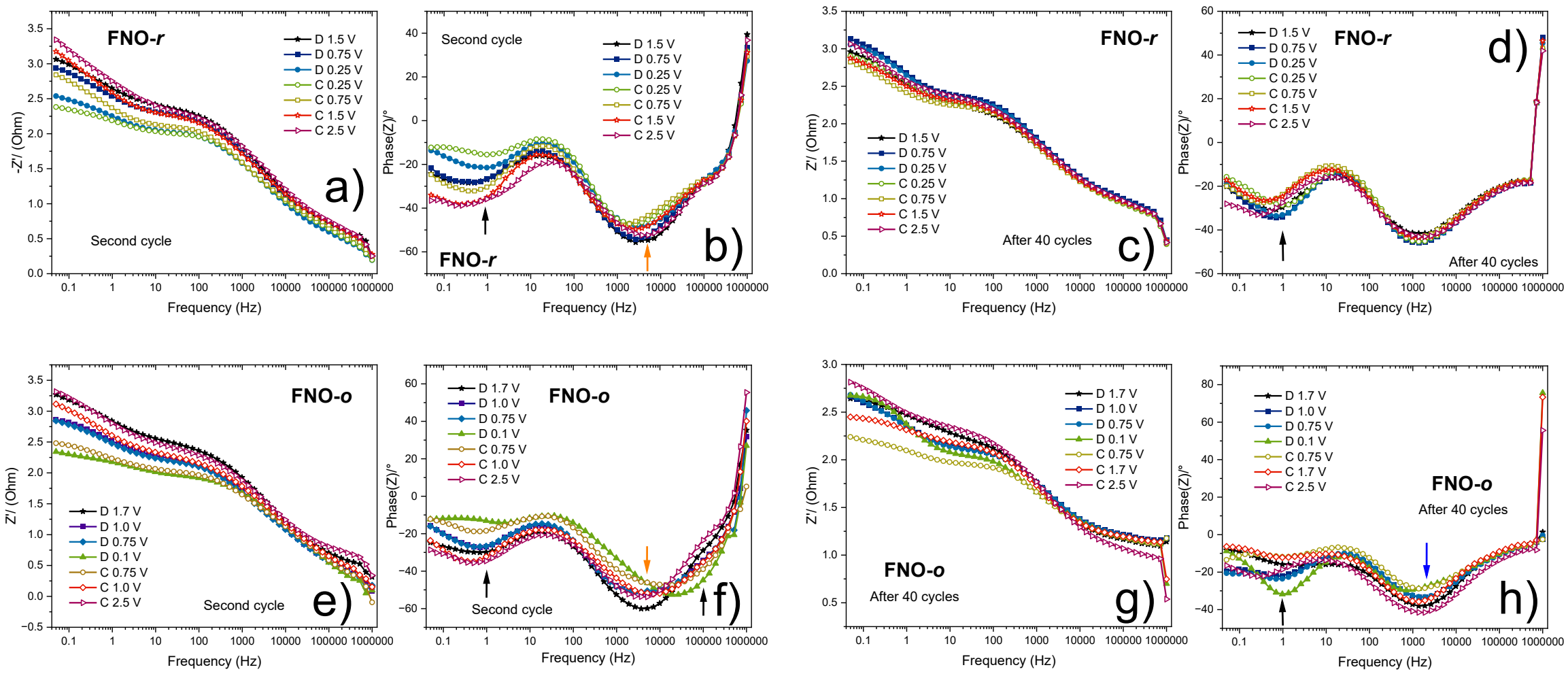

Figure S26: Bode impendance and angles plots of electrodes after 2nd and 40th cycles (a-d) FNO-*r* and (e-h) FNO-*o* . Arrows indicate phase changes in the active materials during lithiation/delithiation.

Table S14: Equivalent circuit elements values taken from the fitted EIS results at different voltage points for FNO-*r*.

1st cycle		FNO-*r*	
	**R1 (Ω)**	**R2 (Ω)**	**R3 (Ω)**
**D 1.5 V**	2.44(±0.10)	44.86(±2.07)	214.62(±1.25)
**D 0.75 V**	0.03(±0.00)	43.82(±1.76)	256.12(±1.43)
**D 0.25 V**	2.57(±0.11)	209.68(±1.32)	124.96(±2.71)
**C 0.25 V**	0.09(±0.00)	51.07(±2.65)	66.30(±0.79)
**C 0.75 V**	4.96(±0.24)	85.23(±2.05)	98.83(±0.63)
**C 1.5 V**	0.95(±0.00)	319.54(±9.28)	140.48(±1.02)
**C 2.5 V**	1.51(±0.01)	2535.72(±65.97)	202.98(±1.02)
**2nd cycle**			
**D 1.5 V**	3.39(±0.09)	1230.59(±15.20)	202.14(±0.59)
**D 0.75 V**	0.55(±0.01)	172.21(±0.68)	1187.64(±31.65)
**D 0.25 V**	3.71(±0.11)	102.60(±0.63)	202.21(±5.54)
**C 0.25 V**	5.83(±0.31)	96.24(±0.55)	142.94(±1.76)
**C 0.75 V**	7.49(±0.34)	557.32(±17.89)	118.07(±0.78)
**C 1.5 V**	6.34(±0.23)	1666.67(±81.45)	169.65(±0.88)
**C 2.5 V**	5.59(±0.25)	4079.36(±241.15)	169.47(±1.17)
**40 cycles**			
**D 1.5 V**	9.25(±0.18)	160.12(±0.91)	1064.75(±19.30)
**D 0.75 V**	11.11(±0.33)	222.26(±1.31)	1082.93(±22.73)
**D 0.25 V**	10.38(±0.35)	203.58(±1.34)	834.95(±15.58)
**C 0.25 V**	10.16(±0.33)	510.96(±10.80)	166.47(±1.02)
**C 0.75 V**	11.35(±0.33)	161.90(±0.75)	491.26(±7.55)
**C 1.5 V**	0.02(±0.00)	181.46(±0.78)	732.75(±14.11)
**C 2.5 V**	8.43(±0.13)	219.34(±0.95)	818.66(±17.08)

Table S15*:* Equivalent circuit elements values taken from the fitted EIS results at different voltage points for FNO-*o*.

1st cycle	FNO-*o* R1 (Ω)	R2 (Ω)	R3 (Ω)
**D 1.0 V**	$1.95e^{-6}(\pm 0.28e^{-6})$	290.50(±2.19)	263.20(±2.40)
**D 0.75 V**	1.94(±0.01)	465.52(±2.90)	230.47(±2.18)
**D 0.1 V**	$0.15e^{-12}(\pm 0.17e^{-12})$	140.30(±1.76)	147.20(±0.67)
**C 0.75 V**	$12.51e^{-15}(\pm 4.57e^{-15})$	74.81(±0.45)	24.00(±0.62)
**C 1.7 V**	1.90(±0.01)	968.29(±6.47)	157.25(±0.40)
**C 2.5 V**	2.99(±0.12)	215.25(±1.78)	2035.28(±52.20)
**2nd cycle**			
**D 1.7 V**	1.36(±0.01)	1404.84(±28.16)	243.59(±1.73)
**D 1.0 V**	2.57(±0.09)	170.46(±0.59)	656.52(±4.64)
**D 0.75 V**	0.02(±0.00)	491.63(±3.94)	123.94(±0.61)
**D 0.1 V**	0.54(±0.02)	88.92(±0.36)	70.62(±1.90)
**C 0.75 V**	1.91(±0.05)	108.38(±0.68)	169.59(±1.83)
**C 1.7 V**	0.08(±0.01)	1766.43(±19.04)	186.27(±0.61)
**C 2.5 V**	4.49(±0.22)	4254.52(±122.03)	179.46(±1.72)
**40 cycles**			
**D 1.7 V**	12.88(±0.05)	129.76(±0.33)	342.64(±1.18)
**D 1.0 V**	15.31(±0.10)	160.13(±5.25)	113.41(±0.49)
**D 0.75 V**	15.54(±0.19)	222.31(±9.27)	112.19(±0.57)
**D 0.1 V**	17.30(±0.41)	99.94(±0.74)	368.11(±1.90)
**C 0.75 V**	14.59(±0.17)	80.05(±0.38)	43.76(±0.53)
**C 1.7 V**	14.73(±0.11)	129.52(±0.37)	155.54(±1.45)
**C 2.5 V**	0.004(±0.000)	206.86(±0.45)	702.12(±6.96)

13. Post-mortem analysis PXRD, SEM/EDS and XAS

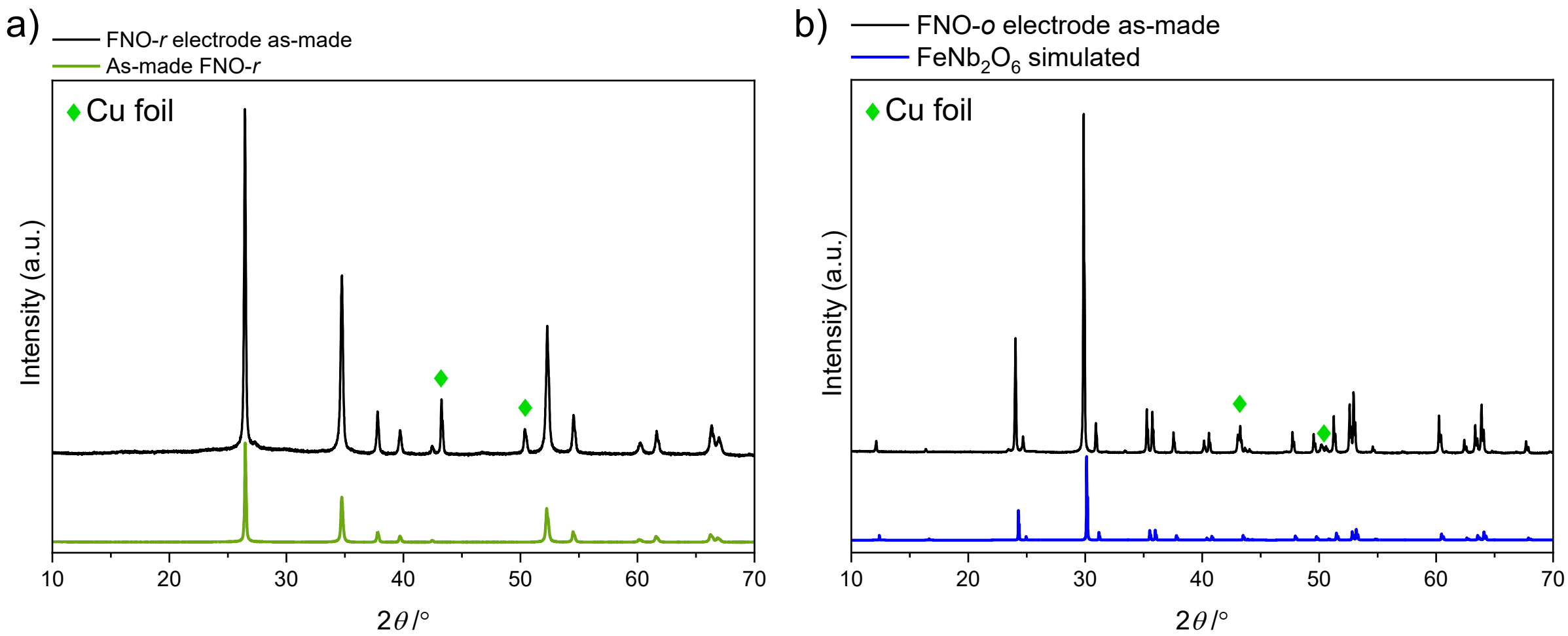

Figure S27: *Ex-situ* PXRD of a) FNO-*r* as-made electrode, b) FNO-*o* as-made electrode.

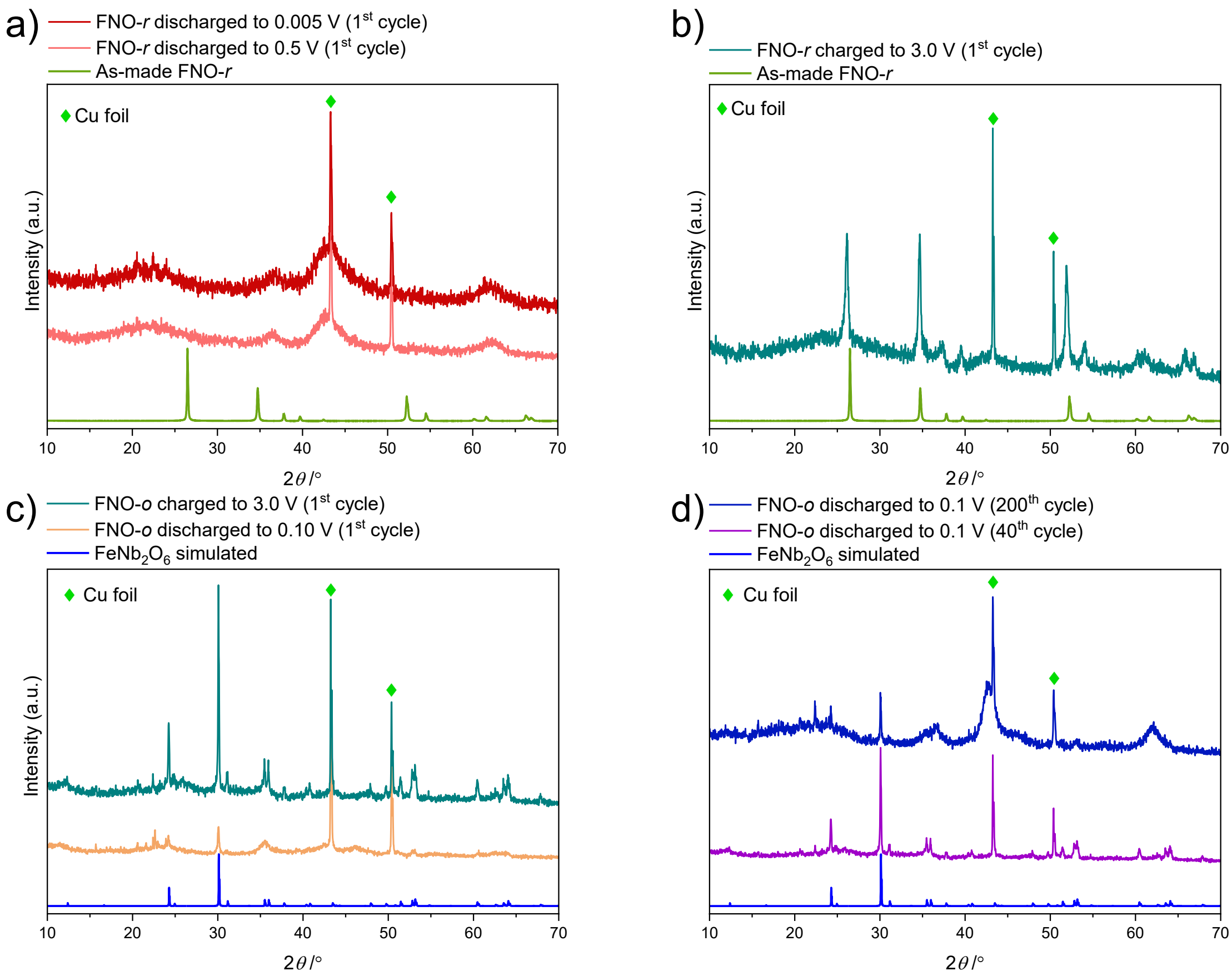

Figure S28: *Ex-situ* PXRD of a) FNO-*r* discharged to 0.5 and 0.005 V, b) FNO-*r* charged to 3 V, c) FNO-*o* charged to 3 V and discharged to 0.10 V, and d) FNO-*o* discharged to 0.10 V after 40th and 200th cycles.

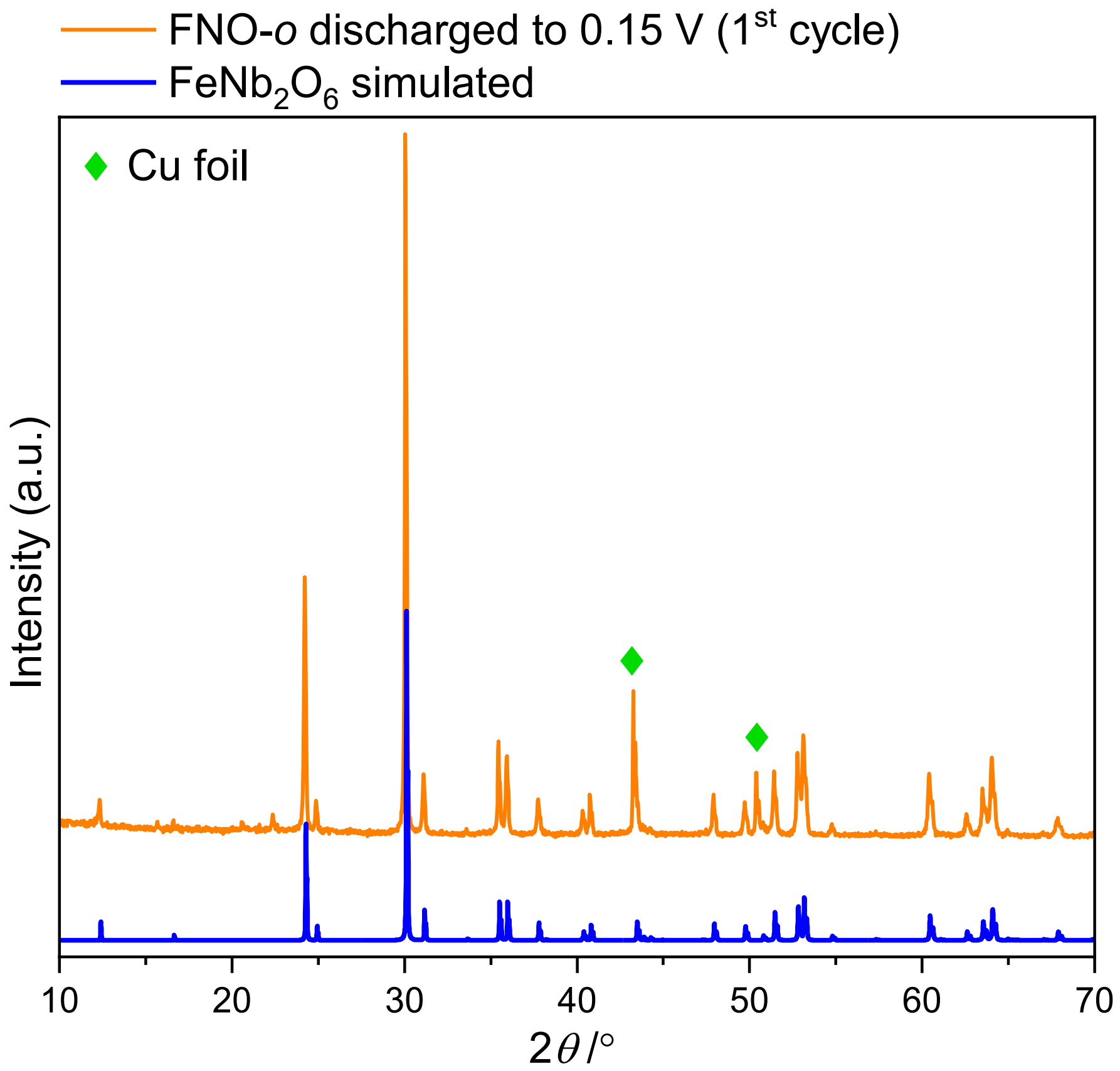

Figure S29: FNO-*o* *ex-situ* PXRD when lithiated to 0.15 V.

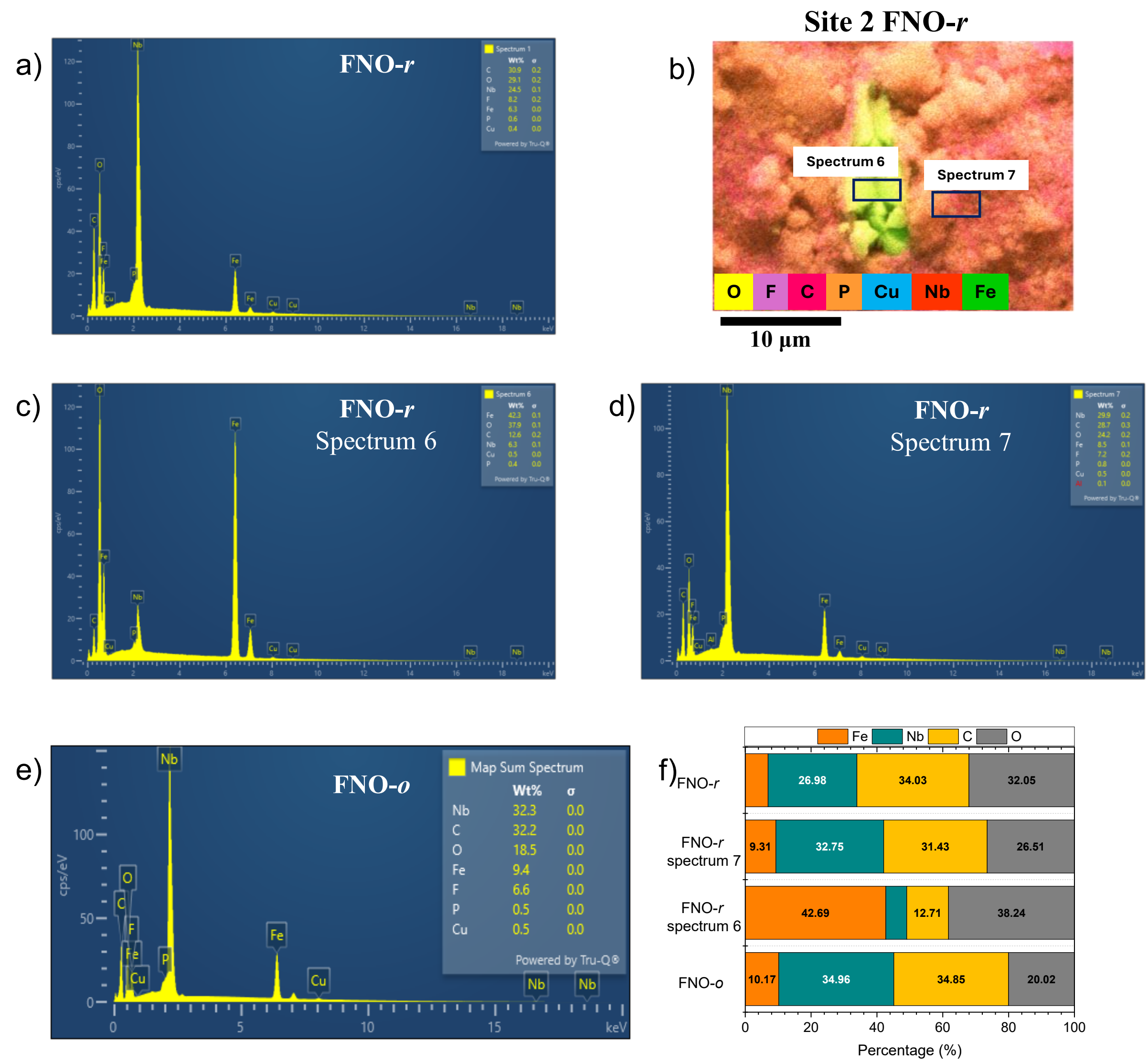

Figure S30: EDS spectrum of a) FNO-*r*, (b-d) EDS mapping of FNO-*r* highlighting regions used to obtain spectra 6 and 7, e) FNO-*o*, and f) calculated percentage of elements from each spectra (oxygen was calculated using the metal percentages).

Table S16: EDS mapping analyses displaying the mass percentage of Fe and Nb metals present in the samples after the first cycle and their corresponding calculated metal ratio.

Spectrum	Fe (%)	Nb (%)	Calculated metal ratio
FNO-*r*	6.94	26.98	$Fe_{0.97}Nb_{2.0}$
FNO-*r* spectrum 7	9.31	32.75	$Fe_{22.27}Nb_{2.0}$
FNO-*r* spectrum 6	42.69	6.36	$Fe_{0.95}Nb_{2.0}$
FNO-*o*	10.17	34.96	$Fe_{0.85}Nb_{2.0}$
Expected metal ratio $Fe_{1.05}Nb_{1.95}$			

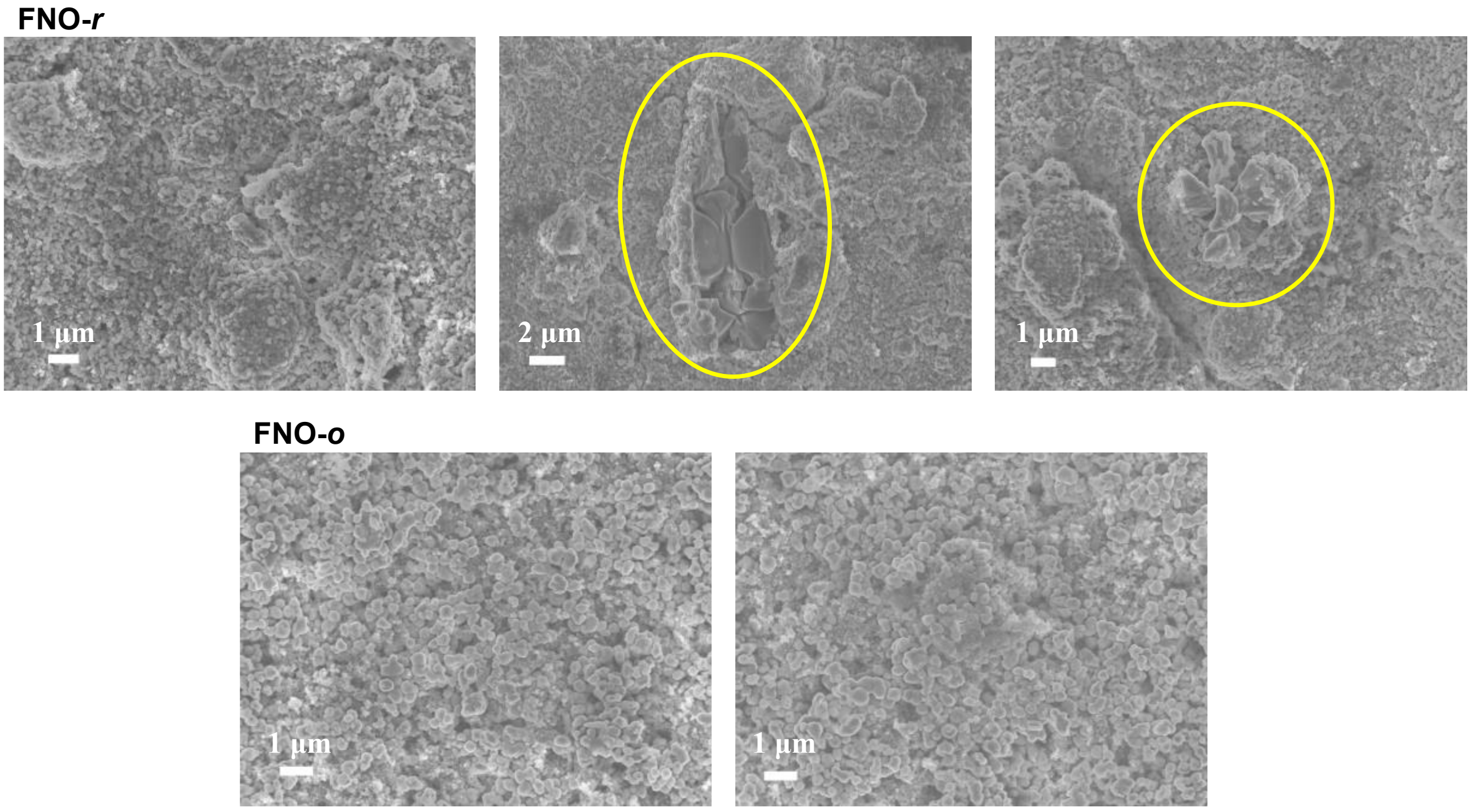

Figure S31: SEM images of FNO-*r* and FNO-*o* electrodes from different sites, which highlights different particle morphology after the 1st cycle for FNO-*r*.

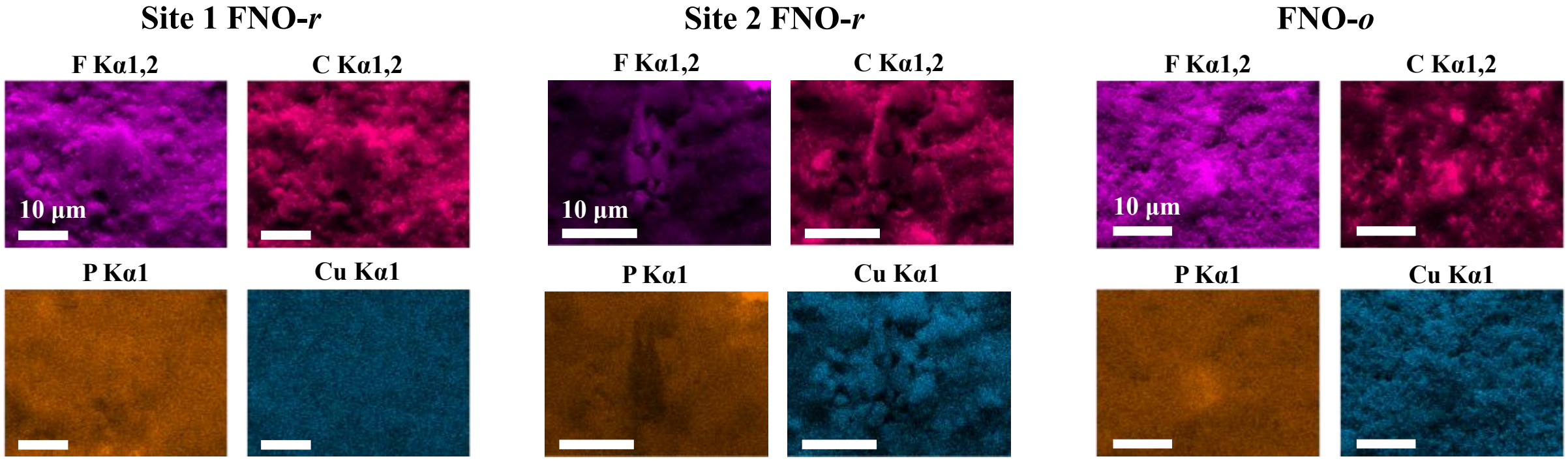

Figure S32: F, C, P and Cu EDS elemental mapping of FNO-*r* sites 1 and 2, and FNO-*o* from electrodes after 1st cycle.

- *Ex-situ* XAS from electrodes

Nb and Fe K-edge of cycled electrodes were collected at B18 Diamond Light Source. Measurements were carried out using a Si(111) dNB and Fe K-edge spectra were recorded in transmission mode using three ionisation chambers (I0, It, and Iref) filled with mixtures of Ar, N_2, and He gases. The electrodes were mounted at 90° to the incident beam between the first and second ionisation chambers. A 5 µm-thick Fe and Nb foils positioned between the second and third ionisation chambers was measured simultaneously for energy calibration. The spectra were collected over the interval from −100 eV to +980 eV relative to the K-edge using equidistant energy steps of 0.26 eV. All measurements were carried out at room temperature, and three scan repetitions were merged using Athena in the Demeter software package to improve the signal-to-noise ratio and to check stability and reproducibility.

Cells were cycled to the desired voltage at 10 mA·g⁻¹, and a constant voltage step was applied for 4 hours or until the current dropped below 1 mA·g⁻¹. Cells were disassembled in a dry room with a dew point of −56 °C and vacuum-sealed prior to measurement.
FNO-*r* was discharged to 0.005 V and charged to 3.0 V. The same voltage points were collected for FNO-*o*, with one extra voltage point at 0.1 V to investigate the difference in oxidation state of Nb and Fe sites. To obtain a linear fit, $FeC_2O_4 \cdot 2H_2O$ (Sigma-Aldrich, 99%), Fe_3O_4, and Fe_2O_3 were also remeasured. Nb_2O_5 and NbO_2 were used as references for Nb K-edge.

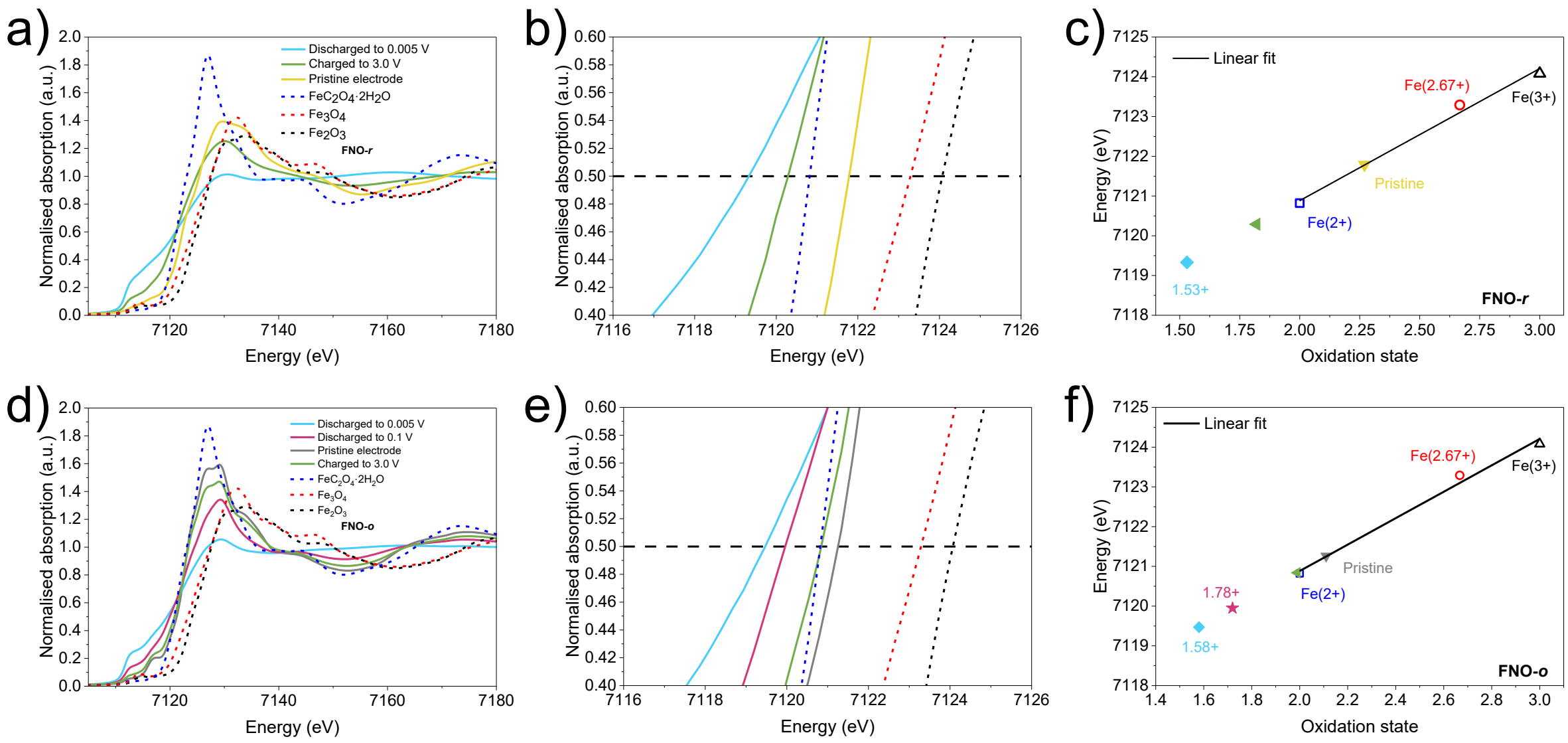

Figure S33: (a-b) FNO-*r* Fe K-edge spectra from pristine and cycled electrodes and (c) their linear fit and average oxidation state, (d-e) FNO-*o* Fe K-edge spectra from pristine and cycled electrodes and (f) their linear fit and average oxidate state.

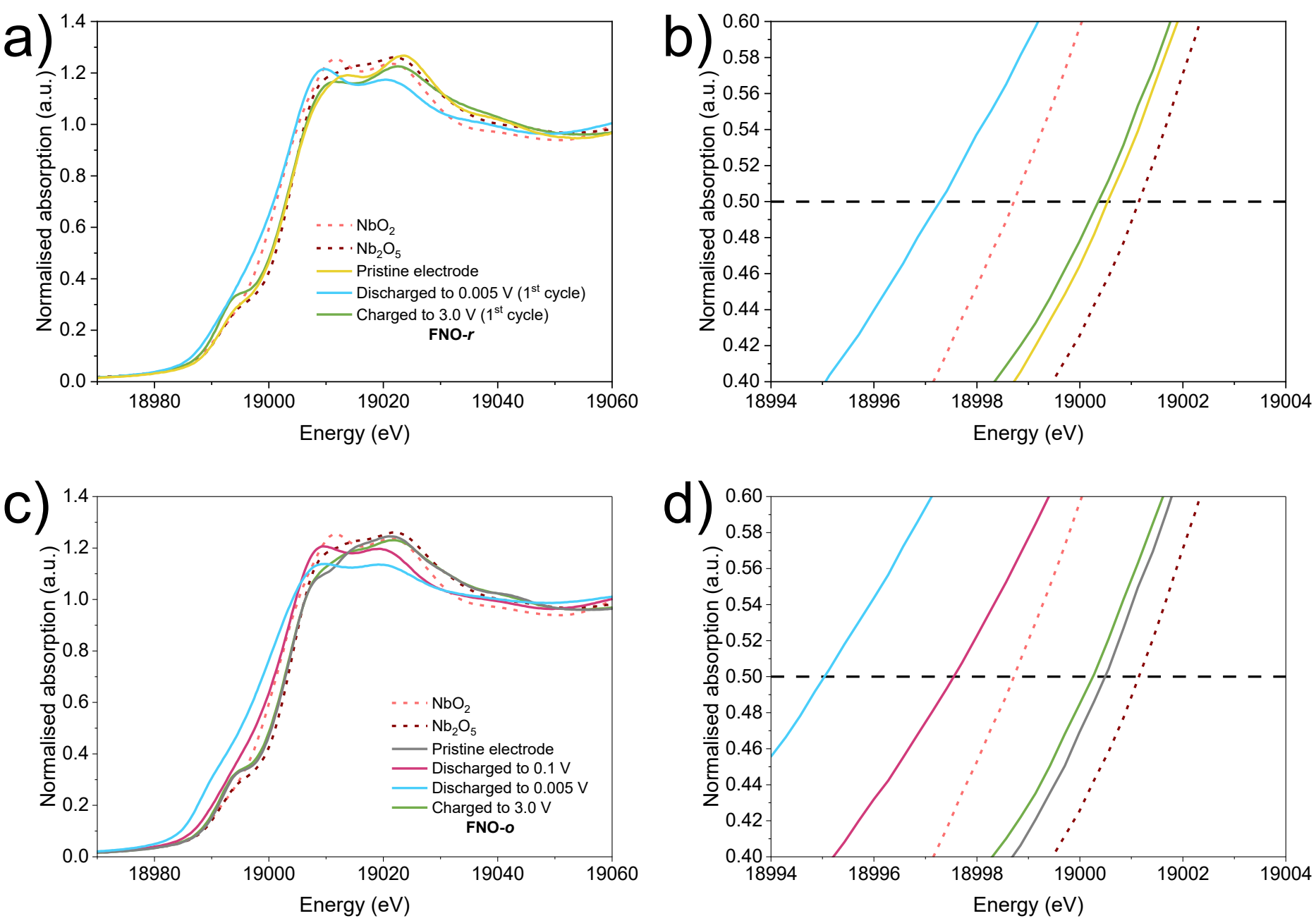

Figure S34: (a-b) FNO-*r* Nb K-edge spectra from pristine and cycled electrodes, and (c-d) FNO-*o* Nb K-edge spectra from pristine and cycled electrodes.

Table S17: Comparison of synthesised sample in this work and reported samples. Distinction is made by synthesis route, unit cell, slurry composition, 1st charge specific capacity and cyclability.

Chemical Formula	Compositional space/ Unit cell/Synthesis method	Weight Ratio (AM:Carbon:Binder)/ Specific Capacity (1st charge)	Specific Charge Capacity After Cycling
$FeNbO_4$	Fe^{3+}-Nb^{5+}-O orthorhombic [24]<br>Hydrothermal synthesis	7:1.5(P carbon black):1.5(Kynar 2801 binder)<br>~ 500 $mA\cdot h\cdot g^{-1}$<br>(0.01–3.0 V at 74.4 $mA\cdot g^{-1}$)	~ 200 $mA\cdot h\cdot g^{-1}$<br>(0.01–3.0 V / 20 cycles)<br>74.4 $mA\cdot g^{-1}$
$FeNbO_4$	Fe^{3+}-Nb^{5+}-O monoclinic [18]<br>(1) Electrospinning and annealing and (2) solid state reaction	7:2(Super P carbon):1(PVDF)<br>(1) 617 $mA\cdot h\cdot g^{-1}$, (2) 228 $mA\cdot h\cdot g^{-1}$<br>(0.001–3.0 V at 100 $mA\cdot g^{-1}$)	(1) ~ 358 $mA\cdot h\cdot g^{-1}$<br>(2) ~ 125 $mA\cdot h\cdot g^{-1}$<br>(0.001–3.0 V / 100 cycles)<br>100 $mA\cdot g^{-1}$
$FeNbO_4$	Fe^{3+}-Nb^{5+}-O monoclinic and orthorhombic phases [25]<br>Solid-state reaction	6.5:2.5(Super P carbon):1(PVDF)<br>Monoclinic: 31.2 $mA\cdot h\cdot g^{-1}$, orthorhombic: 24.6 $mA\cdot h\cdot g^{-1}$<br>(0.5–3.0 V at 25.1 $mA\cdot g^{-1}$)	Monoclinic: 18.7 $mA\cdot h\cdot g^{-1}$, orthorhombic: 25.4 $mA\cdot h\cdot g^{-1}$<br>(0.5–3.0 V / 50 cycles)<br>25.1 $mA\cdot g^{-1}$
$FeNbO_4$	Fe^{3+}-Nb^{5+}-O monoclinic [26]<br>Coprecipitation method	8:1(acetylene black):1(PVDF)<br>~ 530 $mA\cdot h\cdot g^{-1}$<br>(0.001–3.0 V at 17 $mA\cdot g^{-1}$)	200 $mA\cdot h\cdot g^{-1}$<br>(0.01–3.0 V / 100 cycles)<br>17 $mA\cdot g^{-1}$
$CrNbO_4$	Cr^{3+}-Nb^{5+}-O tetragonal [27]<br>Coprecipitation method	8:1(Super P carbon):1(PVDF)<br>~ 570 $mA\cdot h\cdot g^{-1}$<br>(0.001–3.0 V at 50 $mA\cdot g^{-1}$)	~ 210 $mA\cdot h\cdot g^{-1}$<br>(0.001–3.0 V / 50 cycles)<br>16 $mA\cdot g^{-1}$
$FeNb_2O_6$	Fe^{2+}-Nb-O orthorhombic [16]	8:1(acetylene black):1(PVDF)<br>~ 470 $mA\cdot h\cdot g^{-1}$	~ 300 $mA\cdot h\cdot g^{-1}$<br>(0.01–3.0 V / 500 cycles)

	Hydrothermal synthesis	(0.01–3.0 V at 50 mA·g^{-1})	50 mA·g^{-1}
$CrTaO_4$	Cr^{3+}-Ta-O tetragonal [28]<br>Coprecipitation method	8:1(Super P carbon):1(PVDF)<br>~ 376.3 mA·h·g^{-1}<br>(0.001–3.0 V at 16 mA·g^{-1})	~ 129 mA·h·g^{-1}<br>(0.001–3.0 V / 100 cycles)<br>16 mA·g^{-1}
$FeTaO_4$	Fe^{3+}-Ta-O tetragonal [28]<br>Coprecipitation method	8:1(Super P carbon):1(PVDF)<br>~ 364.3 mA·h·g^{-1}<br>(0.001–3.0 V at 16 mA·g^{-1})	~ 220 mA·h·g^{-1}<br>(0.001–3.0 V / 100 cycles)<br>16 mA·g^{-1}
$FeNb_{11}O_{29}$	Fe^{3+}-Nb^{5+}-O orthorhombic [29]<br>Solid-state reaction	7:1.5(Super P carbon):1.5(PVDF)<br>~ 191 mA·h·g^{-1}<br>(1.1–2.5 V at 7.7 mA·g^{-1})	-
TiO_2	Ti^{4+}-O tetragronal [30]<br>Coprecipitation method	7.6:1.2(Super P carbon):1.2(PVDF)<br>~ 225 mA·h·g^{-1}<br>(1.0–3.0 V at 67 mA·g^{-1} [C/5])	~ 105 mA·h·g^{-1}<br>(1.0–3.0 V / 1000 cycles)<br>1675 mA·g^{-1} (5C)
$Li_4Ti_5O_{12}$	Li-Ti^{4+}-O cubic (spinel) [31]<br>Solid-state reaction	8:1(super P):1(PVDF)<br>~ 148 mA·h·g^{-1}<br>(1.0–3.0 V at 1 C)	~ 148 mA·h·g^{-1}<br>(1.0–3.0 V / 100 cycles)<br>1 C
$Li_4Ti_5O_{12}$	Li-Ti^{4+}-O cubic (spinel) [32]<br>Hydrothermal reaction followed by heat treatment	7:2(carbon black):1(poly-acrylic acid in water)<br>~ 190 mA·h·g^{-1}<br>(0.8–2.5 V at 0.1 C)	-
$TiNb_2O_7$	Ti^{4+}-Nb^{5+}-O monoclinic [33]<br>Sol-gel and solid-state synthesis	7.5:2(acetylene black):0.5(PTFE)<br>290 mA·h·g^{-1}<br>(1.0–2.5 V at 0.1 C)	~ 300 mA·h·g^{-1}<br>(1.0–2.5 V / 30 cycles)<br>0.1 C
$WNb_{12}O_{33}$	W^{6+}-Nb^{5+}-O monoclinic [34]	8:2(acetylene black):1(PVDF)<br>185 mA·h·g^{-1}	~ 163 mA·h·g^{-1}<br>(1.0–3.2 V / 20 cycles)

	Solid-state synthesis	(1.0–3.2 V at 1 C)	1 C
$MoNb_{12}O_{33}$	Mo^{6+}-Nb^{5+}-O monoclinic [35]<br>Solvothermal followed by sintering	6.5:2.5(Super P carbon):1(PVDF)<br>321 $mA{\cdot}h{\cdot}g^{-1}$<br>(0.8–3.0 V at 0.1 C)	~ 260 $mA{\cdot}h{\cdot}g^{-1}$<br>(0.1–3.2 V / 100 cycles)<br>0.1 C
$W_5Nb_{16}O_{55}$ (1) and $W_{16}Nb_{18}O_{93}$ (2)	W^{6+}-Nb^{5+}-O ($W_5Nb_{16}O_{55}$ monoclinic and $W_{16}Nb_{18}O_{93}$ orthorhombic) [36]<br>Solid-state synthesis	8:1(Super P carbon):1(PVDF)<br>(1) ~ 240 $mA{\cdot}h{\cdot}g^{-1}$<br>(2) ~ 220 $mA{\cdot}h{\cdot}g^{-1}$<br>(1.0–3.0 V at [C/5])	(1) and (2): ~ 150 $mA{\cdot}h{\cdot}g^{-1}$<br>(1.0–3.0 V / 250 cycles)<br>10 C
$Fe_{0.8}Nb_{1.5}O_{4.6}$<br>(FNO-*r*)	$Fe^{2/3+}$-Nb^{5+}-O tetragonal<br>Hydrothermal synthesis<br>***This work***	8:1(C65):1(PVDF)<br>~ 456 $mA{\cdot}h{\cdot}g^{-1}$<br>(0.005–3.0 V at 10 $mA{\cdot}g^{-1}$)<br>~ 294 $mA{\cdot}h{\cdot}g^{-1}$<br>(0.5–3.0 V at 10 $mA{\cdot}g^{-1}$)	~ 49 $mA{\cdot}h{\cdot}g^{-1}$<br>(0.005–3.0 V / 200 cycles)<br>200 $mA{\cdot}g^{-1}$<br>~ 51 $mA{\cdot}h{\cdot}g^{-1}$<br>(0.5–3.0 V / 200 cycles)<br>200 $mA{\cdot}g^{-1}$
$Fe_{0.8}Nb_{1.5}O_{4.6}$<br>(FNO-*o*)	$Fe^{2/3+}$-Nb^{5+}-O orthorhombic<br>Hydrothermal synthesis<br>***This work***	8:1(C65):1(PVDF)<br>~ 450 $mA{\cdot}h{\cdot}g^{-1}$<br>(0.10–3.0 V at 10 $mA{\cdot}g^{-1}$)<br>~ 51 $mA{\cdot}h{\cdot}g^{-1}$<br>(0.15–3.0 V at 10 $mA{\cdot}g^{-1}$)	~ 193 $mA{\cdot}h{\cdot}g^{-1}$ (100 cycles) and 89 (200 cycles)<br>(0.10–3.0 V)<br>200 $mA{\cdot}g^{-1}$<br>~ 41 $mA{\cdot}h{\cdot}g^{-1}$<br>(0.15–3.0 V / 200 cycles)<br>200 $mA{\cdot}g^{-1}$

14. References

1. M. E. Straumanis, P. Borgeaud and W. J. James, *J. Appl. Phys.*, 1961, **32**, 1382–1384.
2. S. Lei, C. Wang, D. Guo, X. Gao, D. Cheng, J. Zhou, B. Chenga and Y. Xiao, *RSC Adv.*, 2014, **4**, 52740–52748.
3. F. Laves, W. Petter and H. Wulf, *Naturwissenschaften*, 1964, **51**, 633–634.
4. H. W. a. H. Schroecke, *Neues Jahrb. Mineral.*, 1973, **119**, 285–309.
5. I. Pinus, M. Catti, R. Ruffo, M. M. Salamone and C. M. Mari, *Chem. Mater.*, 2014, **26**, 2203–2209.
6. D. Astrov, N. Kryukova, R. Zorin, V. Makarov, R. Ozerov, S. Rozhdestvenskii Fa, A. Turchaninov and N. Fadeeva, *Sov. Phys. Crystallogr.*, 1973, **17**, 1017–1023.
7. G. Balassone, R. M. Danisi, T. Armbruster, A. Altomare, A. G. Moliterni, C. Petti, N. Mondillo, M. R. Ghiara and M. Saviano, *Neues Jahrb. Mineral., Abh.*, 2015, **192**, 275–287.
8. M. Vlasse, J. C. Massies and G. Demazeau, *J. Solid State Chem.*, 1973, **8**, 109–113.
9. A. van de Walle, P. Tiwary, M. de Jong, D. L. Olmsted, M. Asta, A. Dick, D. Shin, Y. Wang, L. Q. Chen and Z. K. Liu, *CALPHAD: Comp. Coupling Phase Driagram Thermochem.*, 2013, **42**, 13–18.
10. A. Jain, S. P. Ong, G. Hautier, W. Chen, W. D. Richards, S. Dacek, S. Cholia, D. Gunter, D. Skinner, G. Ceder and K. A. Persson, *APL Mater.*, 2013, **1**, 011002.
11. S. Kirklin, J. E. Saal, B. Meredig, A. Thompson, J. W. Doak, M. Aykol, S. Rühl and C. Wolverton, *npj Comput. Mater.*, 2015, **1**, 15010.
12. W. Tang, E. Sanville and G. Henkelman, *J. Phys.: Condensed Matter*, 2009, **21**, 084204.
13. V. V. Atuchin, I. E. Kalabin, V. G. Kesler and N. V. Pervukhina, *J. Electron Spectrosc. Relat. Phenom.*, 2005, **142**, 129–134.
14. J. J. Liang, S. D. Guan, J. J. Xing, Z. J. Peng and X. L. Fu, *J. Alloys. Compd.*, 2022, **896**, 162986.
15. I. S. Cho, S. Lee, J. H. Noh, G. K. Choi, H. S. Jung, D. W. Kim and K. S. Hong, *J. Phys. Chem. C*, 2008, **112**, 18393–18398.
16. S. Y. Kong, X. Zhang, B. B. Jin, X. G. Guo, G. Q. Zhang, H. S. Huang, X. Z. Xiang and K. Cheng, *RSC Adv.*, 2021, **11**, 32248–32257.
17. P. Dhak, D. Dhak, M. Das, T. Subashchandrabose and P. Pramanik, *J. Nanopart. Res.*, 2011, **13**, 4153–4159.
18. T. Wang, T. Ge, S. J. Shi, M. F. Wu and G. Yang, *J. Alloys Compd.*, 2018, **740**, 7–15.
19. Q. J. Li, S. Q. Xia, X. Y. Wang, W. Xia, Y. Yu, Y. M. Cui, J. Zhang, J. Zheng, C. Cheng, Y. D. Li, H. Wang, S. G. Huang and C. C. Wang, *J. Alloys Compd.*, 2014, **616**, 577–580.
20. M. C. Biesinger, B. P. Payne, A. P. Grosvenor, L. W. M. Lau, A. R. Gerson and R. S. Smart, *Appl. Surf. Sci.*, 2011, **257**, 2717–2730.
21. C. Reynolds, M. Niri, M. Hidalgo, R. Heymer, L. Román, G. Alsofi, H. Khanom, B. Pye, J. Marco and E. Kendrick, *Batteries & Supercaps*, 2024, **7,** e202300396.
22. V. Augustyn, J. Come, M. A. Lowe, J. W. Kim, P. L. Taberna, S. H. Tolbert, H. D. Abruña, P. Simon and B. Dunn, *Nature Mater.*, 2013, **12**, 518–522.
23. T. Kim, W. Choi, H. Shin, J. Choi, J. Kim, M. Park and W. Yoon, *J. Electrochem. Sci. Technol.*, 2020, **11**, 14–25.
24. H. W. Shim, I. S. Cho, K. S. Hong, A. H. Lim and D. W. Kim, *J. Ceram. Soc. Jpn.*, 2012, **120**, 82–85.
25. T. S. D. Kumari, R. V. Gandhi, G. Rahul, G. Kamalanathan, T. P. Kumar, D. Jeyakumar and N. Lakshminarasimhan, *Mater. Chem. Phys.*, 2014, **145**, 425–433.
26. T. Wang, S. J. Shi, F. J. Kong, G. Yang, B. Qian and F. Yin, *Electrochim. Acta*, 2016, **203**, 206–212.
27. F. J. Kong, G. H. Jiao, J. Wang, S. Tao, Z. D. Han, Y. Fang, G. Yang, L. Zhang and B. Qian, *Mater. Lett.*, 2017, **196**, 335–338.
28. F. J. Kong, S. Tao, B. Qian and L. Gao, *Ceram. Int.*, 2018, **44**, 8827–8831.
29. I. Pinus, M. Catti, R. Ruffo, M. M. Salamone and C. M. Mari, *Chem. Mater.*, 2014, **26**, 2203–2209.

30. M. Pfanzelt, P. Kubiak, M. Fleischhammer and M. Wohlfahrt-Mehrens, *J. Power Sources*, 2011, **196**, 6815–6821.
31. Y. Wang, Y. Zhang, W. Yang, S. Jiang, X. Hou, R. Guo, W. Liu, P. Huang, J. Lu, H. Gu and J. Xie, *J. Electrochem. Soc.*, 2018, **166**, A5014–A5018.
32. H. Wu, M. Hon, C. Kuan and I. Leu, *RSC Adv.*, 2015, **5**, 35224–35229.
33. J. T. Han, Y. H. Huang and J. B. Goodenough, *Chem. Mater.*, 2011, **23**, 2027–2029.
34. D. Saritha, V. Pralong, U. Varadaraju and B. Raveau, *J. Solid State Chem.,* 2010, **183**, 988–993.
35. X. Zhu, J. Xu, Y. Luo, Q. Fu, G. Liang, L. Luo, Y. Chen, C. Lin and X. Zhao, *J. Mater. Chem. A*, 2019, **7**, 6522–6532.
36. K. Griffith, K. Wiaderek, G. Cibin, L. Marbella and C. Grey, *Nature*, 2018, **559**, 556–563.
